# Plant-derived nanovesicles: the intelligent nanoplatforms for therapeutics and drug delivery

**DOI:** 10.1186/s12951-026-04484-1

**Published:** 2026-05-02

**Authors:** Shuang Du, Dan Jia, Guohui Liang, Yonghui Dou

**Affiliations:** 1 School of Chinese Materia Medica, Guangzhou University of Chinese Medicine, Guangzhou, 510006 Guangdong PR China; 2 Guangzhou General Pharmaceutical Research Institute, Guangzhou, 510240 Guangdong PR China; 3 Research Team of Prevention and Treatment of Cerebral Hemorrhage Applying Chinese Medicine, The Second Affiliated Hospital of Guangzhou, University of Chinese Medicine, Guangzhou, 510120 Guangdong Province PR China; 4 The Second Affiliated Hospital of Guangzhou, University of Chinese Medicine, 111 Haizhu Zhong Road, Guangzhou, 510120 China

**Keywords:** Plant-derived nanovesicles, Engineering modification, Therapeutic potential, Drug delivery, Clinical application

## Abstract

Extracellular vesicles (EVs) are cell-secreted phospholipid bilayer vesicles that play a key role in intercellular communication by transporting molecular cargo and engaging in surface-level signaling. Due to their intrinsic biological features, EVs not only reflect the functional attributes of their originating cells but also hold promise as both therapeutic agent and natural carriers for targeted delivery. In recent years, plant-derived nanovesicles (PDNVs) containing bioactive molecules have attracted the attention of researchers because of their better biocompatibility, low immunogenicity, wide range of sources, and ability to act as natural therapeutic agents for diseases. PDNVs play an increasingly important role in human-plant interactions, as they are able to enter the human system and deliver effector molecules to cells, which in turn modulate cellular signaling pathways. PDNVs play a critical role in human health and disease. This review provides a comprehensive overview of PDNVs, encompassing their biogenesis, methods of isolation and purification, physicochemical characterization, stability, and storage strategies. It further explores their routes of administration, internalization, and biodistribution as therapeutic agents, highlighting their potential in the treatment of conditions such as inflammation, cancer, tissue regeneration, viral infections, liver and brain disorders, and osteoporosis. Lastly, the review examines current clinical applications of PDNVs and the key challenges hindering their broader implementation. We look forward to further exploration of the functions of PDNVs to facilitate their clinical translation and increase their benefits in humans.

**Graphical abstract:**

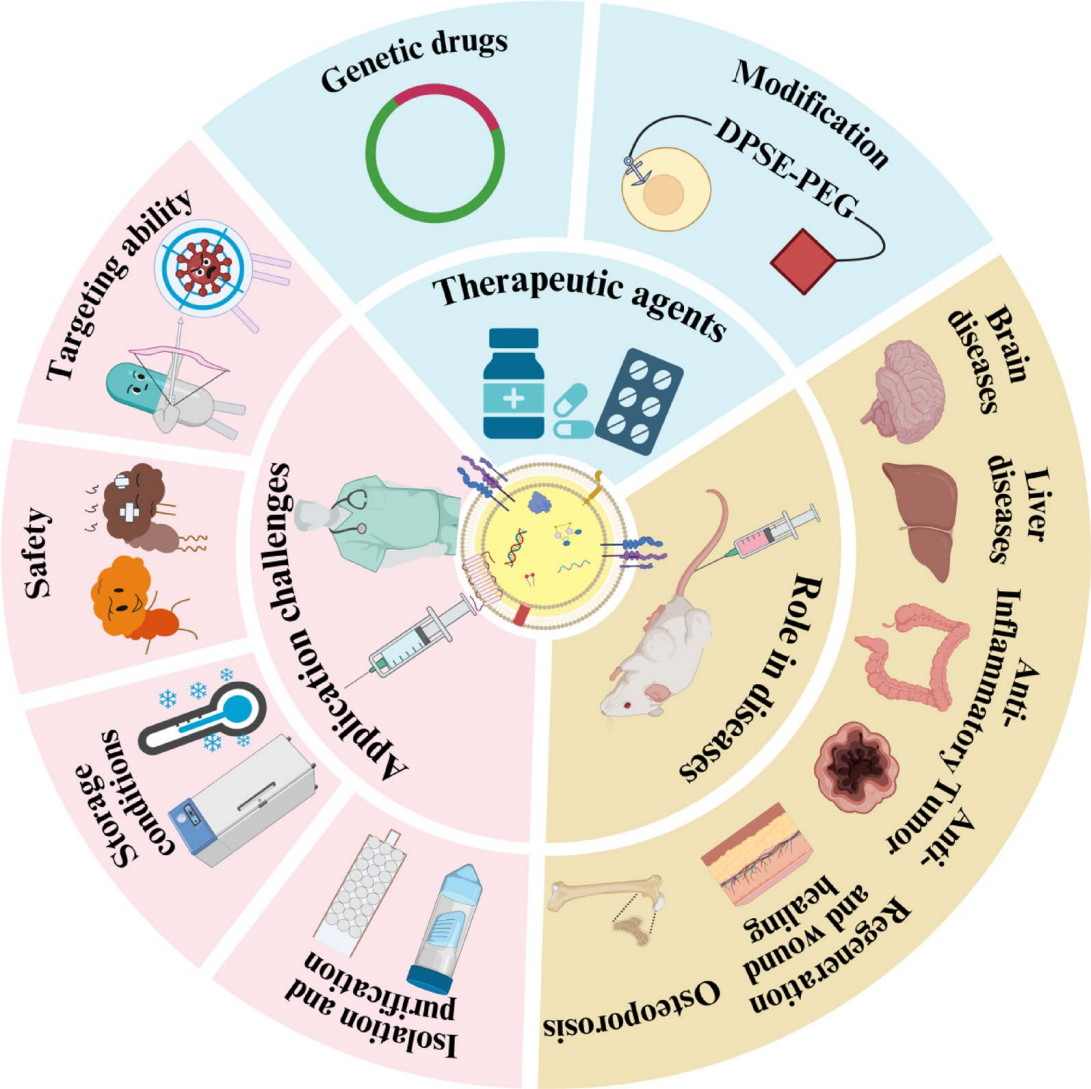

## Introduction

Extracellular vesicles (EVs) are natural lipid nanoparticles secreted by all cells [1, 2], including those of plants, animals and microorganisms [3]. They contain a diverse array of bioactive molecules, such as nucleic acids, lipids, proteins, and carbohydrates, and are integral to mediating intercellular communication [4]. Based on their biogenesis and release pathways, EVs are classified into distinct subtypes—exosomes, microvesicles and apoptotic bodies [5, 6], which are typically in the 40–1000 nm size range [7]. Because EVs originate intracellularly and share the same composition as parental cells, they play a key role in intercellular communication in organismal health and disease [8]. EVs have attracted attention as valuable diagnostic and therapeutic tools [9, 10]. Historically, research has focused on mammalian-derived vesicles (MEVs), and disappointingly, they present several problems, including complex extraction processes, limited yield, high cost, rapid blood clearance, and increased risk of cancer stimulation [11]. Therefore, in response to the above problems, it is necessary to search for safe, easy-to-access, and high-yield EVs to accelerate the translation of EVs from laboratory research to clinical applications.

Natural plant-derived vesicles have non-immunogenic and high-yield properties not found in mammalian EVs and other synthetic drug carriers [12]. Moreover, natural plant-derived vesicles possess some of the bioactive functions of the source plant, such as anti-inflammatory [13], antioxidant [14] and anticancer functions [15]. However, plant-derived vesicles were observed using transmission electron microscopy (TEM) as early as the 1960s [16]. Despite these early findings, researchers did not delve so deeply into them that they were overshadowed by the later discovery of mammalian-derived EVs. Until the early 2000s, An et al. reported that the appearance of ultrastructural secretions was clearly observed via TEM studies of fungus-invaded barley leaf cells [17, 18], Subsequently, Exosome-like vesicles were first isolated from sunflower seeds by the vacuum infiltration-centrifugation method by Regemte et al. in 2009 [19]. Since then, scholars have isolated exosome-like vesicles from different plants [20]. including broccoli [21], bitter melon [22], ginger [23], and lemon [24], etc.

The ability of plant-derived vesicles to be isolated quickly and efficiently from plant cells is one of the current challenges. Two methods, tissue infiltration centrifugation and tissue disruption, have been reported to preprocess plant tissues and have become the preferred methods for isolating vesicles from plant cells in this field [25]. After tissue pre-processing, ultracentrifugation is commonly used to isolate plant-derived vesicles [26], but the isolated nanovesicles are not pure EVs, probably because they contain cytoplasmic intracellular membrane contaminants [27], thus, finding antibodies to plant species that are universally or specifically labeled for plant-derived vesicles is necessary for the isolation of pure plant EVs. As stated above, the EVs extracted from the samples may also contain other intracellular components, and a variety of terms have been used for the prepared plant-derived vesicles in various studies, including “plant-derived extracellular vesicles”, “plant-derived nanovesicles”, “plant exosome nanovesicles”, “plant-derived exosome-like nanoparticles” and exosome-like nanoparticles”, etc [12, 26, 28, 29]., so standardized nomenclature is necessary. Since there is no consensus yet, in this review we have standardized their nomenclature as plant-derived nanovesicles (PDNVs).

In recent years, an increasing number of scientists have focused on PDNVs in the biomedical field. PDNVs contain a variety of biologically active biomolecules, can therefore be used as biotherapeutic agents or drug carriers as alternative cell-free treatments [30], and play important roles in preventing and treating a wide range of diseases [31]. As expected, PDNVs are non-toxic, low-immunogenicity, low-cost, high-yield and free of human pathogenic pathogens, offering significant advantages over mammalian exosomes and synthetic carriers, and there are no ethical concerns associated with PDNVs, all of which are attributed to their wide availability and natural sources [11, 32]. Although PDNVs present a promising platform for drug delivery, several critical challenges must be addressed to fully realize their potential as biotherapeutic agents or drug carriers. Key concerns include optimizing the purity, yield, and stability of PDNVs, as well as enhancing their capacity for precise, site-specific targeting in disease contexts—factors that are essential for their successful translation into clinical practice. This review discusses current extraction techniques, outlines the physicochemical and biochemical properties of PDNVs, and examines their internalization mechanisms, biodistribution, and biosafety profiles. It further summarizes the therapeutic applications of PDNVs across various disease models and reviews ongoing clinical trials. Finally, the review offers a perspective on future directions and the potential advancements in PDNV-based therapies.

## Biogenesis of PDNVs

**Fig. 1:**
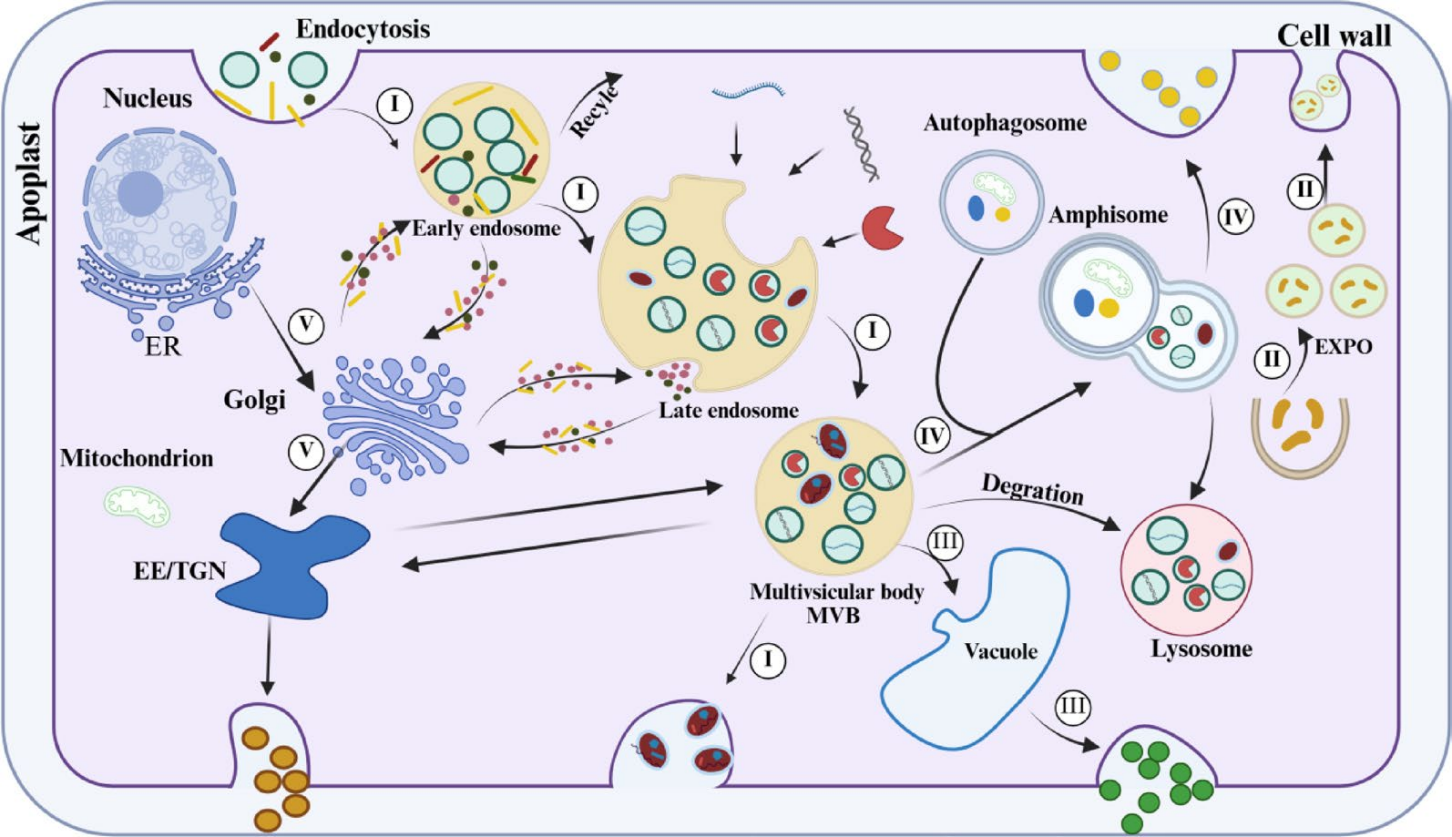
Biogenesis of PDNVs. (Ⅰ) MVB fuses with PM to release ILVs into the extracellular space; (Ⅱ) Secretion of EXPO; (Ⅲ) Vacuole fuses with PM to release ILVs; (Ⅳ) Autophagosomes fuse with MVBs to form amphitrosomes, which fuse with the plasma membrane, and (Ⅴ) TGN pathway. EE: early endosome. Created with Biorender.com

The unique biogenesis of MEVs has been widely validated, exosomes are formed from vesicles formed by inward invagination of endosomal membranes and fusion of multivesicular bodies (MVBs) with the plasma membrane, Microvesicles are formed by direct outward budding of the plasma membrane of healthy cells [33], and apoptotic bodies are formed during apoptosis by outward blebbing of the plasma membrane of the cell [34, 35]. The biogenesis of PDNVs has received less comprehensive investigation than that of MEVs, The reason that the biogenesis of PDNVs has been less comprehensively studied than that of MEVs may be due to the fact that the presence of cell walls in plant cells means that communication between them is more complex, and therefore exploring the mechanisms of biogenesis of PDNVs would be more difficult [28, 36]. And researchers have now proposed several models to explain how PDNVs are secreted (Fig. 1).

However, in the 1960s, researchers identified ultrastructural MVBs in plant cells by TEM [16], and then in 2006, An et al. observed MVBs in barley leaf cells as well [17]. Several potential mechanisms have been proposed to elucidate the secretory process of PDNVs. For example, through MVBs, exocyst-positive organelles (EXPO), autophagosomes and vacuoles pathways [37, 38]. Briefly, the biogenesis of PDNVs begins with the formation of a trans-Golgi network or early endosomes that mature into multivesicular endosomes (MVEs) or MVBs. Characteristic intraluminal vesicles (ILVs) actively and selectively integrate RNAs, including mRNAs, microRNAs (miRNAs), and other noncoding RNAs, as well as lipids and DNA, which inhabit MVBs and eventually bind to the plasma membrane to release PDNVs [39]. This process is similar to that of mammalian EVs, which are secreted via the conventional protein secretion (CPS) and unconventional protein secretion (UPS) pathways. In plant cells, soluble proteins and membrane proteins with an N-terminal signal peptide (SP) are synthesized in the endoplasmic reticulum (ER), transported to the Golgi for further modification, and subsequently transported to the trans-Golgi network (TGN), where they are sorted to the plasma membrane (PM) for secretion. On the other hand, the UPS is responsible for the secretion of proteins without SP or other cytoplasmic material into the extracellular space, which may lead to the formation of EVs [40].

EXPO and autophagosomes are both spherical double-membrane organelles, but unlike MVBs and autophagy, and despite their structural similarities [41], EXPO does not co-localize with autophagosomes and is not affected by nutrient deprivation. Therefore, these two organelles are considered as separate entities [42]. EXPO has a bilayer structure and is capable of fusing with the plasma membrane and discharging its internal vesicles into the extracellular space, Permeabilization experiments were performed for 10 min using wild Arabidopsis and tobacco BY-2 cells expressing Exo70E2-GFP to determine whether cytosolization reaches the extracellular level after the fusion of EXPO with the plasma membrane. A slightly weaker speckled fluorescent signal was observed [37, 43]. Vesicles are single-membrane organelles containing hydrolytic enzymes that are important in plant defense [44], and vesicles can release antimicrobial and cell death-inducing proteins into the extracellular compartment of *Arabidopsis* cells by fusion with the plasma membrane [45]. Moreover, the formation of intraluminal vesicles within small vacuoles (SV) predominantly results from the fusion and maturation of MVBs, while the central vacuole arises through the transformation of MVBs into SV, followed by SV fusion events [46], suggesting a potential link between the vacuole and MVBs pathways. Overall, the secretion pathway of PDNVs is essentially based on the plant infection response [47–49]. When plant cells are infected by pathogens, MVBs, vacuoles, and EXPOs fuse with the plasma membrane and the vesicles within them are released into the extracellular environment, and when these vesicles rupture, they secrete defenses to inhibit pathogen proliferation. On the other hand, autophagosomes fuse with lysis compartments such as vacuoles, bind to MVBs, and subsequently fuse with the plasma membrane, eventually releasing vesicles [45, 50].

## Preparations of PDNVs

PDNVs can be isolated in high yields from plant juices, directly from plant organs (e.g. fruits, roots and leaves), and even from whole plants [51]. Extraction methods for PDNVs are similar to those for MEVs, and the correct extraction and purification methods are critical for analyzing EVs, as they are often present in overly complex matrices [26]. According to the guidelines of the International Society for Extracellular Vesicles (ISEV)-2018, the required purity and specificity of EVs preparations should be determined by the specific experimental objectives and the intended application of the EVs [52]. Scientists are also currently trying to improve the extraction specificity, efficiency and recovery of PDNVs by combining multiple methods, which remains a challenge that needs to be urgently overcome. In this section, we first introduce the method of pre- processing the tissues before extracting PDNVs, and subsequently, after pre-processing, we briefly introduce the preparation method of enriched PDNVs.

### Tissue pre-processing methods

#### Tissue-disruption method

First, before separation, we should try to keep the batch and condition of the raw materials consistent. Second, the selected raw materials need to be washed repeatedly. The selected fresh plants are subsequently dissociated in a blender to obtain the plant juice [53]. In this process, different parts of the plant need to be treated, for example, for some plants with juicy fruits, such as tomatoes [54], grapefruit [55] and lemon [24], after washing can be directly extracted juice [56], whereas for some hard plant rhizomes, such as ginseng [57], ginger [58, 59], etc., it is necessary to add some phosphate buffer solution (PBS) to promote the production of sample juice in the juicer. This process may influence the composition and functional integrity of the final product (e.g. damaging the cells by mixing their organelles or the membrane structure of the plasma membrane with the EVs), and the speed, intensity, and duration of juicing in this step need to be carefully selected according to the raw material.

#### Tissue-infiltration centrifugation method

The plant apoplast is a distinct extracellular compartment situated outside the plasma membrane, serving as a site for various essential biological processes. The fluid extracted through infiltration-centrifugation is referred to as apoplast washing fluid (AWF) [60]. Studies have shown that variations in the osmotic pressure of the infiltration medium and the duration between the onset of infiltration and the completion of centrifugation exert minimal influence on AWF composition [61]. In 2009, Regente et al. were the first to report the presence of exosome-like vesicles with a distinct membrane structures in the sunflower apoplast [19]. A number of studies have reported the isolation of PDNVs from AWF in plant tissues, such as olive pollen tubes [62], *Arabidopsis* leaves [63] and *Nicotiana benthamiana* leaves [47]. Chen et al. conducted a comparative analysis of common isolation techniques for PDNVs derived from *Arabidopsis* and established an optimized protocol for obtaining purified AWF. The procedure involves excising the distal (blade) portion of the leaf at the base, removing the petiole, rinsing the sample with Milli-Q water to eliminate residual surface moisture, and loading the leaves into a needleless syringe. An infiltration buffer (pH 6.0) is then introduced, composed of 20 mM MES hydrate, 2 mM calcium chloride, 0.1 M sodium chloride, and Milli-Q water adjusted to 1 L and pH 6.0 using NaOH. The syringe piston is gently depressed to expel air, allowing the buffer to infiltrate the leaf tissue. After infiltration, the piston is withdrawn, the infiltrated leaves are collected, and any excess buffer on the surface is carefully removed. Transparent tape was placed with the sticky side up and one end wrapped around a 1 mL syringe, and the infiltrated leaves were placed in layers on the sticky side with the base of the leaf facing the top of the syringe. Then, the syringe was placed into a 50 mL Falcon tube, which was then centrifuged for 10 min at 900×g at 4 °C to collect the AWF. PDNVs were subsequently isolated from AWFs via a density gradient and immunocapture of *Arabidopsis* transmembrane protein 8 (TET8) [64]. Therefore, there is still no standard scheme for collecting PDNVs from AWF, and in addition to the final centrifugal speed, how the AWF is collected is also crucial.

Overall, an increasing number of researchers are now using the tissue disruption method for sample pretreatment, which may be attributed to the simpler operation compared with the tissue infiltration centrifugation method and the relatively high concentration of its subsequently extracted PDNVs. However, during the juicing process, cells may be damaged, which may result in the mixing of the membrane structures of organelles or plasma membranes with EVs, thus, the parameters during juicing need to be carefully selected. The tissue infiltration centrifugation method, is less damaging to the cells, and thus, the purity of the extracted PDNVs is greater, whereas infiltration buffer, if it is not treated cleanly during the subsequent process, affects the downstream analysis of PDNVs. Therefore, the appropriate pretreatment method should be selected according to the parts of the plant itself, and in general, for the pretreatment of plant leaves, the use of tissue infiltration centrifugation is advocated.

### Purification methods

**Fig. 2:**
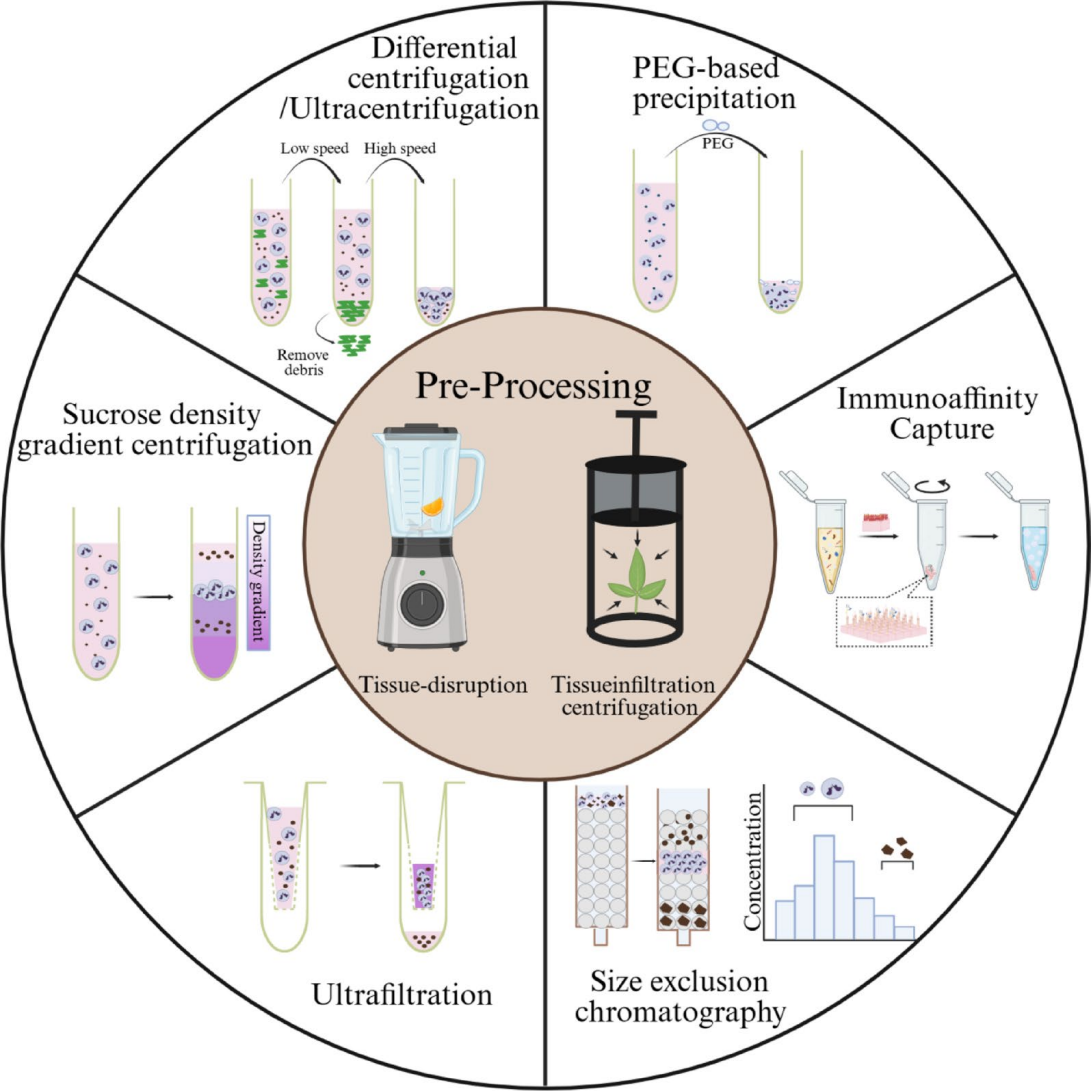
Methods for pre-processing, isolation and purification of PDNVs. Created with Biorender.com

After plant samples are pretreated, the juice or AWF obtained needs to be further purified as a way to achieve high purity, high recovery, and high throughput of PDNVs. In the subsequent sections, we detail the various techniques employed for the isolation and purification of PDNVs, as summarized in Fig. 2; Table 1.

**Table 1:** Preparation and physical characterization methods of PDNVs

Tissue-disruption	Sources	Pre-processing methods	Purification methods	Morphology check	Size (nm)	Ref.
Tissue-disruption	Grapefruit and Tomato	(1) 1500 g (30 min) (2) 3500 g (20 min) (3) 10,000 g (60 min) (4) 16,000 g (60 min) (5) 10,000 g (overnight)	UC, 150,000 g (2 h)	NTA, DLS, AFM, SEM	Grapefruit: 86–125 Tomato: 140–170	[65]
	Apple	(1) 2000 g (20 min) (2) 13,000 g (70 min)	UC, 120,000 g (130 min)	TEM	170	[65]
	Apple	(1) 2000 g (20 min) (2) 13,000 g (70 min)	UC, 120,000 g (130 min)	/	/	[66]
	Strawberry and lemon	(1) 3000 g (30 min) (2) 10,000 g (60 min) (3) 16,500 g (60 min), three times	UC, 110,000 g (60 min), two times	TEM	/	[67]
	Grapefruits	(1) 500 g (10 min) (2) 2000 g (20 min) (3) 15,000 g (30 min)	UC, 110,000 g (90 min)	TEM, NTA	147 ± 11	[68]
	Citrus species	(1) 400 g (20 min) (2) 1000 g (20 min) (3) 15,000 g (20 min)	UC, 150,000 g (60 min)	/	/	[69]
	Grapefruit	(1) 500 g (10 min) (2) 3000 g (30 min) (3) 10,000 g (60 min)	UC, 100,000 g (2 h)	DLS, NTA, Cryo-EM	170.9 ± 5.7	[70]
	Grapefruit	(1) 1200 g (20 min) (2) 3000 g (20 min), three times (3) 10,000 g (60 min) (4) 15,000 g (60 min)	UC, (1) 150,000 g (2 h) (2) 150,000 g (2 h)	NTA, DLS, AFM, Cryo-EM	30	[71]
	Bitter melon	(1) 1000 g (10 min) (2) 3000 g (20 min) (3) 10,000 g (40 min)	DGC, (1) 150,000 g (90 min) (2) gradient sucrose solution (8%, 30%, 45%, and 60%), 150,000 g (90 min)	TEM, NTA	120	[22]
	Ginger	(1) 3000 g (20 min) (2) 10,000 g (40 min)	DGC, (1) 150,000 g (2 h) (2) gradient sucrose solution (8%, 30%, 45% and 60%), 150,000 g (2 h)	TEM, AFM, DLS	292.5 (band 1), 231.6 (band 2), 219.6 (band 3)	[72]
	Ginger	(1) 3000 g (20 min) (2) 10,000 g (40 min)	DGC, (1) 150,000 g (2 h) (2) gradient sucrose solution (8%, 30%, 45% and 60%), 150,000 g (2 h)	DLS, TEM	146 ± 1.8	[73]
	Ginger	(1) 10,000 g (60 min), repeated 2 ~ 3 times	DGC, (1) 100,000 g (80 min) (2) 60% iodixanol cushion, 100,000 g (80 min) (3) Iodixanol gradient (10%,40%), 40,000 rpm (24 h)	NTA, DLS	110.5 ± 27.49	[74]
	Ginger	(1) 1000 g (10 min) (2) 3000 g (20 min) (3) 10,000 g (40 min)	DGC, (1) 150,000 g (90 min) (2) gradient sucrose solution (8%, 30%, 45% and 60%), 150,000 g (2 h)	DLS, AFM	8/30% layer: 386.6, 30/45% layer: 294.1	[75]
	Ginseng	(1) 200 g (10 min) (2) 2000 g (20 min) (3) 10,000 g (30 min)	DGC, (1) 100,000 g (60 min) (2) gradient sucrose solution (15%, 30%, 45% and 60%), 150,000 g (60 min) (3) 100,000 g (60 min)	TEM, DLS	344.8	[76]
	Ginseng	(1) 2000 g (20 min) (2) 10,000 g (60 min)	DGC, (1) sucrose cushion solutions (27%, 68%), 100,000 g (90 min) (2) gradient sucrose solution (8%, 30%, 45% and 60%), 200,000 g (90 min)	NTA, TEM, DLS	105.8 ± 47.85	[77]
	Ginseng	(1) 2000 g (20 min) (2) 10,000 g (60 min)	DGC, (1) sucrose cushion solutions (27%, 68%), 100,000 g (90 min) (2) gradient sucrose solution (8%, 30%, 45% and 60%), 200,000 g (90 min)	DLS, NTA, TEM	71.42	[57]
	Ginseng	(1) 3000 g (30 min) (2) 10,000 g (60 min)	DGC, (1) 150,000 g (2 h) (2) gradient sucrose solution (8%, 30%, 45% and 60%), 150,000 g (2 h)	LDS, TEM	8/30% layer: 241.1 ± 3.8 30/45% layer: 144.1 ± 2.8 60% layer: 340.1 ± 15.9	[78]
	Ginseng	(1) 200 g (10 min) (2) 2000 g (20 min) (3) 10,000 g (30 min)	DGC, (1) 100,000 g (60 min) (2) gradient sucrose solution (8%, 30%, 45% and 60%), 150,000 g (60 min) (3) 100,000 g (60 min)	DLS, TEM	45% layer: 344.8	[76]
	Grapefruit	(1) 500 g (10 min) (2) 2000 g (20 min) (3) 5000 g (30 min) (4) 10,000 g (60 min)	DGC, (1) 100,000 g (2 h) (2) gradient sucrose solution (8%, 30%, 45% and 60%) in 20 mM Tris. HCl, pH 7.2, 150,000 g (2 h)	TEM, DLS	113.4 ± 8.9	[79]
	Grapefruit	(1) 500 g (10 min) (2) 2000 g (20 min) (3) 5000 g (30 min) (4) 10,000 g (60 min)	DGC, (1) 100,000 g (2 h) (2) gradient sucrose solution (8%, 30%, 45% and 60%) in 20 mM Tris. HCl, pH 7.2	DLS, TEM	253.7	[80]
	Garlic	(1) 1000 g (10 min) (2) 3000 g (15 min) (3) 10,000 g (60 min)	DGC, (1) 150,000 g (2 h) (2) gradient sucrose solution (8%, 30%, 45% and 60%), 150,000 g (2 h)	DLS, TEM	43.82–396.1	[81]
	Garlic	(1) 1000 g (10 min) (2) 5000 g (20 min) (3) 10,000 g (30 min)	DGC, (1) 150,000 g (90 min) (2) gradient sucrose solution (8%, 30%, 45% and 60%), 100,000 g (90 min)	TEM, DLS	30/45% layer: 182.2	[82]
	Blueberry	(1) 1000 g (10 min) (2) 5000 g (20 min) (3) 11,000 g (50 min)	UF + UC, (1) Ultrafiltration (10,000 MWCO) (2) 120,000 g (90 min)	DLS, SEM	198 ± 112	[83]
	Pomegranate fruits	(1) 700 g (10 min) (2) 3000 g (15 min) (3) 20,000 g (20 min)	SEC, 1 mL of concentrated extract was loaded into the column for elution.	TEM, NTA	148.7	[84]
	Ginger rhizomes	(1) 2000 g (10 min) (2) 6000 g (20 min) (3) 10,000 g (40 min)	PEG-6000, (1) PEG-6000 (8%/10%/12%/15%), 4℃, overnight (2) 8000 g (30 min)	DLS	8%: 365 10%: 304 12%: 263 15%: 252	[85]
	Ginger rhizomes	(1) 2000 g (10 min) (2) 6000 g (20 min) (3) 10,000 g (40 min)	PEG-6000, (1) PEG-6000 (8%), pH = 4/5, overnight (2) 8000 g (30 min)		pH = 4: 294.1 pH = 5: 343.8	[86]
	Grapefruit	(1) 1000 g (10 min) (2) 10,000 g (10 min)	PEG/ dextran (DEX), (1) Filtration (0.45, 0.22 μm, ) (2) 2×PEG/DEX, 1000 g (10 min), two times (3) 1000 g (10 min), two times	NTA, AFM	132	[55]
	Lemon	(1) 3000 g (10 min) (2) 10,000 g (20 min)	ELD, 300 kDa cut-off dialysis bag placed in a gel holder cassette and a current of 300 mA was used to isolate PDEV	TEM, NTA	< 200	[87]
	Bitter melon	(1) 3000 g (10 min) (2) 10,000 g (20 min)	ELD, (1) Filtration (0.22 μm ) (2) 300 kDa cut-off dialysis bag placed in a gel holder cassette and a current of 300 mA was used to isolate PDEV	TEM, NTA	/	[88]
	Fruit and vegetable	Filtration	(1) Plant supernatants were mixed with ammonium sulfate and deposited in a cuvette of C-CP tips, 300 g (1 min) (2) Matrix proteins were eluted with 25% glycerol and 1 M ammonium sulfate PBS solution, 300 g (1 min) (3) 50% glycerol PBS solution elution, 300 g (1 min)	TEM	/	[89]
	Onion	(1) 20,000 g (60 min) (2) 8000 g (60 min)	UF + SEC, 5000 g	NTA, TEM, DLS	150	[90]
	Wheat	(1) 300 g (10 min) (2) 16,000 g (30 min)	Exo-spin™+ Filtration (0.22 μm )	SEM	40–100	[91]
Tissue-Infiltration	Bblueberry	(1) 2000 g (20 min) (2) 10,000 g (30 min)	UC, (1) 40,000 g (6 h) (2) 100,000 g (6 h)	NTA, TEM	95.7 ± 3.1	[92]
	Sunflower seeds	(1) 500 g (15 min) (2) 10,000 g (30 min)	UC, 100,000 g (60 min)	TEM	30–150	[48]
	*Nicotiana benthamiana* leaves	(1) 700 g (20 min) (2) 10,000 g (30 min) (3) 10,000 g (30 min)	UC, 100,000 g (3 h)	TEM	117 ± 9	[93]
	*Arabidopsis and Brassica* plants	(1) 700 g (10 min) (2) 2000 g (20 min) (3) Filter through 0.22 μm (4) 10,000 g (30 min)	DGC, (1) 100,000 g (3 h) gradient sucrose solution (2 M, 1.3 M, 1.16 M, 0.8 M, 0.5 M, and 0.25 M), 100 000 g (17 h)(2)	TEM, Cryo-EM, NTA	60–200	[94]
	*Arabidopsis*	(1) 700 g (20 min) (2) 10,000 g (30 min)	DGC, (1) 40,000 g (60 min) to obtain P40 fraction (2) 100,000 g (60 min) to obtain P100 fraction (3) Iodixanol gradient (5, 10, 20, and 40%), 100 000 g (17 h) (4) 100,000 g (60 min)	DLS,	P40 fraction: 50–300 P100 fraction: 10–17	[63]
	*Arabidopsis*	(1) 900 g (10 min) (2) 2000 g (10 min) (3) 10,000 g (30 min)	IC, (1) 100 000 g for 1 h in twice times to obtain P100 fraction (2) Incubate P100 fraction with TET8-specific antibody-linked beads	/	/	[27]
	*Nicotiana tabacum* L.	(1) 400 g (20 min), two times (2) 15,000 g (20 min), two times (3) 50,000 g (90 min)	Agarose gel Electrophoresis, 20,000 g (20 min)	Cryo-EM, DLS	/	[95]

#### Ultracentrifugation

Ultracentrifugation (UC) is the most widely employed method for isolating EVs [96, 97], The separation principle of UC is that particles of different sizes have different settling rates under centrifugal force [98]. In the context of PDNV isolation, plant homogenates are initially subjected to low-speed centrifugation (500–3000×g) to remove large particulates such as plant fibers. This is followed by medium-speed centrifugation (2000–10,000×g) to eliminate cellular debris, and finally by high-speed centrifugation (100,000–150,000×g) to collect PDNVs [28]. UC offers advantages including procedural simplicity, cost-effectiveness, and scalability. However, significant variability in plant tissue types, compositions, and structural characteristics can lead to marked differences in the resulting PDNVs in terms of particle size, polydispersity index (PDI), concentration, and purity. Therefore, the specific parameters of centrifugation force and time should be selected according to different plants, and there is no standardized centrifugation method [99]. Kilasoniya et al. obtained PDNVs by differential ultracentrifugation after juicing grapefruit and tomato, which were similar in size and morphology to MEVs, the yield of grapefruit-derived PDNVs was greater than that of tomato-derived PDNVs, and the particle size was smaller [100]. Some related studies have also reported the use of UC to extract PDNVs from apples [65, 66]. As the UC process involves a long term and high speed centrifugation, which may lead to the disruption of the morphology and structure of PDNVs, combined with the experience of MEVs extraction, a thin layer of sucrose or 60% iodixanol can be placed at the bottom of the centrifuge tube as an isotonic cushion [101].

#### Density gradient centrifugation

Density gradient centrifugation (DGC) is an improved separation technique based on UC, in which EVs are separated from other particles due to different densities as each particle moves to the equilibrium of centrifugal force and buoyancy in a density gradient solution in which a sucrose solution with a density gradient is widely used for purification of PDNVs [102, 103]. The procedure was as follows: after adding the sample was separated to the bottom of the centrifuge tube, a gradient solution of sucrose was subsequently added from low to high concentrations (8%, 30%, 45%, and 60%), and after the preparation was completed, ultracentrifugation is carried out to collect the sucrose bands in the 30%/45% layer, which is how to obtain the purified PDNVs [26]. Wang et al. After juicing bitter melon, the juice was centrifuged, the supernatant was collected, transferred to a sucrose gradient solution, and the bands at the sucrose layer (30%-45%) were collected and washed with PBS to obtain PDNVs [22]. Similarly, Zhang et al. utilized DGC to isolate and purify nanovesicles from ginger. Characterization of vesicles across different sucrose gradient layers revealed that the average particle size was 292.2 nm in the 8/30% interface (band 1), 231.6 nm in the 30/45% interface (band 2), and 219.6 nm in the 45/60% interface (band 3). The nanovesicles in band 1 and band 2 were resistant to freeze-thaw cycles and stable at room temperature for up to 7 days, whereas the nanovesicles in band 3 presented low and unstable yields [72]. There are also many related reports on the use of DGC to extract PDNVs from ginger [73, 75]. Liu et al. pre-treated Arabidopsis and Brassica plants by tissue infiltration centrifugation followed by differential centrifugation to obtain larger vesicles, which were ultimately purified by ultracentrifugation using a six-layer sucrose concentration gradient [94]. In addition to the sucrose gradient substrate that can be used, iodixanol is often used [104]. Rutter et al. prepared discontinuous iodixanol gradients (5%, 10%, 20%, 40%) to purify vesicles from Arabidopsis sources [63].

#### Ultrafiltration

Ultrafiltration (UF), similar to conventional filtration techniques, employs nanoporous membranes through which smaller molecular weight components pass under applied fluid pressure, while larger molecules are retained by the membrane barrier [105]. Lee et al. isolated PDNVs from fresh leaves and stems of Dendropanax morbifera by grinding them with an extractor and then filtering the resulting juice, followed by centrifugation at 10,000 × g for 10 min and filtration of the supernatant with a 0.22 μm filter membrane, and then concentrating the samples using a 100 K filter to obtain PDNVs [106]. Similarly, Robertis et al. processed blueberry juice by performing low-speed centrifugation, followed by sequential filtration through a 0.45 μm membrane and ultrafiltration using a 10,000 MWCO membrane. The concentrated filtrate was then passed through a 0.22 μm filter and subjected to ultracentrifugation to isolate PDNVs [83]. While this method is relatively straightforward and time-efficient, it is best suited for dilute juice samples, as the filter membranes are prone to clogging. This can result in reduced vesicle recovery, lower purity, and decreased reusability of the filtration apparatus.

#### Size exclusive chromatography

Size exclusion chromatography (SEC) is a technique for separating molecules or particles based on their hydrodynamic volume [107]. That is, components with larger hydrodynamic radii are not able to enter the pore space, leading to early elution, in contrast, components with smaller hydrodynamic radii can enter the pore space, leading to late elution [26]. Christian et al. used SEC for the first time to isolate PDNVs from pomegranate juice and obtained highly concentrated PDNVs without most non-PDNVs co-isolated proteins, which were characterized by nanoparticle tracking analysis (NTA) and transmission electron microscopy (TEM) to determine their concentration/volume, size and morphology [84]. Recently, however, new studies have shown that, compared with UC, extraction using SEC can produce EVs with more complete biophysical properties, but whether this leads to functional differences remains to be investigated [108]. YYou et al. isolated PDNVs from cabbage using SEC, PEG, and UC techniques and reported that PDNVs obtained using the SEC technique presented the smallest particle size and the highest yield and purity; therefore, the SEC-based isolation method can be used as a standard method for isolating PDNVs, which is not limited to the isolation of PDNVs from cabbage but can also be used for other types of plants [109].

#### Immunoaffinity capture

Due to the presence of a large number of proteins and receptors on the membranes of EVs, the technique that utilizes the immunoaffinity interactions between these proteins (antigens) and antibodies, as well as the specific interactions between receptors and ligands, to isolate EVs is called immunoaffinity capture (IC) [110]. This technique has been widely used for the isolation of mammalian EVs such as common markers of mammalian EVs (e.g., CD9, CD63, CD81), and this method is highly sensitive and has high sample purity [111]. However, the lack of specific antibodies for recognizing different plants and the high cost of the method may limit its usefulness, making it extremely difficult to use IC to extract PDNVs in the plant kingdom. Tsai et al. identified the gene for TETRASPANIN (TET) in Arabidopsis thaliana, in which TET8 and TET9 are structurally similar to mammalian CD63, and observed that TET8-associated vesicles may be derived from MVB [112]. He et al. employed magnetic beads coated with anti-TET8 antibodies to specifically recognize the EC2 exocystic ring domain of TET8, enabling the targeted capture of TET8-positive PDNVs following UC [113]. In addition, Wen et al. developed a novel platform designed to remove contaminants and improve the purification of PDNVs. In this work, they present a novel integrated platform for the efficient separation and purification of PDNVs by utilizing the unique property of magnetic beads conjugated to the Concanavalin A (Con A) lectin and validated the utility of the platform by using licorice-sourced PDNVs, which achieved accurate PDNV enrichment while dramatically reducing the separation time [114].

#### Other purification methods

In addition to the aforementioned purification techniques, several alternative methods have been developed for the isolation of PDNVs. One such approach, previously reported for the extraction of extracellular vesicles from mammalian cells, involves polyethylene glycol (PEG) precipitation. In this method, a polymer solution is employed to reduce the solubility of EVs, facilitating their precipitation, which can then be collected through low-speed centrifugation [115, 116]. Kalarikkal et al. developed a PEG-6000-based method to purify PDNVs from ginger rhizome sources and were able to recover 60–90% of the PDNVs to a greater extent than UC by using different concentrations of PEG-6000 (8%, 10%, 12%, and 15%) and compared with PDNVs extracted by UC, the size and zeta potential of PDNVs were almost the same, and the protein, lipid, small RNA and bioactive contents were comparable [85]. In a follow-up study, Suresh et al. reported that the recovery of PDNVs using PEG-6000 (10%) at different pH values (pH = 4/5/6/7/8/9) increased 4-5-fold when PEG precipitation was carried out at low pH conditions (pH = 4/5), resulting in smaller sizes and no significant change in the zeta potential [86]. Savcı et al. also used PEG/dextran (DEX) precipitation to isolate PDNVs from grapefruit [55].

In addition to the method by PEG precipitation, Yang et al. combined electrophoresis with a 300 kDa cutoff dialysis bag (ELD) for the isolation of PDNVs from lemon juice and reported similar sizes and numbers of vesicles with structural integrity to those of UC, which is both time-saving and does not require special equipment compared with other isolation methods [87]. PDNVs were also obtained from bitter melon by the ELD method of by Yang et al. [88]. Additionally, Jackson et al. developed a rapid isolation method for PDNVs with an average diameter of 189 nm from 20 commonly consumed fruits and vegetables using hydrophobic interaction chromatography (HIC) facilitated by capillary channel polymer (C-CP) fiber spin tips [89]. Agarose gel electrophoresis has also been reported as an effective technique for removing soluble contaminants from PDNV preparations. In this method, pretreated PDNVs are loaded onto an agarose gel and subjected to electrophoresis, after which the electrophoresed gel is centrifuged at 20,000 ×g for 20 min to recover high-purity PDNVs [95]. Zhao et al. enzymatically degraded the cell walls in the roots of Morinda officinalis to release PDNVs and compared the results with those of the extraction of PDNVs by milling and reported a greater yield and smaller size of PDNVs by enzymatic degradation [117].

#### Combined isolation method

Since plant extracts contain large molecules such as cellulose and starch, it becomes more difficult for extracting PDNVs with higher purity [28]. As mentioned above, any single method has some drawbacks, so some researchers have now developed combinatorial isolation methods to improve yield and specificity. For example, Kang et al. obtained PDNVs by centrifuging the juice obtained from juicing onions to remove large fragments and then filtering it using UF centrifugation, followed by concentrating the supernatant using SEC, which showed that most of the protein impurities were separated after Sect [90]. Similarly, Fikrettin et al. employed a combined approach using the Exo-spin™ kit and 0.22 μm pore-size filters to isolate PDNVs from wheatgrass wort. The resulting vesicles, ranging in size from 40 to 100 nm, expressed typical exosome markers such as HSP70, CD9, and CD63 [91].

Overall, for each step from pretreatment to final purification to obtain PDNVs, each step is critical. For example, different pretreatment methods can be selected according to different parts of a plant. For plant leaves, the tissue infiltration centrifugation method is usually used, which is suitable for the separation of high-purity EVs, but whether the vesicle isolation buffer (VIB) used in this process affects the analysis of downstream metabolites should be a more worthwhile consideration. Plant parts such as fruits and rhizomes are usually pretreated by tissue disruption but are more likely to produce high yields of mixed EVs. Among the subsequent multiple purification methods, UC, which is the most commonly used method, is widely favored by researchers because of its simplicity of operation and is usually used in combination with DGC as a way of obtaining higher-purity PDNVs; other combinations have also been reported in current studies. However, each purification method presents its own set of advantages and limitations (Table 2). Therefore, the selection of appropriate pretreatment, isolation, and purification techniques should be carefully tailored to various factors, including the specific plant species, laboratory conditions, research objectives, and experimental goals, in order to achieve optimal outcomes.

**Table 2:** The advantages and disadvantages of PDNVs isolation methods

Methods	Advantages	Disadvantages	Ref.
Ultracentrifugation	Current gold standard, Simple operation, Large sample capacity, High recovery rate.	Time-consuming, Easy to aggregate pellet, Low purity, High equipment requirements.	[29, 43]
Density gradient centrifugation	Current gold standard, High recovery, and purity.	Time-consuming, Large centrifuge equipment, Contamination of density gradient medium, Low yield.	[25, 118]
Ultrafiltration	Simple, Low cost, Rapid.	Easy to cause clogged pores; EVs are adsorbed on the filter surface; Leading to a loss of yield; Shear forces may damage EVs.	[7, 119]
Size Exclusive Chromatography	Fast, Simple, and low cost, Complete structure, and uniform size.	High cost, Difficulty in large-scale production, Specialized equipment.	[120]
Immunoaffinity Capture	Strong specificity, High purity.	High cost, Lack of universal PDVLNs markers, Dearth of commercial antibodies, Not suitable for large samples, May lose original functional activity.	[25, 121, 122]

## Characterization of PDNVs

In this section, we elucidate the physical and biochemical characterization, stability and storage of PDNVs. Like for mammalian EVs, physical characterization typically involves measuring the particle size distribution, particle number, morphology, and surface charge to determine the physical characteristics of PDNVs [25]. Depending on the type and origin of the plant or fruit, PDNVs carry distinct molecular cargos—such as lipids, nucleic acids, proteins, and metabolites—that define their biochemical characteristics and inform the development of therapeutic agents targeting these biomolecules in human diseases [123]. The stability and optimal storage conditions of PDNVs following extraction and purification warrant thorough investigation, as these factors are critical for maintaining their functional efficacy in preclinical models and determining their potential for clinical translation.

### Physical characteristics

The physical characteristics of the PDNVs were evaluated in terms of their particle size distribution, zeta potential, and morphology. Commonly used methods for determining the particle size of PDNVs include dynamic light scattering (DLS) [124], nanoparticle tracking analysis (NTA) [125], and laser transmission spectroscopy (LTS) [83]. DLS has become a benchmark technique for evaluating the particle size distribution of nanoparticles in a number of scientific fields because of its ability to calculate accurate particle sizes with only a small sample size [39]. However, a non-negligible disadvantage of DLS is its low resolution [126]. NTA is a high-throughput, single-particle method for quantitatively assessing particle diameter and concentration, and has become the gold standard for the detection of PDNVs because of its fast detection rate. It has good reproducibility and resolution and is not easily affected by the high scattering intensity of large particles [127, 128]. The particle size and potential of PDNVs from different plant sources vary considerably, with average particle sizes ranging from 30 nm to 400 nm and potentials ranging from nearly neutral to about − 70 mV [106, 129, 130]. This may be related to the fact that PDNVs from different species sources contain different compositions or have different biogenesis processes, and differences in isolation and purification methods may also have an effect on particle size and potential [94]. Electron microscopy (EM) is one of the most commonly used techniques for high-resolution imaging of PDNVs. Transmission electron microscopy (TEM) is commonly used to observe the ultrastructures of PDNVs in their subcellular state, with the majority of PDNVs presenting a cup-shaped structure [131, 132] and certain plant-derived PDVLNs presenting a nearly spherical morphology [69, 133]. Owing to the different kinds of EM for detecting PDNVs, the preparation of the sample is performed differently, e.g., the commonly used EM techniques, scanning electron microscopy (SEM) and TEM, require fixation, dehydration, and staining of the samples, which may lead to vesicles being dehydrated and deformed, and their morphology to becoming a cup-shaped type. In contrast, Cryo-electron microscopy (Cryo-EM) analyzes samples at very low temperatures (e.g., -100 °C), avoiding the effects of dehydration and chemical fixatives, and its vesicle morphology takes on a spherical shape. Atomic force microscopy (AFM) is a widely utilized high-resolution imaging technique for the characterization of vesicles [98, 134, 135]. Its exceptional resolution enables the detailed visualization of three-dimensional morphology, as well as the assessment of physical properties such as viscoelasticity and membrane stiffness [136]. For instance, Garaeva et al. used NTA, DLS, AFM and Cryo-EM techniques to visualize the size, number and morphology of PDNVs extracted from grapefruit and observed in Cryo-EM that the average size of the PDNVs was 41 ± 13 nm, their morphology was spherical, and the thickness of their lipid bilayer was 5.3 ± 0.8 nm [71].

### Biochemical content

In general, the composition of PDNVs from different plant species varies, and consists mainly of lipids, proteins, nucleic acids, and other metabolites, depending on their parent source [137]. These components play various roles in physiological and pathological processes [138]. Subjecting PDNVs to compositional analysis is an important characterization criterion for quality control of PDNVs [139]. The evaluation of intracellular proteins, RNA, lipids, and secondary metabolites in PDNVs has been reported using various analytical techniques, including polyacrylamide gel electrophoresis (PAGE), agarose gel electrophoresis, thin-layer chromatography (TLC), and high-performance liquid chromatography (HPLC) [140, 141]. Accordingly, this section provides a detailed overview of the molecular components of PDNVs, as summarized in Table 3.

#### Lipids

An important component of the vesicle bilayer structure is lipids [142], which are also involved in the formation and release of PDNVs [143]. The lipids found in EVs of mammalian origin are mainly phosphatidylcholine (PC) and cholesterol, whereas no cholesterol [9], which consists mainly of phosphatidic acid (PA), phosphatidylethanolamine (PE), phosphatidylcholine (PC), phosphatidylinositol (PI), and diacylglycerol (DG), is found in PDNVs [144, 145]. On the basis of the total lipid analysis of PDNVs, lipids can be divided into two categories: glycerolipids and phospholipids [26]. Among them, PA regulates membrane cleavage and fusion and is a major lipid mediator. The lipid composition of PDNVs also influences their cellular uptake by modulating cytoskeletal rearrangement and protein regulation during the endocytic process [146]. Bokka et al. analyzed the lipid profile of tomato-derived PDNVs using TLC, identifying the presence of PS, PG, PA, PC, and PE. Quantitatively, the vesicles contained 33% PA, 18% PG, 16% each of PC and PS, and 17% PE [147]. Furthermore, certain studies have suggested that specific lipid components in PDNVs may contribute to their antimicrobial properties. For example, tomato-derived PDNVs were shown to reverse microbiota dysbiosis caused by Clostridium polymorpha in a gut microbiota fermentation model simulator [148]. In addition, ginger-derived PDNVs prevent high-fat diet-induced obesity and insulin resistance, confirming that PA in PDNVs induces intestinal epithelial cells to express Foxa2 [149]. While lipid analysis of PDNVs from the aloe vera source revealed enrichment of various phospholipids, glucosylceramide (GlcCer) was the major lipid, and other lipids, including ceramide (Cer), PA, and PC, were identified in a report by Zeng et al. Previous studies have shown that GlcCer, which is usually considered a minor constituent, constitutes less than 5% of the total lipid extracts from plant tissues but that plasma and vesicular membranes account for about 6–30% of the total membrane lipids [150]. Liu et al. performed lipidomic analysis of PDNVs from Arabidopsis rosette leaves and reported that sphingolipids were more abundant and consisted of almost pure glycosyl inositol phosphoryl ceramide (GIPC) [151]. Researchers have also extracted total lipids from PDNVs and made nanoparticles for use as drug delivery platforms [80, 152]. For instance, Zhang et al. employed the Bligh and Dyer method to extract total lipids from ginger-derived PDNVs and incorporated siRNA-CD98 into a lipid/HEPES buffer suspension. This mixture was then processed through a 200 nm liposome extruder, resulting in uniformly sized siRNA-CD98/ginger-derived lipid carriers, which were successfully delivered to colon tissues in a murine model of ulcerative colitis [153, 154].

#### Proteins

Overall, PDNVs typically exhibit lower protein concentrations compared to proteins from mammalian-derived EVs, consisting mainly of subclasses of peripheral membrane proteins, transmembrane proteins, and intracellular proteins. However, markers for PDNVs have not been clearly reported and require deeper investigation [39, 155, 156]. Raimondo et al. reported that 56.7% of proteins from lemon-derived PDNVs overlap with EVs from mammalian-derived tissues and cells [157]. Some reports reveal different proteomics data from PDNVs samples. Liu et al. performed proteomic analysis of proteins in Leek-derived PDNVs using the reference proteomic database *Viridiplantae* and identified a variety of proteins, among which a series of plasma membrane proteins were identified, such as plasma membrane ATPases, members of the G-family of the ABC transporter, pleiotropic drug resistance proteins, and hypersensitive response-inducing proteins. Clathrin heavy and light chains, along with Ras-associated proteins—membrane-binding proteins typically associated with the plasma membrane have been identified in GC-VLNs [158]. Using mass spectrometry, Cao et al. characterized the proteome of ginseng-derived PDNVs, identifying 3,129 distinct proteins. These proteins were subsequently classified into categories based on gene ontology (GO), encompassing biological processes, cellular localization, and molecular functions [76]. In a separate study, Zhang et al. utilized DGC to purify PDNVs from ginger and analyzed the protein composition across various gradient layers using proteomic techniques. Their findings indicated a relatively low protein content, consisting mainly of cytoplasmic proteins such as actin and proteolytic enzymes, along with a smaller subset of membrane-associated proteins, including transport and channel proteins like aquaporins and chloride channels [72].

Liu et al. compared Arabidopsis leaf-derived PDNVs with previous proteomic studies of other plant-derived PDNVs and reported that their complexity was lower than that of Arabidopsis, with 1,438 of the proteins found in Arabidopsis and lower than that of Arabidopsis in grapefruit, grapefruit, and citrus juices, and that vesicle protein complexity may be the result of variability in the cellular and tissue origins of vesicles [94, 129, 130, 159]. The protein species in PDNVs from extracellular fluid and whole leaf sources were compared, and only 787 species were identified in their extracellular fluid, thus, fewer proteins in PDNVs were extracted exclusively from extracellular fluid, and the protein diversity was greater in PDNVs prepared via tissue fragmentation pretreatment [94].

Stress-responsive proteins enable plants to withstand adverse environmental conditions. EVs derived from rice have been reported to carry heat shock proteins and chaperonins, which play a crucial role in mitigating heat stress by preserving protein stability and facilitating proper folding under stress conditions [160]. Heat shock protein 70, glutathione S-transferase, adenosylhomocysteinase, glyceraldehyde-3 phosphate dehydrogenase (GAPDH), the annexin family, and adenosylhomocysteinase are among the proteins of Aloe-derived PDNVs [150]. Because relatively few studies have focused on the labeling of PDNVs, peneration1 (PEN1) was found in Arabidopsis-derived PDNVs and enriched in the lumen of PDNVs in a previous study [63], whereas in another study, TET8 was found to be expressed on the membrane surface of Arabidopsis-derived PDNVs [112], Huang et al. successfully isolated TET8-positive vesicles from the apoplastic fluid of Arabidopsis using an immunoaffinity capture assay [27]. However, whether PEN1 or TET8 can serve as universal protein biomarkers for PDNVs across diverse plant species remains an open question, requiring further investigation. Consequently, comprehensive proteomic analysis of PDNVs is essential for the identification of reliable markers.

#### RNAs

PDNVs are known to encapsulate various types of nucleic acids, including messenger RNAs (mRNAs), microRNAs (miRNAs), and small interfering RNAs (siRNAs), which may facilitate both intercellular and interspecies communication [11, 112, 161]. Teng et al. administered ginger-derived PDNVs to mice with colitis, and owing to the miRNAs in the PDNVs, they were found to be absorbed by the gut microbiota, where they mediate communication between the gut microbiota and the host immune system and thus alleviate the disease [145]. Xiao et al. isolated PDNVs from 11 different edible fruits and vegetables and identified a total of 418 miRNAs within these samples. Subsequent target prediction and functional enrichment analyses revealed that the most abundantly expressed miRNAs were strongly linked to inflammatory responses and cancer-related signaling pathways [56]. Teng et al. used miRNAs from ginger-derived PDNVs to inhibit the expression of the SARS-CoV-2 gene without causing side effects, thereby eliminating lung inflammation, and reported that miRNA cargoes in PDNVs were richer than those in ginger tissue, but lower than those in tRNAs [162]. However, naked miRNAs are easily and immediately disassembled by ubiquitous RNA enzymes, and thus can be actively secreted in various body fluids through vesicles or encapsulated within them, avoiding their early destruction by RNA enzymes, and thus participating in various physiological or pathological processes [163, 164]. For example, Xu et al. used ginseng-derived PDNVs as vectors to efficiently transfer 20 miRNAs into bone marrow mesenchymal stem cells (BMSCs), and were able to stimulate the neural differentiation of high-level BMSCs, suggesting that PDNVs can be used as delivery vectors to transfer plant-derived miRNAs into mammalian stem cells [78]. Thus, the unique ability of PDNVs to transport a variety of RNAs highlights their potential as platforms for drug delivery. Moreover, PDNVs also contain many nucleic acids that are used as therapeutic agents, but how they affect uptake and cellular communication in recipient cells is currently unclear.

#### Metabolites

Studies have demonstrated that PDNVs carry secondary metabolites, including flavonoids, chlorophylls, and curcuminoids, with their composition varying according to the plant species of origin [123, 165]. It has been reported that when secondary metabolites are more lipophilic, they will be enriched in membranes rather than packaged in vesicles in an active manner [165]. PDNVs extracted from orange juice by Berger et al. were examined by metabolomics and found to contain a number of primary metabolites such as carbohydrates (glucose, fructose, sucrose) and amino acids (alanine, asparagine-isoleucine, threonine, leucine). Compared with orange juice, PDNVs accumulate leucine, threonine, formate, methanol, ethanol, and sn-glycero-3-phosphocholine [140]. Cao et al. reported that curcumin, demethoxycurcumin, and bisdemethoxycurcumin in turmeric-sourced PDNVs are extremely low in content and unable to fulfill their biological functions, whereas dried turmeric contains a large amount of curcumin-like substances [166]. Cao et al. identified a large number of active constituents of ginger in ginger-derived PDNVs, such as 6-gingerol, and 6-sugoanol [72]. Chen et al. quantified the content of active small molecules in tea-derived PDNVs by high-performance liquid chromatography-tandem mass spectrometry (HPLC-MS/MS), and these small molecules included many antitumor polyphenols and flavonoids, which promote apoptosis and microbiota regulation [167]. Thus, metabolites of PDNVs may have potential therapeutic functions for human diseases.

In conclusion, current studies have reported that the components of PDNVs are lipids, proteins, RNA and some plant metabolites. They have a greater potential in the treatment of human diseases, which may work synergistically through the various types of components, and need to be studied in greater depth. On the other hand, the study of specific protein markers for PDNVs is not well developed, probably due to the complexity of plant species, and therefore the identification of generalized protein markers would improve the consistency and transparency of this field of research.

**Table 3:** The main biochemical contents of various PDNVs

Source	Lipid	Protein	Nucleic acid	Others	Ref.
Tomato	PS, PA, PG, PC, PE	/	/	/	[147, 148]
	Cholesterol esters (CE), Diacylglycerols (DAG), Free fatty acid (FFA), PC, PE, Triacylglycerols (TAG)	/	/	/	
Aloe	GlcCer, Cer, PA, PC	135	/	Aloe-emodin, aloesin β-sitosterol	[75, 150]
	PA, Digalactosyl diacylglycerol(DGDG), Monogalactosyl monoacylglycerol (MGDG),	/	/	/	
Orange	MGDG, DGDG, PG, PI, PA, PE, PC	/	/	Carbohydrates (glucose, fructose, sucrose); amino acids (alanine, asparagine isoleucine, threonine, leucine)	[140]
Arabidopsis thaliana	GIPC, PA	/	/		[151]
Garlic chive	PC, PE, MGDG, DGDG, PA, PG	922	/	/	[158]
Ginseng	/	3129	/	amino acids, organic acids	[76]
Ginger	PC, MGDG, DGDG	Low content	125 miRNAs	6-gingerol, 6-shogaol	[72]
Lemon	/	580	/	/	[157]
*Arabidopsis*	/	1438	/	/	[94]
Grape	PA, PE, MGDG, DGDG, PC, PI, PS, Lyso-phosphatidylcholines (LPC), Lyso-phosphatidylethanolamines (LPE), Lyso-Phosphatidylglycerol(LPG)	28	95 miRNAs	/	[129]
Grapefruit	PC, PE, PI, PS, PA, MGDG, DGDG, LPC, LPE, LPG	137	/	naringenin	[130]
Citrus fruit	/	600–800	/	/	[159]
Ginger	PA, PC	/	106 miRNAs	/	[145]
11 edible fruits and vegetables	/	/	418 miRNAs	/	[56]
ginger	/	/	532 miRNAs	/	[162]
Orange juice	PE, PC, PI, PA	/	/	Carbohydrates, amino acids	[140]
Turmeric	PE, PC, fatty acid (FA), DAG, TAG,	/	/	curcumin, demethoxycurcumin and bisdemethoxycurcumin	[166]
Tea leaf	PC, Phosphatidylmethanol (Pme), Phosphatidylethanol (Pet), Triglyceride (TG), PA, Diacylglycerol (DG), Monogalactosyldiacyglycerol (MGMG)	446	/	epigallocatechin gallate (EGCG), vitexin-2-*O*-rhamnoside, vitexin, myricetin-3-*O*-rhamnoside, kaempferol-3-*O*-galactoside, myricetin	[167]

### Stability and preservation

As with mammalian-derived EVs, the stability and optimal preservation of PDNVs is critical for the subsequent evaluation of in vivo efficacy. And PDNVs also require good stability and suitable preservation conditions during preparation, storage and transportation. Relevant studies have been conducted to discuss the optimal storage conditions and stability of PDNVs, and their stability has been tested by changing external factors such as temperature and pH.

#### Stability

Research indicates that PDNVs exhibit notable stability across different pH environments. For example, Zhang et al. investigated the behavior of ginger-derived PDNVs in simulated gastric and intestinal fluids. They observed a slight reduction in particle size under both conditions. In terms of surface charge, the zeta potential shifted from − 14.2 mV in a near-neutral PBS solution to − 7.3 mV in intestinal-like fluid, and to a mildly positive value (0.26 mV) under acidic, stomach-like conditions. These findings suggest that PDNVs maintain structural stability in both gastric and intestinal environments, with changes in zeta potential reflecting their inherent physicochemical characteristics [72]. To simulate in vivo conditions, the particle size of grapefruit-derived PDNVs was tested under physiological conditions (37 °C), which were reduced and weakly positively charged under acidic conditions and unchanged and negatively charged (compared with the size of PDNVs in water) under alkaline conditions, and PDNVs were found to be highly resistant to digestion by pepsin solution and intestinal trypsin and bile extract solution resistance [130]. Additionally, PDNVs derived from edible tea flowers have demonstrated strong stability in various simulated physiological fluids, including gastric, small intestinal, and colonic simulants, as well as DMEM. Across these conditions, no significant alterations were observed in either particle size or zeta potential, suggesting that these vesicles maintain their structural integrity throughout the gastrointestinal tract (GIT) and in the bloodstream following either oral administration or intravenous injection [168].

However, sonication can also affect the structure and bioactivity of PDNVs, e.g., the particle number of apple-derived PDNVs disappears after sonication or boiling [65], and ginseng-derived PDNVs significantly reduce their ability to polarize macrophages after sonication [76]. Several studies have reported the use of sonication to de-load therapeutic agents [169, 170], so researchers should examine the morphology of PDNVs before and after loading. In addition, a review reported that the structure of PDNVs was damaged after treatment with the chemical deconstructant Triton X-100 [99]. Similar to mammalian EVs, PDNVs are usually stored at low temperatures for long periods of time to retain their intact membrane structure and biological activity. Chen et al. compared PDNV extracted from fresh ginger with PDNV extracted from thawed ginger stored at -80 °C, and reported that both had similar inhibitory effects on IL-1β secretion and auto-cleavage of caspase-1 (CASP1) [171]. The enhanced cytotoxicity of groundnut-derived PDNVs after storage at -80 °C has also been reported in the literature, but the reason for this is currently unknown [99]. Therefore, the stability of PDNVs from different plant sources needs to be further explored to ensure their uniform physicochemical properties.

#### Preservation

Since PDNVs have active ingredients of their own, storage conditions are critical. However, the storage conditions of PDNVs have been less explored, and most studies have extended the storage conditions of mammalian EVs to 4 °C or -20 °C for short-term storage, and − 80 °C for long-term storage, which is a widely used storage method for EVs [172]. However, storage at − 80 °C decreases the concentration of EVs and sample purity in a time-dependent manner, and the size of EVs increases, and the zeta potential changes [173]. In a report related to plant-derived PDNVs, Variya et al. placed Kaempferia parviflora-derived PDNVs at − 20 °C and − 80 °C for 8 weeks, and the PDNVs were more stable. Lipid hydrolysis of the PDNVs was observed during storage at room temperature and 4 °C [174]. In another study, PDNVs were found to be stable for more than 1 month in a 4 °C storage environment [80]. Edible ginger-derived PDNVs showed no significant differences in particle size and zeta potential after 25 days of storage at 4 °C [175]. The ISEV’s recommendation in 2013 was reportedly that samples be stored using PBS as a matrix and cryopreserved at -80 °C. However, in 2018, ISEV no longer promoted a specific method and instead recommended a specific protocol based on the nature of the different types of vesicles [52]. Therefore, the stability of PDNVs may also be influenced by storage temperature and shelf life, which can vary depending on their plant source. Currently, there is no standardized consensus on optimal storage conditions, as these parameters are likely contingent upon the intrinsic characteristics of the originating plant species.

For mammalian EVs, cryopreservation agents (CPAs) have been widely used for the freeze-drying storage of EVs as a way to improve their stability [176, 177]. However, few studies have been reported on plant-derived PDNVs. Kim et al. reported that freeze-dried ginseng-derived PDNVs were stable at room temperature for up to 60 days, the protein and total RNA concentrations were evaluated, and no major changes in their morphology were detected [77]. However, in this report, no CPAs were used; perhaps the chemical composition of the different types of CPAs has a potential effect on PDNVs, thus minimizing the effect of the additional components until the way in which PDNVs are stored is well researched.

In conclusion, multiple factors—including temperature, pH, chemical reagents, and sonication—can significantly impact the stability of PDNVs. Variations in storage conditions may alter their concentration and purity, ultimately influencing the biological functionality and therapeutic efficacy of PDNVs. However, there are few reports related to their storage conditions, and cryopreservation is still common, but also involves freeze-drying so that they can be preserved at room temperature. However, whether CPAs can be added during the freeze-drying process to improve long-term stability, as in the case of mammalian EVs, and whether the possible addition of chemical components has unknown effects on PDNVs needs to be studied in depth by researchers. The study of the stability and preservation conditions of PDNVs is crucial and is one of the key conditions for clinical translation.

## Uptake mechanisms, distribution, and biosafety

Whether PDNVs can be taken up by receptor cells is a key factor for their effective utilization after administration, and it is of great concern whether PDNVs, once in the body, can reach the site of the lesion to exert their therapeutic effects as well as whether they are safe and nontoxic to non-target organs.

### Uptake mechanisms of PDNVs and biodistribution

**Fig. 3:**
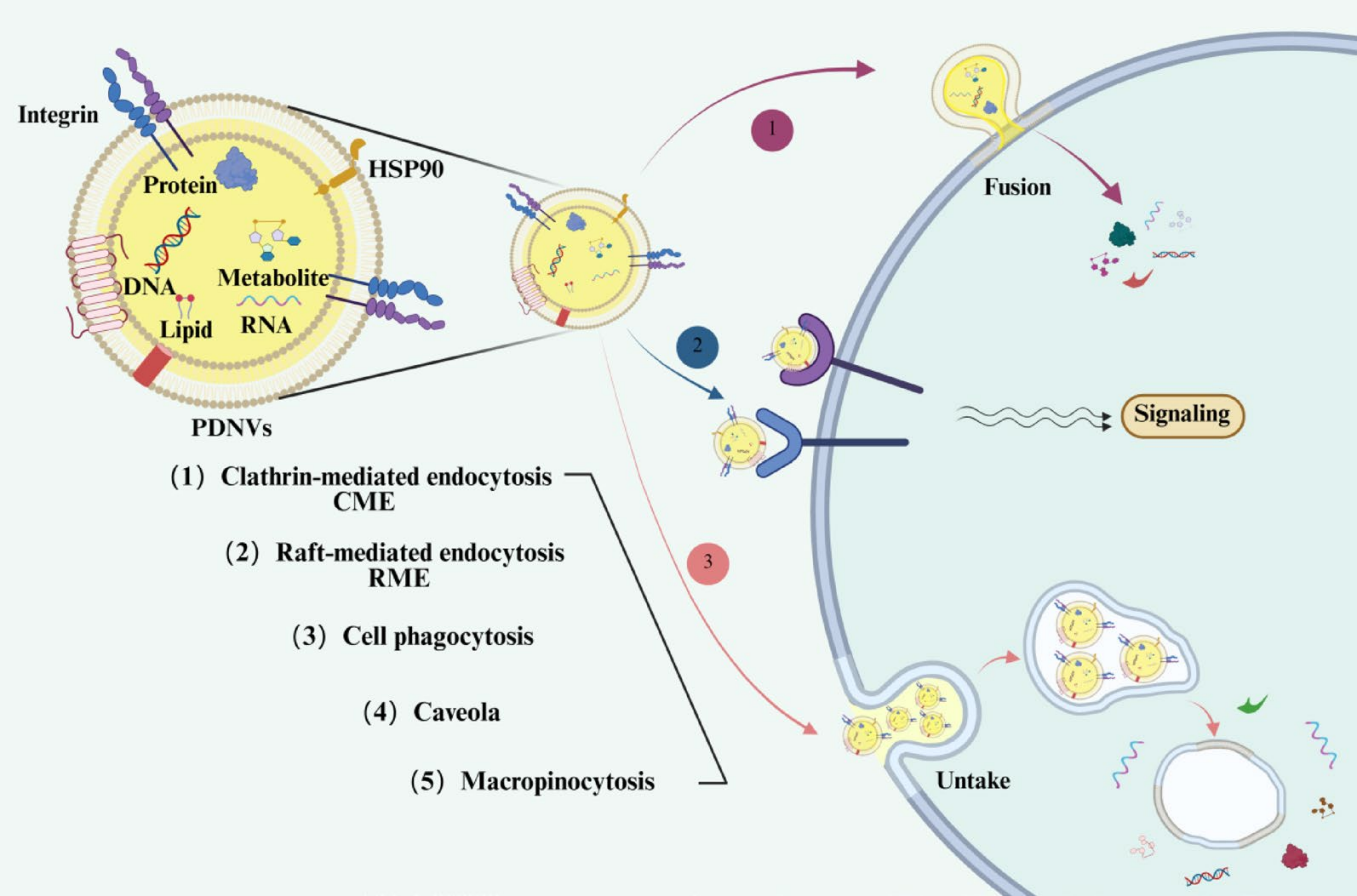
Elucidation of Multiple Pathways of Cellular Internalization. (1) Fusion with the target cell membrane; (2) internalization through endocytosis into target cells; (3) binding to receptors on the target cell membrane. Created with Biorender.com

PDNVs can act as therapeutic agents and drug carriers in their own right, so it is important to evaluate their transport and distribution in vivo. PDNVs have been reported to be internalized by a wide range of mammalian cells, such as tumor cells (breast, colon, melanoma, and oral cancer) [69, 88, 125, 175] and normal human skin keratinocytes (HaCat cells) [69, 80]. There are currently three mechanisms by which PDNVs enter recipient cells: the first is direct fusion with the recipient cell membrane and release of cargo into the cytoplasm. The second is internalization into the receptor cell by endocytosis, followed by release of the cargo into the cytoplasm. The third binds to receptors on the recipient cell membrane, initiating receptor-ligand interactions and downstream signaling cascades that activate the recipient cell [178] (Fig. 3). The mechanisms of the cellular internalization and in vivo biodistribution of PDNVs have also been reported in relevant studies. Ju et al. reported that the uptake of grape-derived PDNVs by CT26 cells was significantly attenuated by the addition of cytochalasin D, whereas the caveolae-mediated inhibitor of endocytosis, indomethacin, and the clathrin-mediated endocytosis inhibitor chlorpromazine did not affect the uptake of PDNVs, demonstrating an uptake mechanism that is mediated through macropinocytosis [129]. Macrophage RAW 264.7 cells take up onion-derived PDNVs via macropinocytosis and the caveolae-dependent endocytosis pathway [179]. Cholesterol-dependent pathways were found to be involved in the uptake of corn-derived PDNVs by colon26 cells by Sasaki et al. [180]. In another study, the mechanism of uptake of turmeric-derived PDNVs by RAW 264.7 cells was reported, and indomethacin was found to significantly inhibit the internalization of PDNVs, suggesting that PDNVs mediate entry into cells via caveolae-mediated endocytosis, and the effects of temperature (37, 20, and 4 °C) on the efficiency of cellular uptake were explored and found to be reduced at 4 and 20 °C, suggesting that PDNV uptake is energy dependent [181].

Transport proteins located on the surface of PDNVs may influence the mechanism of their internalization, and a preliminary investigation in which the surface proteins of garlic-derived PDNVs were digested with trypsin and then removed revealed that the uptake of PDNVs by HepG2 cells was significantly reduced and that endocytosis was dependent on the interaction between the surface protein II agglutinin of the PDNVs and CD98 on the HepG2 cells [182]. The specific lipids carried by PDNVs also affect their internalization efficiency [144], e.g., PC-rich grapefruit-derived PDNVs are preferentially internalized by Ruminococcaceae [145].

It is crucial to explore the translocation pathway and fate of PDNVs after their uptake by cells. Itakura et al. observed the organelle localization of grapefruit-derived PDNVs in HaCaT cells using confocal laser scanning microscopy (CLSM) and reported that co-localization occurred in endosomes/lysosomes after 6 h, indicating that PDNVs are degraded in lysosomes after their cellular uptake [183]. Cui et al. reported that when functional plant-derived ginger-derived PDNVs and porous ZIF-8 nanoparticles were used for the delivery of TNF-α siRNA, co-localization of the siRNA with lysosomes was observed after co-incubation with RAW 264.7 cells (red), which indicated that a large amount of the siRNA in the nanoparticles entered the lysosomes. After 12 h of incubation, the red fluorescence weakened, and green fluorescence was distributed in all parts of the cytoplasm, indicating that the siRNA effectively escaped from the lysosome [184]. However, few studies have focused on the mechanism of the intracellular fate of PDNVs, and elucidating the mechanism of their internalization, as well as the fate of PDNVs after their cellular uptake, will pave the way for the delivery of PDNVs as drug carriers or therapeutic agents.

### Distribution of PDNVs

The route of administration is primarily influenced by the disease type and the characteristics of the therapeutic vehicle; thus, different administration methods can significantly impact the biodistribution of PDNVs [165]. The following routes of administration of PDNVs-based nanoplatforms have been reported to deliver drugs to experimental animals, such as oral [153], intravenous, intraperitoneal [76], nasal [185] and transdermal administration [186] (Table 4).

Owing to its simplicity and ease of administration, oral administration is still the preferred mode of administration for most drugs [187]. Ginger-derived PDNVs are more readily absorbed by intestinal bacteria in the colon because of their rich PA content, and Cui et al. reported that nanoparticles encapsulated via ginger-derived PDNVs were more concentrated in colon tissue (labeling of siRNA in nanoparticles with Cy5.5), but fluorescent signals in other major organs (heart, liver, spleen, lungs, and kidneys) were negligible throughout the assay period, suggesting that they were metabolized in the gastrointestinal tract after oral administration [184]. In contrast, in another study in which DIR-labeled ginger-derived PDNVs were used after oral administration to mice, DiR fluorescence signals were detected mainly in the liver and mesenteric lymph nodes, and the fluorescence intensity in the liver peaked at 12 h; however, no fluorescence signals were detected in the lungs, spleen or other organs [75]. Using DIR-labeled lemon-derived PDNVs given orally to mice with gastric cancer, the mice were imaged at 6 h and 24 h and 3D organ reconstruction was performed, revealing that their PDNVs were predominantly found in gastrointestinal organs [87]. Berger et al. To determine the biodistribution of orange juice-derived PDNVs during digestion, mice were given 150 µg of DIR-labeled PDNVs by gavage, and the distribution of DIR-PDNVs fluorescence in metabolic organs was determined 6 h later, with a gradient of fluorescence from the jejunum-ileum to the cecum-ascending colon in the intestine [140]. Zhang et al. explored the stability of PDNVs in gastro-like and intestinal-like solutions, and analyzed the size and surface charge of PDNVs in water, and in gastro-like or intestinal-like fluids. The results revealed that the size of PDNVs can change with pH, and an evaluation of the charge of PDNVs revealed that, in comparison with that of PDNVs in water, the negative charge of PDNVs in a gastro-like solution significantly decreased, whereas the charge of most of the PDNVs in an intestinal-like solution did not significantly change, whereas that of a few PDNVs with a reduced charge therefore increased their resistance to gastrointestinal tract digestion [188]. Overall, PDNVs exhibited strong resistance to degradation in gastric and intestinal fluids, thus allowing them to be taken orally and applied to digestive disorders.

Intravenous injection enables rapid entry of therapeutic agents into systemic circulation, leading to a faster onset of action while bypassing intestinal absorption. In a breast cancer mouse model, intravenous administration of free DiR and DiR-labeled bitter melon-derived PDNVs demonstrated that free DiR was rapidly cleared from the body and primarily metabolized by the kidneys. In contrast, DiR-loaded PDNVs exhibited predominant accumulation in the liver and demonstrated some degree of tumor-targeting capability, likely due to the enhanced permeability and retention (EPR) effect [125]. However, most PDNVs rely on passive targeting to reach pathological sites, which limits their specificity. To overcome this limitation, surface modification of PDNVs has been proposed to enhance their targeting efficiency following intravenous administration. For example, intravenous injection of DIR-labeled ginger-derived PDNVs and folic acid (FA)-modified PDNVs showed that the fluorescence intensity of FA-PDNVs at the tumor site was 2.8-fold greater than that of PDNVs, suggesting that FA-PDNVs are able to target tumors, possibly through an active (FA-FA receptor interaction) mechanism, and that the two show significant fluorescence in the liver, possibly because the lipid component digalactosyl diacylglycerol derived from PDNVs is recognized by the asialoglycoprotein receptor expressed in hepatocytes [175]. Liu et al. functionalized the surface of grapefruit-derived PDNVs with cRGD peptides to target integrin αvβ3 receptors expressed on glioma cells, aiming to enhance their specificity and targeting efficiency. Following tail vein injection, these modified PDNVs demonstrated extended circulation time, effective BBB penetration, and accumulation within glioma tissues. Additionally, the engineered PDNVs exhibited improved retention in metabolic organs, thereby contributing to prolonged systemic circulation [189]. Therefore, it is important to engineer modifications to PDNVs that can prolong their systemic circulation time and effectively target lesions via intravenous injection.

In another study, Raimondo et al. injected free DIR and DIR-labeled lemon-derived PDNVs intraperitoneally into a mouse model of multiple myeloma, and free DIR never reached the tumor site at the three time points of 15 min, 1 h, and 24 h. At 24 h after the injection of DIR-PDNVs, significant fluorescence was also detected at the tumor site [157]. To determine the biodistribution of ginseng-derived PDNVs in vivo, Cao et al. evaluated the effects of DiR-labeled PDNVs via different routes of administration and reported that 72 h after intraperitoneal and intravenous injection, most DiR-labeled PDNVs were located in the liver and spleen. In contrast, PDNVs administered via intragastric injection were located mainly in the stomach and intestine, and no signals were detected in the lungs, heart, kidneys or brain. This suggests that the size and structural characteristics of PDNVs may contribute to their enhanced stability and prolonged retention in systemic circulation [76].

Several studies have also indicated that nasal administration can facilitate drug delivery across the BBB [190]. For instance, FA-encapsulated grapefruit-derived PDNVs were shown to rapidly reach the brain following nasal administration [185]. Transdermal administration is a mode of drug delivery in which the drug is able to penetrate the epidermal barrier, be absorbed by the skin and enter the site of action [191]. Broccoli-derived PDNVs reportedly have high lipophilicity and high ability to penetrate deep into skin tissues [186]. Another study reported that the skin penetration efficiency of cucumber-derived PDNVs mixed with lipophilic drug substitutes was two times greater than that of a blank control, and these PDNVs significantly penetrated the dermis [192].

In conclusion, different injection methods as well as different sources of PDNVs affect the biodistribution of PDNVs, and currently also the in vivo distribution of PDNVs has been affected by engineering modifications of PDNVs, and the mechanisms of internalization and biodistribution are intricate and complex, thus further research is needed to explore.

**Table 4:** Different administrations of PDNVs

Administration approach	Plant source	Injection dose	Biodistribution	Ref.
Oral	Ginger	PDNVs at 200 nmol siRNA dose	Colon	[184]
	Ginger	50 mg of PDNVs	Liver and mesenteric lymph nodes	[75]
	Lemon	50 µL(50 µg) of PDNVs	Gastrointestinal organs	[87]
	Orange	150 µg of PDNVs	Jejunum-ileum to the cecum-ascending colon	[140]
Intravenously (i.v.)	Ginger	200 nmol of PDNVs	Liver and tumor	[175]
	Melon	/	Liver, spleen and tumor	[125]
	Grapefruit	1.7 mg/kg of PDNVs	Brain, liver, spleen, lungs and kidneys	[189]
Intraperitoneal (i.p.)	Limon	50 µg of PDNVs	Liver, spleen and tumor	[157]
	Ginseng	/	Liver and spleen	[76]
Intranasal	Grapefruit	10 µg (10 µL) of PDNVs	Brain	[185]
Transdermal	Broccoli	200 µL of PDNVs	Deeper skin layers	[186]

### Toxicity and immunogenicity of PDNVs

In recent years, PDNVs have attracted much attention from researchers because of their natural origin and good biocompatibility. However, to fully utilize the potential of PDNVs in nanomedicine, adequate attention needs to be given to safety and toxicological issues [193], and a comprehensive safety assessment of PDNVs is a more critical step to facilitate their translation to the clinic. Comparing the synthesized nanoparticles and liposomes, they will mostly accumulate in the liver and spleen, causing toxicity. In the case of exosomes secreted by mammalian cells, they can contain immunogenicity introduced by the secreting cells [194], Unlike PDNVs, which are derived mostly from edible plants, they are free of zoonotic or human pathogens [39, 195], therefore, they are less toxic to humans and more suitable for therapeutic use.

Strawberry-derived PDNVs internalized by mesenchymal stromal cells (MSCs) did not negatively affect their cell viability and also protected the cells from oxidative stress, as reported in previous studies [67]. The use of cabbage-derived PDNVs with different particle numbers did not significantly affect the reduction in cell viability after 72 h of incubation with human cells or mouse macrophages [109]. Zhang et al. compared commercially available liposomes with ginger-derived nano-liposomes (GDLVs) to assess their biocompatibility after co-incubation with RAW 264.7 cells and colon cancer cells for 24 h (up to 200 µM), respectively, and found that the GDLVs did not change the cell viability significantly, whereas the commercially available liposomes significantly reduced the RAW 264.7 and colon cancer cells in a concentration-dependent manner viability. Moreover, at the same lipid concentration, the effect of GDLVs on intestinal barrier function was less than that of commercially available liposomes, which are toxic to intestinal epithelial cells to a certain extent. The potential side effects of GDLVs in vivo were also evaluated by collecting blood from GDLVs-treated mice and performing blood analyses and biochemical analyses, which revealed no significant differences compared with those of the PBS control group. These results suggest that GDLVs are non-toxic compared with commercially available liposomes and have the potential to replace liposomes as a new drug delivery vehicle [153].

Yang et al. administered lemon-derived PDNVs to BALB/c nude mice intravenously (50 µg/mouse), and after 2 weeks, morphological analysis of heart, liver, spleen, lung, and kidney tissue sections using H&E staining to assess the biosafety of the PDNVs revealed that none of the tissues detected had any significant abnormalities [87]. Chen et al. evaluated the safety of cucumber-derived PDNVs by hemolysis tests, proinflammatory cytokine assays, and H&E staining and reported that the hemolysis rate of PDNVs was less than 5%, suggesting that intravenous injection of PDNVs did not destroy erythrocytes. Moreover, there was no significant difference in the serum levels of pro-inflammatory cytokines (including TNF-α and IL-1β) between the mice in the PDNV-treated group treated with i.v. injections and those in the PBS-control group, and minimal damage was observed by H&E staining of organs. These results therefore suggest that cucumber-derived PDNVs are nontoxic in vivo [196]. To explore the potential in vivo cytotoxic effects of grapefruit-derived PDNVs, Wang et al. quantified proinflammatory cytokines and liver injury indices and used DOTAP: DOPE liposomes as a control and reported that alanine aminotransferase and aspartate aminotransferase were significantly elevated in the serum of mice treated with DOTAP: DOPE liposomes, whereas they were not elevated after treatment with GNVs receiving doses of 50 nmol or higher. Histologic analysis of tissues from PDNV-treated animals revealed no pathological changes or necrosis in the lungs, kidneys, liver, or spleen compared with those in tissues from untreated mice. When PDNVs are injected intravenously into pregnant mice, they do not cross the placental barrier, indicating that PDNVs are also safe for the fetuses of pregnant women and may act as useful drug delivery vehicles for pregnant women [80].

Overall, nanoplatforms based on PDNVs have excellent biocompatibility, but it is also necessary to consider whether engineered modified PDNVs alter their biological properties or safety, which deserves more in-depth studies by researchers. In addition, PDNVs from different sources have different compositions and properties, so a comprehensive safety assessment is needed before their administration. Since PDNVs are derived mainly from edible plants, the oral route of administration should be prioritized. Owing to the different drug delivery routes of PDNVs and some unknown bioactive components of PDNVs, their biosafety and toxicity are still major challenges and more critical steps toward the clinic.

## The role of PDNVs in diseases

The use of active substances (e.g., polysaccharides, phenolics, and terpenoids) contained in plants for the prevention and treatment of diseases has been applied for a long time [197], among which PDNVs, which are natural products of plant cells, carry a large number of active substances that mediate cell-cell communication and perform biological functions [198], and have pharmacological effects similar to those of the original plants, which are also considered to be relatively safe. PDNVs have been reported to have anti-inflammatory [199], anti-tumor [200], antiviral [201], liver disease [202] and regenerative effects [55] and can be modified, engineered, or combined with other therapeutic agents and nanomedicines as a way to enhance therapeutic capabilities for specific diseases. In this section, the possible roles of PDNVs in human health are discussed (Table 5).

### The role of PDNVs in anti-inflammatory disease

Inflammation is the physiological response of an individual to a series of physical, chemical or infectious stressors, which primarily provide localized protection for the body, but excessive or prolonged inflammatory responses may be harmful to the body and are now proven to trigger a wide range of chronic diseases, especially inflammatory diseases [203, 204]. This has developed into a widespread global health issue, impacting millions of individuals affected by diverse pathological conditions [205]. Numerous studies have now reported that PDNVs can exert anti-inflammatory effects through mechanisms such as increasing anti-inflammatory cytokines and decreasing pro-inflammatory cytokines [90]. Therefore, PDNVs can be used as inflammatory immunomodulators to treat inflammation.

**Fig. 4:**
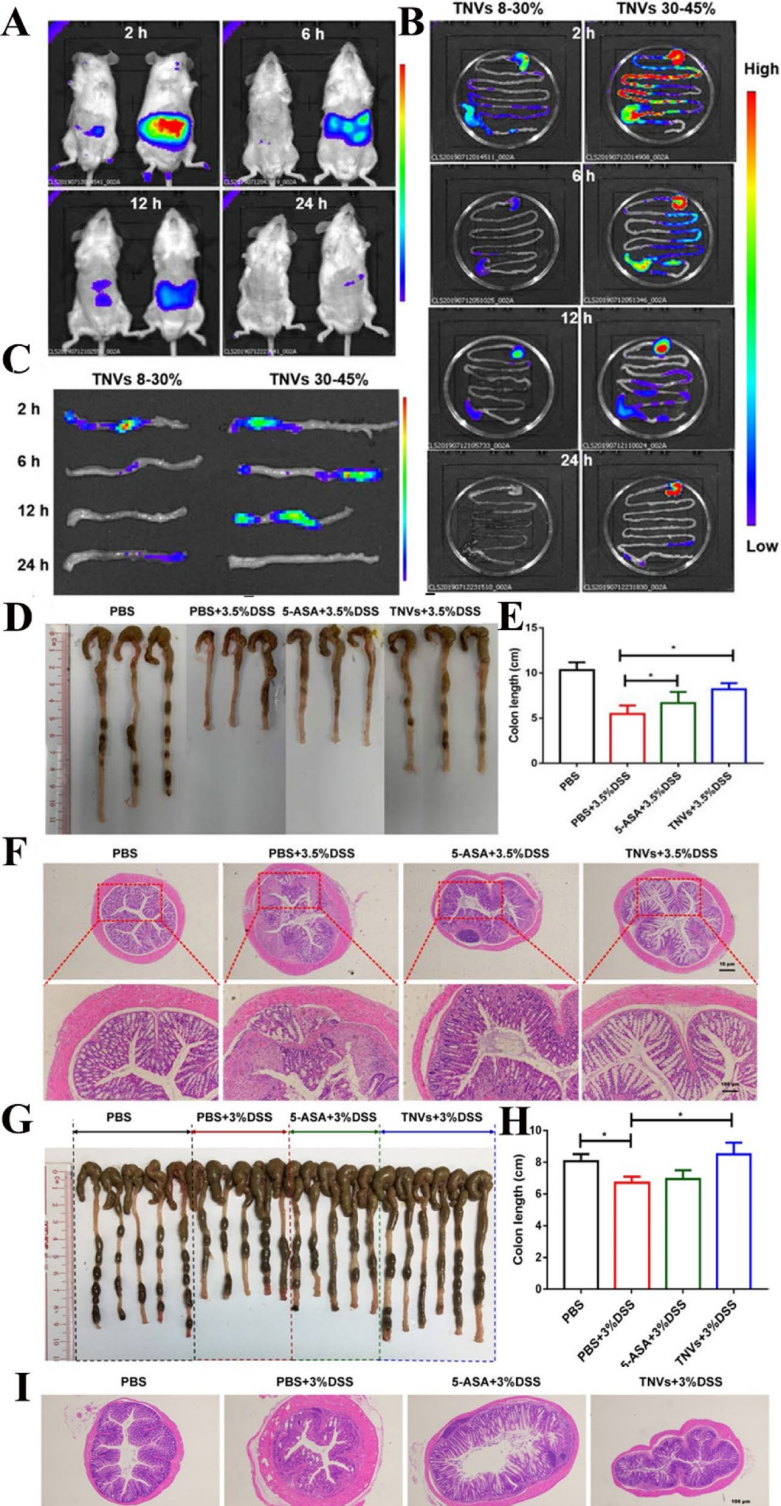
In vivo and ex vivo distribution of DiR-labeled TNVs and TNVs alleviated colitis-related symptoms in DSS-induced colitic mice.** (A)** Whole body images of the mice at 2, 6, 12 and 24 h after oral administration of TNVs originating from 8%-30% (left) and 30%-45% (right) band. (**B)** The distribution of DiR-labeled TNVs in the gastrointestinal tract at different time points. (**C)** The distribution of DiR-labeled TNVs in the distal colons at different time point. (**D-E)** Representative images of colons and average colon length from different groups. (**F)** Representative histological sections of distal colons stained with H&E (4× and 10× magnification). Scale bar: 10 μm. **(G-H)** Representative images of colons and average colon length from different groups. **(I)** histological sections of distal colons stained with H&E (4× magnification). Scale bar: 100 μm. Reprinted with permission from Ref [166]

Ulcerative colitis (UC) is a type of inflammatory bowel disease (IBD) that presents as idiopathic chronic intestinal inflammation. Conventional treatment mainly regulates pro-inflammatory and anti-inflammatory cytokines, but repairing the intestinal barrier, regulating the bacterial flora and correcting mucosal immune dysregulation remain therapeutic difficulties. Gao et al. used differential centrifugation to isolate and DGC-purified turmeric-derived PDNVs (TNVs) obtained from 8% to 30% (left) and 30%-45% (right) sucrose solutions labeled with DIR to validate their distribution ex vivo and in vivo in mice with acute colitis induced by dextran sulfate sodium (DSS) by gavage2,6 ,12 and 24 h. The fluorescence intensity reached a maximum after 6 h according to fluorescence imaging, followed by a gradual decrease and even disappearance (Fig. 4A). The in vitro distribution presented a more pronounced distribution of 30%–45% PDNVs than 8%–30% PDNVs in both the gastrointestinal and distal colon (Fig. 4B-C). Colonic shortening is a key factor in assessing the severity of inflammation during the development of UC. In DSS-induced acute colitis, colon length shortened to 5.5 ± 0.94 cm vs. 9.3 ± 0.85 cm in healthy mice. TNVs treatment restored colon length to 8.2 ± 0.56 cm, outperforming the positive drug group (6.7 ± 0.43 cm), indicating intestinal mucosa protection (Fig. 4D-E). H&E staining revealed reduced inflammatory cell infiltration and crypt damage in TNVs-treated mice (Fig. 4F). In chronic colitis, TNVs also prevented colon shortening (Fig. 4G-H) and mitigated inflammation, epithelial damage, and goblet cell loss (Fig. 4I). These results suggest that TNVs restore the damaged intestinal epithelial barrier and exert anti-colitis efficacy [166].

Kim et al. reported that ginseng-derived PDNVs suppressed the production of pro-inflammatory cytokines and promoted anti-inflammatory activity within the immune microenvironment by inhibiting the NF-κB signaling pathway [206]. In a separate study, Cui et al. loaded TNF-α siRNA onto ginger-derived PDNVs and encapsulated them on the surface of porous ZIF-8 nanoparticles (EVs@ZIF-8@siRNA) to promote intestinal barrier repair through effective inhibition of targeted TNF-α by siRNA and modulation of intestinal hygiene committee balance [184]. Kawada et al. reported that three microRNAs (ath-miR166f, ath-miR162a-5p, and ath-miR162b-5p) in PDNVs of Atractylodes lancea origin may have anti-inflammatory activity, and that the treatment of BV-2 cells and mouse microglia with these microRNAs significantly reduces nitric oxide levels to prevent the pro-inflammatory effects of LPS stimulation. These findings suggest that PDNVs are promising therapeutic agents for neuroinflammation [207]. Liu et al. Lyophilised Eudragit S100-encapsulated cabbage-derived PDNVs packaged in capsules prevented disruption in the GI tract and were better transported through the upper GI tract to the colon, showing strong therapeutic effects in a mouse model of colitis [208]. Kang et al. found that onion-derived PDNVs showed dose-dependent inhibition of pro-inflammatory factor secretion and modulated NF-κB phosphorylation in LPS-treated RAW 264.7 cells [90].

In conclusion, PDNVs exhibit remarkable potential in anti-inflammatory therapy and can exert anti-inflammatory effects through multiple mechanisms, including increasing the levels of anti-inflammatory cytokines, decreasing the levels of pro-inflammatory cytokines, and inhibiting the NF-κB pathway. These findings suggest that PDNVs, as immunomodulators of natural origin, have promising applications in the treatment of inflammatory diseases, especially chronic inflammation and neuroinflammation.

### The Role of PDNVs in Anti-Tumor

In recent years, medicinal plants have been demonstrated to play important roles in tumor therapy. The secreted PDNVs are rich in specific proteins, miRNAs, lipids, and natural active ingredients, which are highly stable, biocompatible, and tissue targeted and can be used as direct tumor therapeutic agents and drug delivery systems [209]. The synergistic application of PDNVs with other therapeutic methods is expected to lead to new breakthroughs in tumor therapy.

**Fig. 5:**
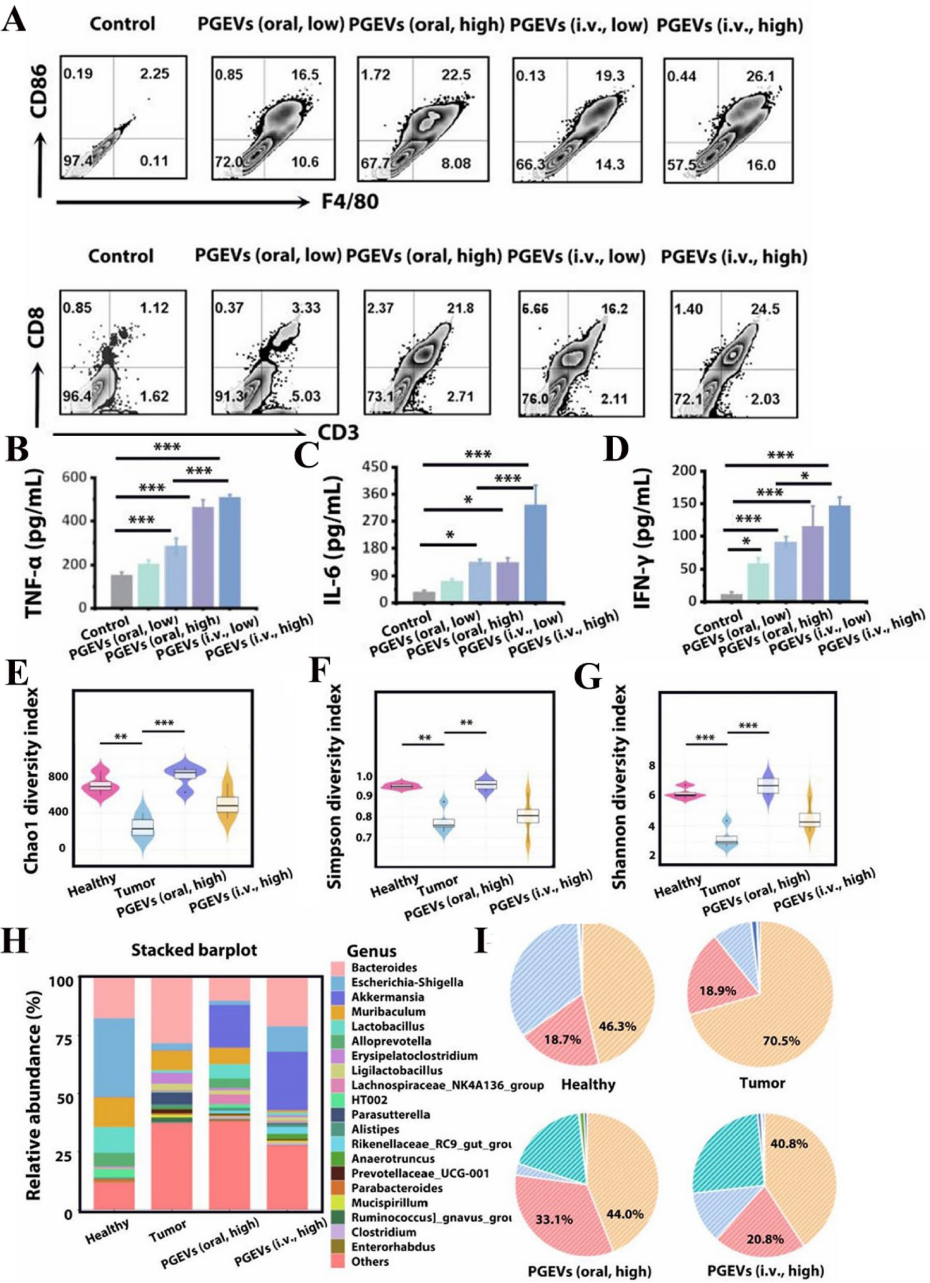
In vivo immune response evaluation and gut microbiota modulating effects of PGEVs.** (A)** Representative flow cytometry plots of CD86^+^F4/80^+^ cells and CD3 + CD8+ cells in tumors from mice after treatment with Control, PGEVs (oral, low), PGEVs (oral, high), PGEVs (i.v., low) and PGEVs (i.v., high). (**B-D)** Cytokine levels of TNF-α, IL-6 and IFN-γ in the serum of mice from different groups. (**E-G)** Alpha diversity were presented as violin diagrams of the Chao1, Shannon and Simpson indices. (**H-I)** The genus and phylum levels of gut microbiota compositions in different treated mice. Reprinted with permission from Ref [211]

Currently triple-negative breast cancer (TNBC) is clinically treated with chemotherapy as the main treatment option. However PDNVs derived from medicinal plants show great potential as novel biotherapeutic agents for cancer treatment by delivering their doped nucleic acids, especially microRNAs (miRNAs), to mammalian cells. Yan et al. isolated PDNVs derived from Brucea javanica and found that 10 functional miRNAs were delivered to 4T1 cells and inhibited 4T1 cell growth and metastasis by regulating the PI3K/Akt/mTOR signaling pathway and promoting ROS/caspase-mediated apoptosis, these miRNAs were able to inhibit tumor growth, metastasis, and angiogenesis in mammary carcinoma tumor-bearing mice while maintaining increased biosafety [210]. Yang et al. administered PGEVs (derived from Platycodon grandiflorus) to TNBC models via oral and intravenous routes at varying doses, observing significant tumor reduction. Flow cytometry analysis revealed increased CD86^+^F4/80^+^ cells across all PGEV-treated groups, with notably elevated CD3^+^CD8^+^ cells in intravenous (high/low dose) and oral high-dose groups (Fig. 5A), suggesting that PGEVs promote the reprogramming of TAMs to the M1-type, and that cytotoxic T-lymphocytes (CTLs) contribute to the reprogramming of TAMs to the M1-type either by direct killing of tumor cells or by modulating other immune cell activity to promote anti-tumor immunity. And cytokines (TNF-α, IL-6 and IFN-γ) associated with anti-tumor immune response were detected in mouse serum by ELISA, and PGEVs (venous, high/low) were significantly elevated in the treatment group (Fig. 5B-D), suggesting that it could induce a strong immune response response. Growing evidence suggests that the gut microbiota is highly correlated with the initiation, progression, and treatment response of various tumors, including TNBC. Therefore, 16 S rRNA gene sequencing was performed on mouse feces, and its results showed that the gut mycobiota abundance and diversity (Chao1/Simpson/Shannon index) was reduced in tumor control mice compared to the healthy group, which was significantly restored by PGEVs treatment ( Fig. 5E-G). At the genus level, PGEVs (especially in the oral group) decreased the abundance of the harmful bacterium Bacteroides and significantly increased the beneficial bacterium Lactobacilli (Fig. 5H). At the phylum level, the *Anaplasma phylum*, thick-walled phylum and Aspergillus phylum were the dominant phyla, and the ratio of the thick-walled phylum/Anaplasma phylum was greater in the oral and treatment groups than in the tumor control group (Fig. 5I). These results suggest that PGEVs treatment can increase the abundance and diversity of the intestinal microbiota and correct flora disorders, which may help inhibit tumor development [211].

Kim et al. isolated PDNVs derived from Perilla leaf and demonstrated their anticancer potential, showing significant inhibition of proliferation, migration, and invasion in MDA-MB-231 breast cancer cells [212]. In a related study, Yang et al. extracted PDNVs from *Curcumae Rhizoma* loaded with astragalus-derived compounds, reporting reduced cell viability in Caco-2 and HeLa cells to 42.58% and 33.34%, respectively, after 48 h of treatment [213]. Additionally, Yang et al. used UC to obtain PDNVs from Radix Polygoni Multiflori, which were found to inhibit the proliferation and migration of hepatocellular carcinoma cells, and bioinformatics analyses showed that PDNVs regulated the expression levels of numerous genes related to cancer and the cell cycle, and were able to target the liver tissue of mice [214]. Ma et al. obtained PDNVs derived from Hypericum perforatum and confirmed the presence of chrysin, as well as the fact that PDNVs exhibit potent photosensitizing properties, generate ROS upon light activation, and that in vivo PDNVs-mediated photodynamic therapy (PDT) significantly inhibits melanoma growth [215]. Cui et al. found that Citrus limonL-derived PDNVs had significant anti-tumor cellular effects on TNBC cells [216]. Lu et al. loaded DOX onto celery-derived PDNVs and found it to be more effective than conventional liposomes against tumors both in vivo and in vitro [217]. Han et al. combined ginseng-derived PDNVs with PD-1 monoclonal antibody for the treatment of breast and colon cancer, which significantly inhibited tumor growth and no significant systemic toxicity was observed, It also induced long-term anti-tumor immune memory against tumor recurrence [218]. Chen et al. administered PDNVs derived from tea flowers intravenously or orally to mice with breast cancer, modulated the gut microbiota, and inhibited breast cancer growth and metastasis [168].

In conclusion, PDNVs offer distinct advantages in cancer therapy by serving as carriers for functional biomolecules—such as microRNAs—that regulate tumor-associated signaling pathways, trigger apoptosis, and remodel the tumor microenvironment to enhance antitumor immune responses. Additionally, PDNVs contribute to the modulation of gut microbiota, alleviating dysbiosis and further suppressing tumor progression. Their inherent biocompatibility and ability to target specific tissues position PDNVs as promising candidates for drug delivery applications. In the future, the synergistic application of PDNVs with existing therapeutics is expected to bring breakthroughs in tumor treatment and promote the development of personalized precision medicine.

### The Role of PDNVs in regeneration and wound healing

PDNVs have been shown to promote the healing of chronic wounds; however, research on their specific roles and underlying mechanisms in this context remains limited [219]. Given that inflammatory regulation is critical to tissue regeneration, maintaining an optimal inflammatory balance is essential—insufficient inflammation may permit pathogen-induced tissue damage, while excessive or persistent inflammation can lead to lesion formation. Thus, modulating the inflammatory response represents an effective strategy to enhance tissue repair and regeneration [30].

Spinal cord injury (SCI) regeneration is a global medical challenge, with research focused on three key areas: nerve regeneration, inflammation suppression, and innovative applications of regenerative scaffolds. Zhang et al. isolated PDNVs from Lycium barbarum and found that they inhibited its inflammation and promoted neuronal differentiation, and that PDNVs significantly enhanced neural differentiation compared to stem cell-derived exosomes [220]. Natania et al. extracted PDNVs from P. peruviana fruits and found that they promoted the proliferation and migration of human dermal fibroblasts (HDF) and reorganized actin, and up-regulated collagen I and lowered MMP-1, suggesting that PDNVs regenerated HDF as well as remodeled HDF [221].

**Fig. 6:**
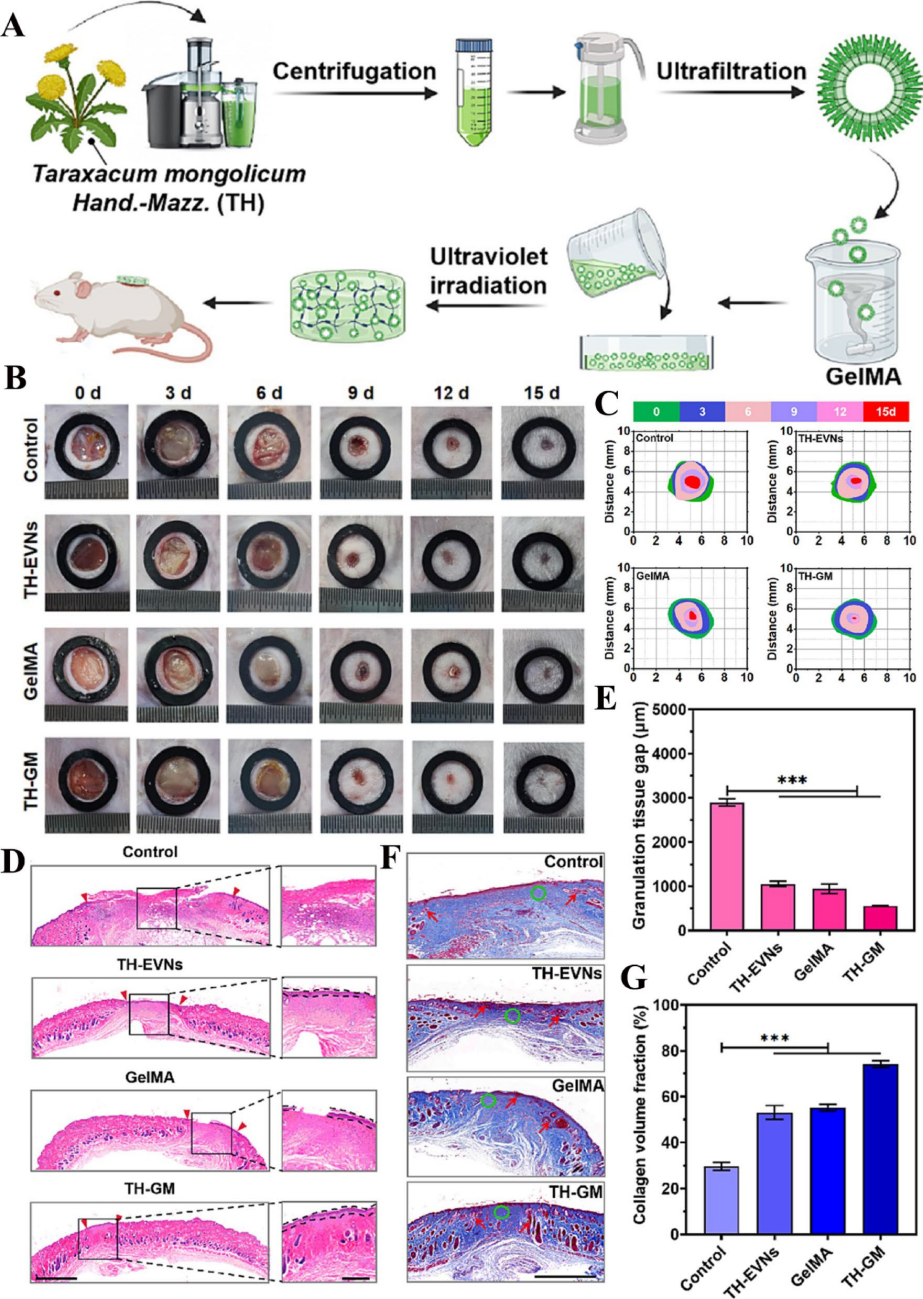
TH-GM hydrogels promote wound healing in vivo. **(A)** Schematic illustrations of photocrosslinked hydrogel loaded with dandelion-derived extracellular vesicle-like nanoparticles for *S. aureus* EVs-invasive wound. (**B)** Representative images of the healing process of *S. aureus* EVs-invasive wound. (**C)** Schematic diagrams of the wound healed by different treatments during 15 days. (**D)** Representative images of wound tissue sections in different groups stained by H&E on day 15. the two red arrows represent the unhealed area of the wound. Scale bar: 50 µm. (**E)** Quantification of the cross-sectional length of different wounds at day 15. (**F)** Representative images of wound tissue sections in different groups stained by Masson’ s on day 15. hair follicles are highlighted with red arrows and green circles highlight collagen deposits. Scale bar: 400 μm. (**G)** Quantification of collagen deposition density in different groups on day 15. Reprinted with permission from Ref[222]

Şahin et al. demonstrated for the first time, via an in vitro approach the activity of wheat-derived PDNVs during wound healing, with excellent proliferative and migratory effects on endothelial, epithelial, and dermal fibers, providing a completely new opportunity for therapeutic strategies in cutaneous wound healing [91]. Delayed wound healing due to bacterial infections is a clinical challenge, and exotoxins play important roles as key virulence factors. Plant-derived PDNVs have emerged as promising therapeutic agents for treating dermatologic conditions. Tan et al. isolated dandelion-derived PDNVs (TH-EVN) that specifically bind to Staphylococcus aureus (S. aureus) exotoxin to exert antitoxic activity, and developed gelatin methacryloyl hydrogels (GelMA) as PDNVs-loaded dressings (TH-GM) (Fig. 6A). A whole-layer wound model infected with Staphylococcus aureus was developed to evaluate the effect of TH-GM hydrogel on wound healing in vivo. As expected, the TH-GM-treated mice had faster wound closure than the other groups throughout the healing process (Fig. 6B-C). Compared with the other groups, the TH-GM-treated group presented significantly greater improvement in wound tissue, as evidenced by a more complete skin structure with newly formed epithelium (Fig. 6D-E). After 15 days of TH-GM treatment, there was no significant inflammation and relatively complete re-epithelialization of the wound. In addition, collagen deposition and organization were greater in the TH-GM group than in the other groups, as shown by Masson staining (Fig. 6F-G). These data suggest that TH-GM hydrogel may be an effective therapeutic strategy to promote healing of S. aureus exotoxin-infested wounds [222]. Valentino et al. isolated PDNVs from *Opuntia ficus-indica* fruit and demonstrated their protective effects against inflammation and oxidative stress in an in vitro model of chronic skin wounds [223]. Given that bacterial infections are a major barrier to effective wound healing, the development of safe and targeted antimicrobial therapies is critical. In this context, Saroj et al. assembled PDNVs derived from mint leaves with hydrogels developed from oxidized alginate and chitosan (MENV-HG) for the treatment of Gram-positive Micrococcus luteus and Gram-negative Escherichia coli-infested wounds, which showed satisfactory wound healing results [224].

In conclusion, PDNVs show remarkable potential in tissue regeneration and wound healing, especially in the regulation of inflammation, cell proliferation, and infection prevention. Combined with carriers such as hydrogels, PDNVs provide an innovative strategy for the treatment of complex wounds and infections and open a new direction for regenerative medicine.

### The role of PDNVs in liver diseases

Globally, more than 2 million people die of liver disease each year, accounting for 4% of all deaths. Despite the availability of many treatments, acute and chronic liver disease remains a medical challenge, and drug therapy has limited effect on end-stage liver disease. Liver transplantation is effective but there is a shortage of donors. Therefore, there is an urgent need to develop new therapeutic strategies for liver disease [225].

**Fig. 7:**
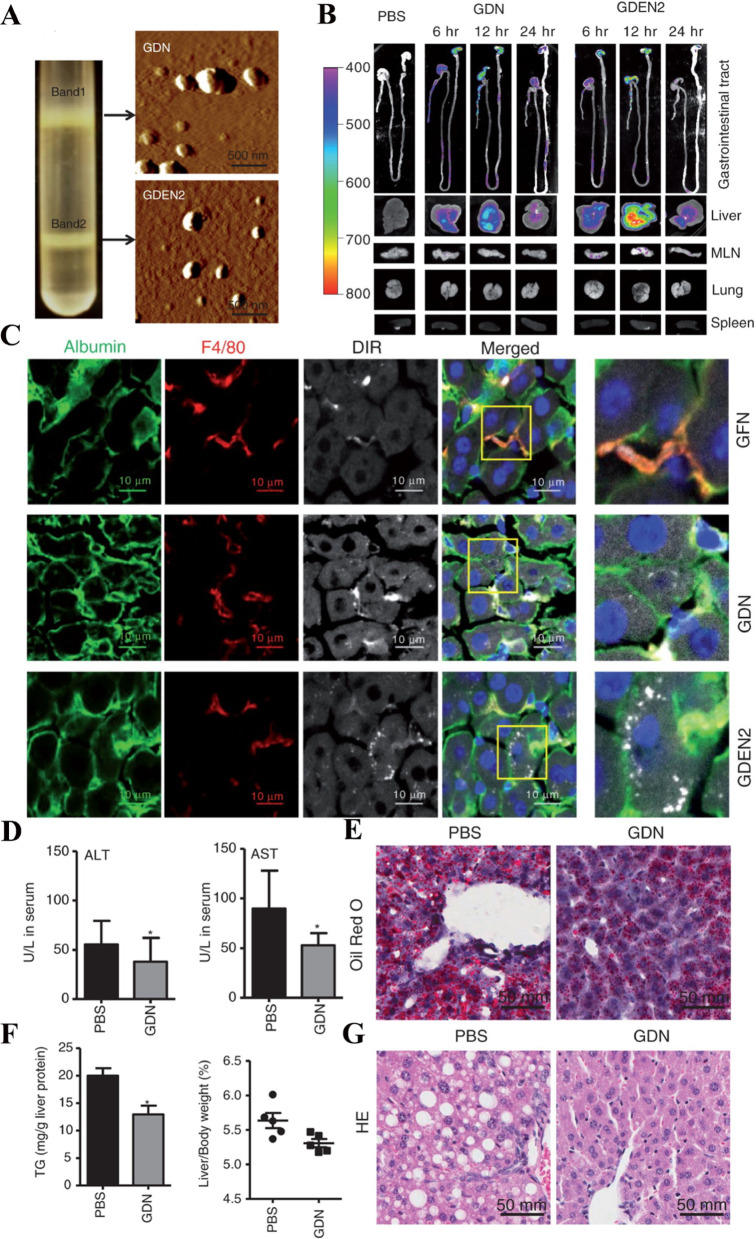
In vivo distribution of oral GDN and GDEN2 and oral GDN protects against alcohol-induced liver injury.** (A)** Two bands from sucrose-banded ginger rhizome root derived samples were formed after gradient ultracentrifugation (left). GDN and GDEN2 particles were visualized by atomic force microscopy (AFM, right). (**B)** In vivo imaging of trafficking of GDN and GDEN2. (**C)** Hepatocytes taking up DIR labeled GDN. Six hours after the administration, frozen liver sections were examined by confocal microscopy for DIR /Albumin F4/80 cells. Scale bar: 10 μm. (**D-G)** Eight-week-old male C57BL/6J mice fed a liquid diet containing 5% ethanol daily for 7 days. Starting on day 7, the mice were gavaged with GDN (50 mg/day) or PBS as a control daily while continuing the feeding of the 5% ethanol diet until day 14. On day 14, mice were fed with 30% instead of 5% ethanol and gavaged with a final dose of GDN at 9 h post-ethanol administration. The mice were euthanized and assessed for (**D**) levels of ALT and AST in serum, (**E**) neutral triglycerides and lipids using Oil red stain, Scale bar: 50 μm. (**F**) Liver triglyceride (TG) levels, ratios of liver/body weight, and (**G**) H&E-stained sections of livers from mice pretreated with PBS or GDN. Scale bar: 50 μm. Reprinted with permission from Ref [76]

The liver is subjected to a variety of different biological insults on a daily basis. The induction of cytoprotective enzymes, including antioxidant enzymes and carcinogen detoxifying enzymes, is essential for maintaining homeostasis within the liver and preventing damage from absorbed endotoxins. Zhuang et al. isolated PDNVs from ginger using sucrose gradient centrifugation, which was predominantly distributed at the 8%/30% interface (GDN, 386.6 nm) and the 30%/45% interface (GDEN2, 294.1 nm), and confirmed its size and integrity by AFM (Fig. 7A). To investigate tissue distribution, ginger-derived PDNVs were labeled with DiR and administered orally. Fluorescence imaging revealed a strong DiR signal predominantly in the liver and mesenteric lymph nodes (MLNs), with negligible detection in other organs such as the lungs and spleen (Fig. 7B). The presence of DIR-labeled ginger-derived PDNVs in the liver was further confirmed by confocal immunostaining for albumin, suggesting that hepatocytes are the primary cells targeted by PDNVs. Albumin^+^ hepatocytes were specific for ginger PDNVs, whereas PDNVs from grapefruit co-localized with F4/80^+^ liver Kupffer cells but not albumin^+^ hepatocytes (Fig. 7C). Elevated serum alanine aminotransferase (ALT) and aspartate aminotransferase (AST) levels were observed in a mouse model of alcohol-induced liver injury. However, histologic analysis revealed the accumulation of hepatic lipid droplets in ethanol-fed mice, which was significantly reduced in GDN-treated mice (Fig. 7D-E). Hepatic triglycerides and liver weights were significantly lower and pathological conditions, including reduced steatosis, fat accumulation, and inflammation, were improved in GDN gavage-treated mice compared with alcohol-fed mice (Fig. 7F-G). These results indicate that GDN has a protective effect against the development of alcoholic liver injury in mice [75]. Using seven commonly used mushrooms to extract PDNVs, Liu et al. reported that the consumption of only mushroom-sourced PDNVs inhibited NLRP3 inflammatory vesicle activation by blocking inflammatory vesicle formation in primary macrophages and significantly attenuated D-galactosamine (GalN)- and lipopolysaccharide (LPS)-induced acute liver injury in mice [202]. Kim et al. found that pomegranate-derived PDNVs prevented alcohol-induced liver injury and leaky gut [226]. Kim et al. found that hemp sprout-derived PDNVs prevented the reduction of intestinal barrier proteins and elevated endotoxin levels in a mouse model of non-alcoholic fatty liver disease (NAFLD) and alleviated leaky gut and hepatic fibrosis by inhibiting oxidative stress and reducing fibrosis marker proteins [227]. In a separate study, Zhao et al. developed garlic-derived PDNVs that significantly improved liver function and mitigated liver injury induced by LPS/D-GalN in mice [81].

In conclusion, PDNVs exhibited protective effects against liver injury in animal models, including attenuating alcoholic liver injury, inhibiting inflammatory vesicle activation, alleviating non-alcoholic fatty liver disease, and ameliorating LPS-induced liver injury. These PDNVs act by targeting hepatocytes or modulating immune cell functions and have good tissue distribution properties. In the future, PDNVs are expected to become an innovative therapeutic tool for liver disease, but their clinical applications still need further research and validation.

### The role of PDNVs in brain diseases

The global prevalence of neurological diseases is increasing, but the development of brain-targeted nanoparticle technology is facing challenges, mainly because the BBB restricts the access of nanoparticle therapies to the brain [228]. PDNVs, however, have become novel nanocarriers for the treatment of brain diseases and have attracted increasing attention because of their high yield, biocompatibility, low immunogenicity, and ability to penetrate the BBB [229, 230].

**Fig. 8:**
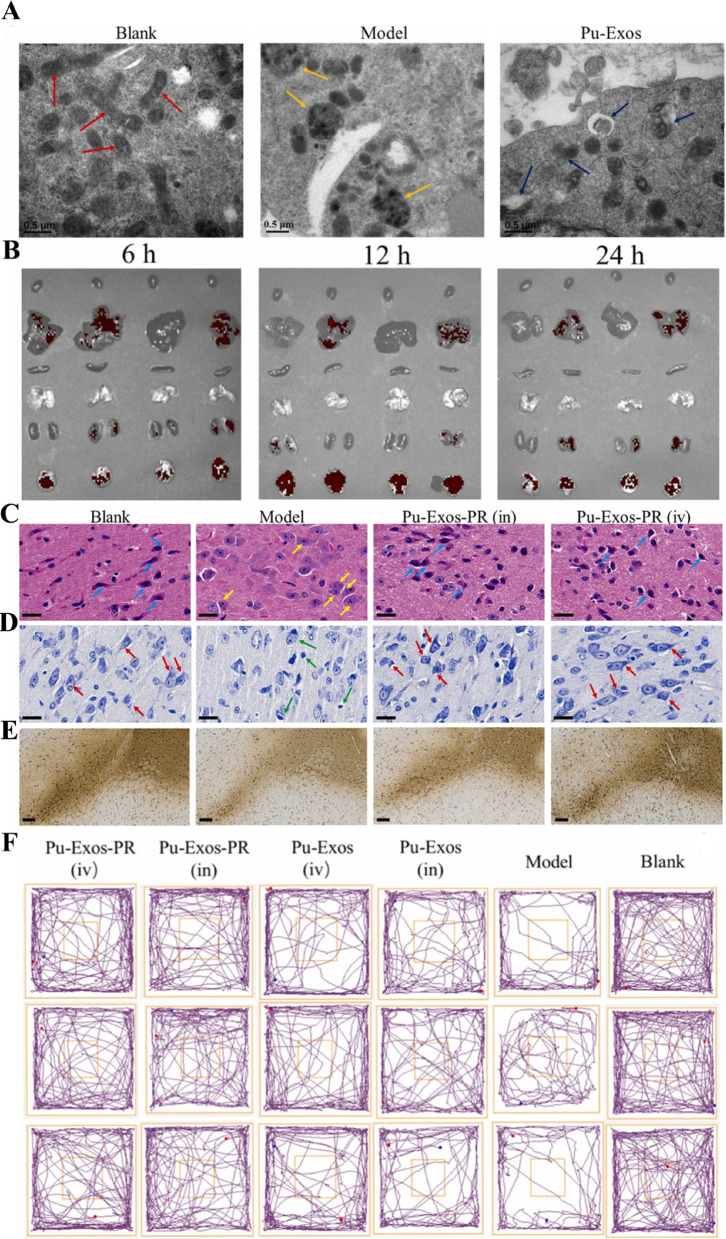
Improvement of mitochondrial dysfunction in SH-SY5Y cells by Pu-Exos and evaluation of in vivo delivery and efficacy of Pu-Exos-PR.** (A)** TEM images of cell sections from the blank, model and Pu-Exos-treated groups. (**B)** Ex vivo biodistribution of Pu-Exos and Pu-Exos-PR (Pu-Exos(in), Pu-Exos(iv), Pu-Exos-PR(in), and Pu-Exos-PR(iv) in the 6 h, 12 h, and 24 h images, respectively, from left to right). (**C-D)** H&E staining and (**C**) Nissl staining (**D)** Scale bar: 100 μm. (**E)** TH immunohistochemistry staining. Scale bar: 100 μm. (**F)** Motion trails of PD mice in the open field test Reprinted with permission from Ref [231]

Parkinson’s disease (PD) is the second most common neurodegenerative disorder characterized by loss of nigrostriatal dopaminergic neurons, cognitive decline and behavioral deficits. Although widely investigated, PD therapeutic strategies based on cellular heterogeneous nanomaterials still face challenges such as potential toxicity, immunogenicity, limited drug-carrying efficiency, and insufficient biobarrier penetration. In a pioneering study, Xu et al. first discovered that PDNVs (Pu-Exos) derived from Pueraria lobata can penetrate cell membranes and endosomal barriers to deliver biomolecules to SH-SY5Y cells. TEM images showed that the model group had reduced mitochondrial numbers, swollen mitochondria, and disrupted cristae structures (indicated by yellow arrows), while the Pu-Exos group exhibited restored mitochondrial numbers, partially intact cristae structures, and autophagosomes in contact with mitochondria (indicated by blue arrows), indicating that Pu-Exos improved mitochondrial dysfunction in SH-SY5Y cells (Fig. 8A). The ternary ligand DSPE-PEG-RVG-modified Pu-Exos-PR optimized brain-targeted delivery by reducing reticuloendothelial system(RES) clearance and non-specific uptake. Six hours after intravenous administration, its brain accumulation was 1.45 times higher than that of other groups (Fig. 8B). To assess the protective effect of Pu-Exos-PR on dopamine (DA) neurons, PD model mice were administered Pu-Exos-PR intravenously or intranasally. H&E staining showed that DA neurons in the control group had normal morphology and strong staining (indicated by blue arrows), DA neurons in the model group were reduced in number and exhibited nuclear condensation and swelling (indicated by yellow arrows), while DA neurons in the Pu-Exos-PR treatment group had restored normal morphology and reduced cellular degeneration (Fig. 8C). In addition, Nissl staining showed that neurons in the control group had normal morphology, with uniform cytoplasmic staining and abundant Nissl vesicles (indicated by red arrows); in the model group, Nissl vesicles were reduced and cell atrophy was observed (indicated by green arrows). In the treatment group, the density of Nissl vesicles increased, indicating improved DA neuron function (Fig. 8D). In addition, compared with the other treatments, the administration of Pu-Exos-PR better prevented the reduction in the number of TH-positive DA neurons. The Pu-Exos-PR(iv) group presented the greatest TH-positive areas and densities, with increased numbers of TH-positive cell bodies and fibers (Fig. 8E). The mice in the Pu-Exos-PR treatment group also showed significant improvements in motion in the field (Fig. 8F). Thus, Pu-Exos-PR are promising exosomes with excellent biocompatibility, effective doping of bioactive agents, and a unique ability to penetrate nasal tissues and the BBB, opening new avenues for the brain-targeted delivery of biomolecules for PD therapy [231].

Zhang et al. isolated PDNVs from *Lycium ruthenicum Murray* and reported that PDNVs attenuate Aβ-induced HT22 apoptosis by increasing the mitochondrial membrane potential, lowering the Bax/Bcl-2 ratio, decreasing cleaved caspase-3 expression, promoting Nrf2 nuclear translocation and upregulating HO-1 and NQO1 expression to attenuate oxidative stress, which has a protective effect and can be used as a potential dietary supplement for the prevention of Alzheimer’s disease [232]. Cai et al. found that bitter melon-derived PDNVs attenuated ischemia-reperfusion-induced BBB injury and inhibited neuronal apoptosis [233]. Brain inflammation, a hallmark of obesity, affects brain function through the gut-brain axis, and Sundaram et al. developed a garlic-derived PDNVs that preferentially uptake microglia and inhibit high-fat diet (HFD)-induced brain inflammation in obese mice, which were treated and showed improved memory function and increased glucose tolerance and insulin sensitivity [234].

In conclusion, the pathogenesis of brain diseases is complex and difficult to treat. PDNVs are widely used to treat brain diseases because of their high yield, excellent biocompatibility, non-immunogenicity, and ability to cross the BBB. However, research on the application of PDNVs for the treatment of brain diseases is still limited, and many challenges in terms of drug delivery efficiency and precise targeting remain. Future studies need to further optimize the design of PDNVs and explore their potential applications in different brain diseases.

### The Role of PDNVs in Osteoporosis

Osteoporosis (OP) is a systemic metabolic disease that is becoming increasingly prevalent and places a heavy burden on healthcare systems worldwide [235, 236]. Although standard treatments are often effective, they are often limited by side effects and the risk of developing drug resistance [237]. PDNVs play crucial roles in bone metabolism and have shown great potential in the treatment of osteoporosis.

**Fig. 9:**
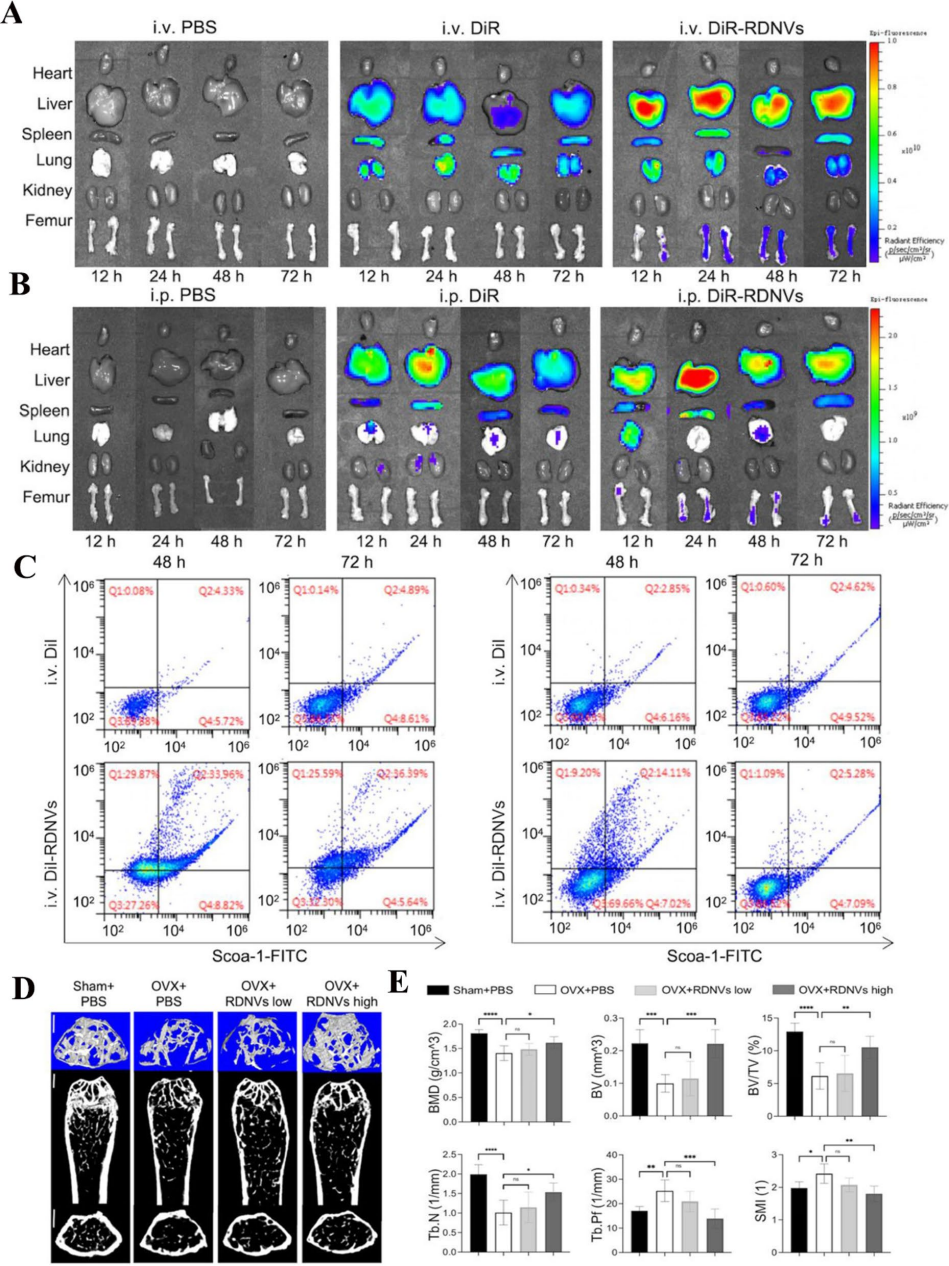
Biodistribution of RDNVs in vivo and RDNVs ameliorate ovariectomy-induced bone loss in vivo. **(A-B)** Fluorescence imaging in the heart, liver, spleen, lung, kidney and femur after i.v. and i.p. administration of PBS, DiR and DiR-labeled RDNVs in mice at different time points (12, 24, 48, and 72 h). (**C)** mBMSCs cellular uptake profiles in vivo were detected by FCM analysis after i.v. and i.p. administration of DiI and DiI-labeled RDNVs in mice at 12, 24, 48, and 72 h. (**D)** Mouse femurs were analyzed by micro-CT and the 3D reconstructions of trabecular were obtained using the software CTAn. Upper, three-dimensional reconstruction; Middle, sagittal; Bottom, transaxial (scale bar = 0.5 mm). (**E)** Histomorphometric analysis of femurs was determined by the scanner software. BMD, bone mineral density; BV, bone volume; BV/TV, bone volume density; Tb.N, trabecular number; Tb.Pf, trabecular pattern factor; SMI, structural model index Reprinted with permission from Ref [239]. Copyright Elsevier

OP is characterized by reduced bone mass and decreased bone density. Although existing drugs can inhibit bone resorption or promote bone formation, long-term use may cause side effects. Research has found that natural products PDNVs contain components such as miRNAs and bioactive lipids, which have antioxidant, anti-inflammatory, and regenerative potential, and can be used as alternative therapies. In this context, Hwang et al. successfully isolated PDNVs from yam and found that their osteogenic activity exceeded that of yam’s primary constituents, diosgenin and diosgeninogen—compounds that were not detected in the PDNVs based on HPLC analysis. These findings suggest that the osteogenic effects of yam-derived PDNVs may be attributed to their intrinsic proteins and mRNAs. In an ovariectomized (OVX) mouse model of osteoporosis, PDNV treatment significantly enhanced bone regeneration, increased bone volume and mineral density, and reduced bone resorption [238]. Zhao et al. revealed the potential of Rhizoma Drynariae-derived PDNVs (RDNVs) in the treatment of postmenopausal osteoporosis (PMOP), which can target and accumulate in the femur. Both i.p. and i.v. injections are effective, but the fluorescence signal is weaker with i.p. administration. This indicates that RDNVs can stably enter the circulation and specifically accumulate in the femur, rather than acting as free DIR components (Fig. 9A-B). To validate the targeting of RDNVs to bone tissue, DiI-labeled RDNVs were i.v. and i.p. injected into female C57BL/6J mice. Femoral bone marrow mesenchymal stem cells (mBMSCs) were isolated at 48 and 72 h. The results showed that at 48 h, the positive rate of mBMSCs uptake of RDNVs was > 35%, confirming that they could be effectively internalized (Fig. 9C). Researchers evaluated the therapeutic efficacy of RDNVs for PMOP using an ovariectomized (OVX) mouse model, and intraperitoneal injection of RDNVs reduced liver/spleen clearance. Results showed that the OVX group exhibited typical bone loss characteristics, including reduced bone mineral density (BMD), bone volume (BV), BV/TV, and trabecular number (Tb.N) decreased, while trabecular pattern factor (Tb.Pf) and structural modeling index (SMI) increased. High-dose RDNVs treatment significantly reversed these changes, improved trabecular structure, and reversed osteoporosis (Fig. 9D-E). Taken together, RDNVs derived from osteoblasts showed exciting bone tissue-targeting activity and reversed osteoporosis [239]. Pueraria lobata has previously been recognized for its role in promoting osteoblast proliferation during bone regeneration, though the underlying mechanisms remain to be fully elucidated. In a recent study, Zhan et al. isolated PDNVs from Pueraria lobata and found that they promoted the differentiation and mineralization of human bone marrow mesenchymal stem cells (hBMSCs) in vitro and in OVX-induced osteoporotic rats. Mechanistic studies revealed that PDNVs reduced the abundance of harmful bacterial strains in the gut of osteoporotic rats by regulating the gut microbiota metabolite trimethylamine-N-oxide (TMAO), and promoted the differentiation and function of hBMSCs by enhancing autophagy [240]. Cao et al. successfully isolated PDNVs from Morinda officinalis and reported that they specifically targeted the femur and enhanced bone formation better than did alendronate. Proteomic and bioinformatic analyses demonstrated that PDNVs alleviate PMOP by promoting osteoclast proliferation through the MAPK pathway and have good biocompatibility [141].

Overall, the bioactive components of PDNVs—such as proteins, DNA, and microRNAs—play a critical role in the pathogenesis and osteogenic regulation of osteoporosis. These vesicles contribute to the mitigation of osteoporosis by stimulating osteoblast proliferation, enhancing autophagic activity, and modulating the gut microbiota. Together, these mechanisms offer a multifaceted and promising therapeutic approach for the effective treatment of osteoporosis.

### The role of PDNVs in antiviral

Treatment of Severe Acute Respiratory Syndrome Coronavirus 2 (SARS-CoV-2) infection and related Coronavirus Disease 2019 (COVID-19), which severely affects the respiratory system and multiple organs and tissues and can lead to death, remains particularly challenging, especially for patients with comorbidities [241, 242]. COVID-19 severe disease can trigger a cytokine storm leading to alveolar epithelial and vascular endothelial cell injury. In contrast, ginger-derived PDNVs encapsulating ally-miR396a-5p inhibited the expression of Nsp12 and Nsp13 genes of SARS-CoV-2 by targeting them with specific miRNAs, which in turn blocked the activation of the NF-κB pathway, reduced the production of inflammatory cytokines, and protected the alveolar epithelial cells from apoptosis, thus effectively inhibiting virus-induced inflammation in the lungs [162]. Kalarikkal et al. isolated PDNVs from ginger and pomelo, identifying several miRNAs, including osa/cme miR-530b-5p, which specifically targets the ribosomal frameshift site between ORF1a and ORF1b. This targeting likely interferes with ribosomal sliding, thereby inhibiting ORF1b translation and suppressing SARS-CoV-2 replication [243]. Additionally, MIR2911, a microRNA found in honeysuckle decoction, was reported to significantly inhibit viral replication and expedite seroconversion in SARS-CoV-2-infected patients [244].

In conclusion, PDNVs can inhibit SARS-CoV-2 replication and attenuate COVID-19-associated inflammation through multiple mechanisms, providing a potential new strategy for novel coronavirus therapy. In the future, PDNVs can be used as adjunctive therapies in combination with existing antiviral drugs and vaccines to exert synergistic effects and improve the overall therapeutic efficacy against COVID-19. In addition, their potential application in other viral infectious diseases can also be explored to provide a wider range of options for antiviral therapy.

### The role of PDNVs in other diseases

The pathogens of chronic infectious diseases are complex and diverse, and the current use of single therapeutic drugs is ineffective in suppressing multiple pathogens and may lead to side effects. However, plant-derived PDNVs (such as those derived from ginger) offer safety and multi-target advantages, specifically inhibiting the growth of Porphyromonas gingivalis. Studies have shown that the lipids and miRNAs they carry can reduce bacterial growth and inflammatory responses, and effectively alleviate P. gingivalis-induced bone loss in mouse models [245]. A high-fat, high-sugar diet (HFHS) can induce ovarian insufficiency and premature ovarian failure (POF). Cui et al. demonstrated for the first time that oral administration of cranberry-derived PDNVs attenuates POF in mice by modulating the intestinal microbiota and decreasing ovarian granulosa cell PANoptosis, which provides a new strategy for the treatment of POF [246]. In addition, PDNVs can also be used to treat kidney disease. Zhang et al. found that orally administered lemon-derived PDNVs can be transported from the intestine to the kidneys, where they regulate calcium oxalate (CaOx) crystallization isoforms, inhibit CaOx-induced endoplasmic reticulum stress in renal tubular cells, restore subcellular function, and indirectly inhibit kidney stone formation [24]. In a separate study, the impact of lemon- and tomato-derived PDNVs on the chondrogenic differentiation of human adipose-derived stem cells (hASCs) was investigated. The results revealed that tomato-derived PDNVs promoted chondrogenesis and enhanced the expression of cartilage-specific markers, while lemon-derived PDNVs exerted an inhibitory effect on this differentiation process [54].

PDNVs isolated from plants have been shown to have various activities; however, PDNVs from coarse grains and legume cereals have rarely been reported. Pang et al. reported that PDNVs from kidney bean sources could provide significant improvement in high-fat diet-induced obesity through modulation of the gut microbiota and increase in the production of short-chain fatty acids (SCFAs) [132]. In addition to their therapeutic efficacy in treating diseases, PDNVs can also mitigate the adverse effects of drugs. Ye et al. demonstrated that PDNVs derived from bitter gourd alleviate the cardiotoxicity of doxorubicin by activating the p62/Keap1/Nrf2 pathway, without affecting its anticancer efficacy [247]. Huang et al. used transcriptomic analysis to show that PDNVs derived from tomatoes increase intestinal zinc transport by downregulating the expression of the metallothionein family. This provides new insights for the development of functional food supplements [248].

These findings suggest that PDNVs from different plant sources have effect differences, and the sources need to be selected according to the type of disease. Moreover, in addition to their therapeutic effects, PDNVs have anti-infective, organ-protective, and function-regulatory functions. Currently, there are fewer studies on PDNVs from coarse grains and legumes, which may be potentially valuable for chronic metabolic diseases (e.g., diabetes).

In conclusion, PDNVs from diverse plant origins may exert their biological effects through distinct mechanisms, contributing to both the development and treatment of various diseases. Their pharmacological activity often reflects, and in some cases exceeds, that of the whole plant or isolated compounds, offering a comprehensive therapeutic advantage. Due to their high biocompatibility and absence of ethical concerns, PDNVs can be administered directly to target tissues or delivered systemically via intravenous injection. As research advances, PDNVs are poised to become versatile and impactful agents in the development of novel therapeutic strategies.

**Table 5:** Application of PDNVs in different diseases

Biological activity	Disease	Plant source	Mechanism of Action	Ref.
Anti-inflammatory	Ulcerative colitis (UC)	Turmeric	TNVs alleviate UC multifacetedly by repairing the intestinal barrier, regulating intestinal flora, remodeling macrophage polarization and direct anti-inflammatory effects.	[166]
	Inflammatory bowel disease (IBD)	Ginseng	PDNVs ameliorate IBD by inhibiting activation of the NF-kB signaling pathway and increasing polarization of M2-like macrophages.	[206]
	UC	Ginger	EVs@ZIF-8@siRNA effectively inhibit targeted TNF-α.	[206]
	Neuroinflammation	Atractylodes lancea	PDNVs prevent LPS-stimulated pro-inflammatory effects by significantly reducing nitric oxide levels.	[207]
	Colitis	Cabbage	Reduces infiltration of neutrophils and other inflammatory cells in the colonic mucosa, as well as reducing pro-inflammatory cytokines in the colonic tissue.	[208]
	Chronic inflammation	Onion	Inhibition of IL-6 and IL-1β secretion and expression and NF-κB activation.	[90]
Anti-tumor	Triple-negative breast cancer (TNBC)	Brucea javanica	PDNVs are anti-tumor via inhibition of the PI3K/Akt/mTOR signaling pathway and inhibition of angiogenesis.	[210]
	TNBC	Platycodon grandiflorum	PDNVs inhibit tumor growth by reversing the immunosuppressive tumor microenvironment and modulating the gut microbiota.	[211]
	MDA-MB-231 cancer cells	Perilla leaves	PDNVs selectively inhibit the proliferation, migration, and invasion of MDA-MB-231 cells with high expression of caveolin-1.	[212]
	Hepatoma cells	Radix Polygoni Multiflori	PDNVs regulate the expression levels of numerous genes associated with cancer and the cell cycle.	[214]
	Melanoma	Hypericum perforatum	PDNVs are capable of generating reactive oxygen species (ROS) via type I and type II pathways in the presence of light.	[215]
	4T1 and HCC-1806 cells	Citrus limonL	PDNVs inhibit cancer cell proliferation through PI3K/AKT and MAPK/ERK signaling pathways.	[216]
	Lung cancer	Celery	/	[217]
	Breast and colon cancer	Ginseng	PDNVs reprogrammed M2-type macrophages to M1-type by activating the TLR4/MyD88 signaling pathway and increased secretion of the chemokines CCL5 and CXCL9.	[218]
	Breast cancer	Tea flowers	PDNVs inhibit tumor growth by inducing oxidative stress and modulating gut microbiota, among others.	[168]
Regeneration and wound healing	Spinal cord injury	Lycium barbarum L	PDNVs suppress inflammation by modulating microglial cell polarization, inhibiting oxidative stress, and modulating the PI3K/AKT signaling pathway.	[220]
	HDF	Peruviana fruits	/	[221]
	HDF, HaCaT and HUVEC	Wheat	PDNVs accelerate wound healing and tissue regeneration by promoting cell proliferation, migration, angiogenesis, and collagen synthesis.	[91]
	Wound healing	Dandelion	TH-GM dressing accelerates the healing process of invasive wounds through multiple mechanisms such as neutralizing exotoxins, promoting cell proliferation and migration, reducing inflammatory response, and promoting collagen maturation.	[222]
	Wound healing	Opuntia ficus-indica fruit	PDNVs accelerate the wound healing process through multiple mechanisms such as antioxidant, anti-inflammatory, promotion of cell migration and angiogenesis.	[223]
	Wound healing	Mint leaves	MENV-HG effectively inhibits bacterial growth and reduces infection by disrupting bacterial cell walls and releasing bioactive components.	[224]
Liver diseases	Liver damage	Ginger	Ginger-derived PDNVs prevent alcohol-induced liver injury by activating the Nrf2 signaling pathway and inhibiting ROS production.	[75]
	Acute liver injury	Shiitake mushroom	PDNVs attenuate acute liver injury by inhibiting NLRP3 inflammasome activation.	[202]
	Liver injury	Pomegranate	PDNVs alleviate alcohol-induced leaky gut and liver injury by reducing oxidative stress and inflammatory responses.	[226]
	Non-alcoholic fatty liver disease	Hemp sprout	PDNVs alleviate NAFLD-induced leaky gut and hepatic fibrosis by inhibiting oxidative stress and reducing fibrosis marker protein expression.	[227]
	Acute liver failure (ALF)	Garlic	PDNVs alleviate acute liver failure by suppressing the inflammatory response, activating autophagy, and inhibiting activation of the NLRP3 inflammasome.	[81]
Brain diseases	Parkinson’s disease (PD)	Pueraria lobata	PDNVs ameliorate PD by improving mitochondrial dysfunction and regulating gene expression.	[231]
	Aβ-induced apoptosis and oxidative stress in HT22 cells.	Lycium ruthenicum Murray	PDNVs exert a protective effect against HT22 cell injury by inhibiting Aβ-induced apoptosis and attenuating Aβ-induced oxidative stress.	[232]
	Ischemic brain injury	Momordica charantia	PDNVs exert neuroprotective effects by down-regulating MMP-9 and up-regulating the expression of ZO-1 and claudin-5, as well as activating the AKT/GSK3β signaling pathway.	[233]
Osteoporosis	Osteoporosis	Yam	PDNVs promote bone regeneration by activating the BMP-2/p-p38-dependent Runx2 signaling pathway.	[238]
	Postmenopausal Osteoporosis	Rhizoma Drynariae	PDNVs promote osteogenic differentiation by targeting the ERα signaling pathway and upregulating BMP2 and RUNX2 expression.	[239]
	Osteoporosis	Pueraria lobata	PDNVs attenuate osteoporosis by promoting differentiation and mineralization of hBMSCs, modulating the gut microbiota and enhancing autophagy.	[240]
	Postmenopausal Osteoporosis	Morinda Officinalis	PDNVs alleviate PMOP by promoting osteoblast proliferation through the MAPK pathway.	[141]
Antiviral	COVID-19	Ginger	PDNVs effectively suppressed virus-induced lung inflammation by targeting the Nsp12 and Nsp13 genes of SARS-CoV-2 with specific miRNAs and inhibiting their expression.	[162]
Other diseases	Antimicrobial	Ginger	PDNVs exert their antimicrobial effects through, among other things, selective uptake of membrane interactions with P. gingivalis.	[245]
	Premature ovarian failure (POF)	Cranberry	PDNVs attenuate premature ovarian failure in mice through various mechanisms including modulation of gut microbiota and reduction of PANoptosis in ovarian granulosa cells.	[246]
	Kidney stones	Lemon	PDNVs inhibit kidney stone progression by altering CaOx crystallization isoforms and inhibiting CaOx-induced endoplasmic reticulum stress response in tubular cells.	[24]
	Obesity	Kidney bean	PDNVs ameliorate obesity by modulating the production of gut microbes and SCFAs.	[132]

## PDNVs as nanocarriers for therapeutic agents

In the field of nanotechnology, designing smart carriers for targeted drug delivery is the gold standard, and PDNVs may be ideal for clinical translation [249]. From a drug delivery perspective, liposomes are among the leading drug delivery platforms, and PDNVs are similar to liposomes in that they are phospholipid structured, delivering both hydrophilic and hydrophobic cargo [250], PDNVs themselves carry bioactive molecules, such as metabolites, proteins, lipids, and nucleic acids, making them a small but powerful carriers [251]. Moreover, compared with other artificial carriers, PDNVs have lower immunogenicity and toxicity, as well as good circulatory stability, and can cross the BBB. Compared with more popular MEVs [252], in addition to the above advantages, PDNVs also have greater potential for application because of their lower cost, lower time consumption, and ability to be acquired in large quantities in a short period of time.

Wang et al. reported that when nanoparticles made from grapefruit-derived PDNVs and synthetic lipids were injected into mice, liposomes caused liver damage, that PDNVs did not pass the placental barrier when injected intravenously into pregnant mice, and that the internalization rate of PDNVs was more than 80% in subsequent assays, whereas the rate of internalization of liposomes was only about 40% [80]. In Zhang et al. it was noted that ginger-derived PDNVs exhibited better pH-dependent drug release properties than commercially available liposomal DOX [175]. PDNVs can also be biased toward tumors by targeting tissues through a specific endocytosis mechanism, while reducing off-target effects [195]. These attributes position PDNVs as advantageous over conventional liposomes or synthetic drug carriers, establishing a robust foundation for the development of next-generation delivery systems.

### Delivery of genetic drugs

PDNVs are natural nanocarriers that can effectively deliver plant miRNAs to mammalian cells, cross-species regulate gene expression, increase miRNA diversity and exert multi-target effects, which are important for diseases [253]. miR17 can target multiple genes and regulate a variety of biological processes. Its multi-targeting properties give it a unique advantage in cancer therapy, as it can affect multiple tumor-related pathways at the same time. Using grapefruit-derived PDNVs to deliver therapeutic miR17 to mouse brain tumors via intranasal administration, Zhuang et al. were able to significantly inhibit tumor growth and prolong survival time in mice. It provides a non-invasive method for the treatment of brain diseases [185]. miR-184 is a representative miRNA drug that is involved in a variety of biological and pathological processes, including proliferation, apoptosis, and tumorigenesis. cabex-sourced PDNVs encapsulated miR-184 with minimal effect on its potential and morphology, and efficiently delivered miR-184 to colon cancer cells, resulting in an increase in cellular levels of the miR of 246, 000-fold [109]. Increased expression of CD98 in the colon plays an important role in colitis and colitis-related cancers. Thus Zhang et al. established and characterized natural nanoparticles with specific drug carriers, loaded ginger-derived PDNVs with siRNA against CD98 (siRNA-CD98), and investigated that these siRNA-loaded “natural liposomes” could effectively and specifically target the colon [153]. Piwi-interacting RNAs (piRNAs) are a class of small RNAs, and oral delivery of an anti-agomir (anta-HAAPIR) targeting cardiac apoptosis-associated piRNAs (HAAPIRs) results in vascular remodeling, thereby attenuating the onset and progression of aortic dissection. Liu et al. encapsulated an anti-agomir into green tea-derived PDNVs by electroporation to form an anti-agomir-PDNVs complex, which was able to target aortic lesions, reduce HAAPIR expression, and decrease vasodilation. In addition, a smaller vessel width and higher survival rate were observed in aortic dissection model mice [254]. In the study of Yan et al. PDNVs were first isolated from Brucea javanica and found that their PDNVs delivered 10 functional miRNAs to 4T1 cells, which significantly delayed the growth and metastasis of 4T1 cells by regulating the PI3K/Akt/mTOR signaling pathway and promoting ROS/caspase-mediated apoptosis [210].

Overall, miRNAs represent valuable disease biomarkers and therapeutic targets due to their ability to regulate complex biological pathways. As such, they hold significant potential for advancement in biopharmaceutical research and drug development. However, free miRNAs are inherently unstable and prone to rapid degradation. In contrast, when encapsulated within PDNVs, miRNAs exhibit enhanced stability and bioavailability. PDNVs serve as effective carriers, protecting miRNAs from enzymatic degradation in the gastrointestinal tract and other physiological environments. Therefore, leveraging PDNVs as specialized delivery vehicles for miRNA-based therapeutics presents a highly promising strategy.

### PDNVs modifications

Similar to MEVs, PDNVs facilitate intercellular communication by selectively targeting recipient cells and delivering bioactive molecules. However, their inherent therapeutic effects are limited by suboptimal targeting capabilities. To overcome these limitations, engineering strategies such as surface functionalization and membrane coating have been employed to significantly improve their therapeutic efficacy and targeting precision [255].

Biological modification is the most widely used membrane modification strategy [256], Improving the biocompatibility of the delivery system, achieving precise targeting, and reducing off-target effects are essential. Folic acid (FA)-modified nanoparticles have been reported to specifically target and concentrate within M1 macrophages, a key cellular component of the inflammatory process associated with rheumatoid arthritis (RA) [257]. Taking advantage of this targeting ability, Han et al. integrated FA on the surface of ginger-derived PDNVs (GDEVs) and developed FA-GDEV, which retains its intrinsic immunomodulatory properties, by inserting FA-PEG2000-cholesterol termini into the lipid bilayer membranes of GDEV. FA-GDEV selectively targets M1 macrophages in inflamed joints via the folate receptor (FR). FA-GDEV has been shown to target M1-type macrophages in vitro and in vivo and can be taken up by M1-type macrophages through FR-mediated endocytosis and accumulate in inflamed joints in mice [258]. In another report, which utilized cRGD as a targeting ligand that can have high affinity for integrin avb3, which is overexpressed in ovarian cancer cells, heparin-cRGD (HR) was first prepared by attaching the amino group of cRGD to the carboxyl group of heparin, and then DN was prepared by coupling HR as a carrier with pH-sensitive adipic acid diamide-DOX (ADH-DOX). Because the orange-derived PDNVs (OEV) have abundant cell membrane proteins on their surface, the reactive amino group of OEV readily reacts with the additional carboxyl group of heparin in DN. This repair strategy significantly improved the loading efficiency of DOX and cRGD in OEV. In the final study, OEV as a carrier efficiently delivered DN into ovarian cancer cells, resulting in high accumulation and permeability in tumor tissues [259]. In another study, the same approach was used to patch DOX-loaded heparin-based nanoparticles with natural grapefruit-derived PDNVs for glioma treatment, with substantial accumulation in tumor tissue [189]. Guo et al. reported that EDC and NHS can activate the NH_2_ group in DSPE-PEG2000-NH_2_ micelles at 4 °C, enabling the conjugation of anti-DR5 antibodies to form DSPE-PEG2000-DR5. This conjugate was then incorporated into the membranes of PDNVs through lipid insertion under continuous stirring at 4 °C overnight [260]. Similarly, Moon et al. demonstrated that incorporating a functionalized lipid component containing a maleimide group into grapefruit-derived PDNV membranes allowed for specific targeting of hCMEC/D3 cells. These findings suggest that targeted delivery can be effectively enhanced through the application of click chemistry to attach aptamers to the PDNV surface [70].

Although membrane surface modification has provided unique properties to traditional PDNVs, it is limited by the single function of the ligand and complicated preparation methods. In contrast, the development of biomimetic nanocarriers utilizing cellular components derived from autologous biological sources is anticipated to produce PDNVs with reduced immunogenicity, prolonged circulation time, and enhanced targeting capability [255]. RA is a chronic, treatment-resistant autoimmune disorder for which effective therapeutic options remain limited. Whereas the roots of Morinda officinalis possess significant anti-inflammatory activity, the production and anti-inflammatory activity of PDNVs from its roots are enhanced by ultraviolet (UV) pretreatment followed by extrusion to encapsulate erythrocyte membranes on the surface of the PDNVs to minimize their immunogenicity and prolong their circulation time. In an adjuvant-induced arthritis mouse model, PDNVs encapsulated with erythrocyte membranes presented significantly increased accumulation at the joint site and longer retention times than PDNVs did, suggesting that erythrocyte membrane artifacts enhance the passive targeting ability of PDNVs [261].

Overall, miRNAs play an important role in disease treatment due to their multiple targets. And PDNVs themselves carry miRNAs and play a better therapeutic effect in a variety of diseases. Even so, in some diseases, some specific miRNAs are not in PDNVs, and miRNAs are very easy to be degraded, so they are encapsulated into PDNVs to play a therapeutic effect. However, PDNV also has some inherent drawbacks, such as lack of targeting, so strategies to engineer PDNV, such as modifying the surface of PDNV, have been proposed. Most of them are still inserting some targets into the membranes of PDNVs by means of lipid insertion. Although the modification of the membrane surface confers properties that conventional PDNVs do not originally possess, they are still limited by the complexity of the preparation method. Subsequently, the concept of biomimetic was proposed to encapsulate the cell membrane on the surface of PDNVs, so that the PDNVs have lower immunogenicity, longer circulation time and stronger targeting ability. Thus engineering PDNVs for modification has broad application prospects and the results are encouraging.

## Clinical trials

Clinical trials for some of the PDNVs have now been proposed, thus demonstrating the potential of PDNVs for clinical applications (Table 6). A study to evaluate the effects of ginger-derived PDNVs or curcumin alone or in combination with curcumin on symptoms and disease scores in patients with refractory inflammatory bowel disease (IBD). A number of patients with chronic IBD were recruited and randomized into 3 groups and were treated orally once daily for a total of 28 days. As described in the Primary Outcome Measures, there was a reduction in inflammatory cells in biopsies after treatment compared with before treatment. The secondary outcome measures included a questionnaire reporting subjective symptom reduction. The results of their study have not yet been submitted (ClinicalTrials.gov: NCT04879810). The ability of grape-derived PDNVs to prevent chemotherapy-associated oral mucositis in head and neck cancer patients will be explored in another study. In addition, the effects of PDNVs on cytokine production, immune responses to tumor exosomal antigens, metabolism, and molecular markers in these patients will be evaluated. Sixty subjects received oral PDNVs daily for a total of 35 days during chemotherapy. In the primary outcome measures described, pain levels for oral mucositis were assessed weekly during treatment (6–7 weeks) and for six months after the end of treatment, which lasted about 30 days. In secondary outcome measures, the levels of immune biomarkers (cytokines, T cells, NK cells, CD11c, and IL12) in the blood at the end of radiation therapy are compared to baseline levels. The levels of immune biomarkers (CD3, CD8, CD11b, F4/80, and BRDU) in mucosal tissue scraped at the end of radiation therapy were compared to baseline levels. The results of the study have not yet been submitted and this study phase is in Phase 1(ClinicalTrials.gov: NCT01668849). And another study plans to address curcumin delivery by using plant-derived PDNVs to deliver the drug to colon tumors and normal colon tissue. Still recruiting, 35 subjects are expected to be recruited, and the experiment is divided into 3 groups, an oral curcumin alone group (15 subjects taking 3.5 g of tablets orally for 7 days), a PDNVs-conjugated curcumin group (15 subjects taking tablets daily for 7 days), and a no-treatment group(ClinicalTrials.gov: NCT01294072). Unfortunately, a preliminary clinical trial on the ability of ginger- or aloe-derived PDNVs to attenuate insulin resistance and chronic inflammation in patients with polycystic ovary syndrome (PCOS) was withdrawn because the researchers left the university before the project was approved(ClinicalTrials.gov: NCT03493984) [28].

In conclusion, after searching for applications of PDNVs in clinical studies, we found only 4, including 1 withdrawn, whose findings were not published on ClinicalTrials.gov. This indicates the scarcity and challenge of PDNVs in clinical application, and the standardization of the production and quality control of PDNVs is one of the challenges; thus, it is necessary to meet these challenges and conduct more clinical trials to promote the future application of PDNVs in clinical treatment.

**Table 6:** The clinical state of PDNVs studies

Trail ID	Official Title	PDNVs source	Targeted disease	Cargo	Functions	Recruitment status
NCT04879810	Pilot Clinical Trial Investigating the Ability of Plant Exosomes +/- Curcumin to Abrogate Symptoms of Inflammatory Bowel Disease (IBD)	Ginger	Inflammable bowel disease	Curcumin	Anti-inflammatory effects	Completed
NCT01668849	Preliminary Clinical Trial Investigating the Ability of Plant Exosomes to Abrogate Oral Mucositis Induced by Combined Chemotherapy and Radiation in Head and Neck Cancer Patients	Grape	Head and Neck Cancer, Oral Mucositis	/	Reducing the incidence of oral mucositis during radiation and chemotherapy treatment for head and neck tumors	Completed
NCT01294072	Phase I Clinical Trial Investigating the Ability of Plant Exosomes to Deliver Curcumin to Normal and Malignant Colon Tissue	Plant	Colon Cancer	Curcumin	Delivery of curcumin to colon tumors and normal colon tissue.	Recruiting
NCT03493984	A Preliminary Clinical Trial Investigating the Ability of Plant Exosomes to Mitigate Insulin Resistance and Chronic Inflammation in Patients Diagnosed With Polycystic Ovary Syndrome (PCOS)	Ginger or Aloe vera	Polycystic Ovary Syndrome	/	Mitigating Insulin Resistance, Anti- Inflammation	Withdrawn

## Challenges in PDNVs Applications

Compared to the isolation and extraction of MEVs, the process for isolating and purifying PDNVs is more complex and requires pre-treatment of plant materials. Factors such as the plant species, specific plant parts used (e.g., flowers, leaves, fruits, seeds, roots, stems), geographical origin, and extraction techniques can significantly influence the yield and purity of PDNVs. To ensure consistency, it is crucial to use the same plant origin and plant part during extraction, and to optimize isolation protocols to achieve the desired concentration and purity for research applications. For potential clinical applications, it is essential to standardize PDNV production and ensure compliance with Good Manufacturing Practices (GMP). This includes establishing rigorous quality control checkpoints throughout the extraction process to enable the scalable production of high-purity PDNVs. Additionally, post-isolation identification protocols must be standardized. At present, there is no universally accepted method for identifying surface proteins on PDNVs, likely due to plant-derived heterogeneity. Therefore, further investigation is required to determine whether PDNVs from different plant sources share common protein markers. This will facilitate the development of standardized identification methods and contribute to improving PDNV purity and consistency.

Fewer studies have been conducted on the storage conditions of PDNVs, and their storage faces great challenges. Currently applied preservation techniques include freezing, lyophilization, and spray drying [262]. Lyophilization of ginseng-derived PDNVs has been reported to be stable for 60 days at room temperature [77]. In contrast, PDNVs are commonly preserved by freezing at − 80 °C. Some researchers have also stored PDNVs at − 80 °C, − 20 °C, 4 °C, and room temperature to study their stability and reported that they are more stable at − 80 °C and − 20 °C [174]. However, another study reported a significant increase in the cytotoxicity of PDNVs after storage at − 80 °C, but the exact reason for this increase is unclear [68]. For MEVs, preservation stability can be enhanced through the use of CPAs; however, no comparable studies have been reported for PDNVs. Consequently, dedicated research on the storage conditions of PDNVs is necessary to improve their stability and support their transport, thereby ensuring their functional integrity in subsequent applications.

PDNVs possess unique properties that make them promising candidates for the treatment of various diseases. Nonetheless, challenges remain, particularly regarding their targeting specificity and safety. Native PDNVs exhibit limited targeting capacity, prompting the development of engineering strategies to enhance their cellular uptake and targeting efficiency. However, the potential immunogenicity and toxicity associated with engineered PDNVs must be carefully evaluated to ensure their safe clinical application. Although many studies have demonstrated that PDNVs are biocompatible [263], in-depth studies on the potential effects of engineered PDNVs on the host are needed. Another factor affecting safety is the route of administration of PDNVs. Intravenous injection, in particular, is a commonly used method of injection by researchers because of its quicker and longer-lasting effects, but there is also a greater risk of adverse drug reactions. With respect to animal studies on PDNVs, the focus is more on the oral route, which is simpler and safer. In the present study, most of the routes of administration of PDNVs were safer, and the optimal route of administration of PDNVs needs to be determined according to the experimental purpose. Once the route of administration is determined, the safe and minimum effective doses of different PDNVs may also differ. Therefore, PDNVs need to be subjected to comprehensive quality control measures prior to administration to eliminate concerns about the biosafety and potential toxicity of unknown bioactive components [178].

## Conclusion and future perspective

PDNVs are considered to be important mediators between plants and other species, and also play a protective role during plant immunity and growth, and serve as mediators of cross-domain communication when interacting with cells of other species [25]. In contrast to mammalian extracellular vesicles, the biogenesis of PDNVs remains less well understood, likely due to the structural complexity of plant cell walls, which poses challenges for studying intercellular communication. Current evidence suggests that PDNV secretion is primarily associated with the plant’s immune response. Upon pathogen infection, MVBs, vesicles, and EXPO fuse with the plasma membrane, releasing vesicles into the extracellular space. These vesicles subsequently rupture and discharge defense compounds that help suppress pathogen proliferation. Compared with MEVs, the research on their PDNVs has just started and there is no consensus on the standardized nomenclature of PDNVs, but there have been many exciting discoveries that open up new avenues for the therapeutic application of nanomedical materials in diseases. To this end, we have also compared the advantages and disadvantages of different sources of extracellular vesicles through a table (Table 7).

PDNVs can be isolated using various techniques, including ultracentrifugation, density gradient centrifugation, ultrafiltration, size exclusion chromatography, immunoaffinity capture, polymer precipitation, and electrophoresis, often in combination. Despite these advancements, centrifugation—particularly ultracentrifugation and density gradient methods—remains the primary technique, although it is notably time-consuming. Compared with MEVs, since PDNVs are derived from plants, pre-treatment of plants is also required before isolation and purification, which involves crushing plant tissues by low-speed agitation to obtain plant juices or removing the extracellular fluid of phone-generated plants by tissue infiltration, which can maximize the integrity of the cells, has high purity but low concentration, and is also more applicable to leaf tissues. The sap obtained by the tissue crushing method, on the other hand, has difficulty obtaining high-purity PDNVs because it contains many plant fragments. In recent years, basic biological research on PDNVs has developed rapidly, but at present, the yield of PDNVs is affected by extraction technology, and it is not yet possible to extract many high-purity PDNVs. Moreover, in the process of isolation, the parameters of its isolation, the plant category, plant origin, and different parts of the plant may lead to different purities, particle sizes, and bioactive components of PDNVs. Accordingly, refining the methods for isolating and purifying PDNVs is necessary to improve both yield and purity, while also aiming to reduce processing time and associated costs. Due to the current gaps in scientific understanding, applying the nomenclature used for MEVs to plant-derived EVs remains problematic. A consistent classification framework cannot be developed without comprehensive physical characterization and a clearly defined biosynthetic origin [264]. To standardize the field of PDNVs, their biosynthetic pathways should be identified as soon as possible, and a more standard nomenclature should be established. The current morphological characterization of PDNVs revealed a cup-shaped structure via TEM, and the contents of PDNVs included lipids, proteins, nucleic acids, and other metabolites. However, due to the inherent heterogeneity among plant species, universal or specific markers for PDNVs have not yet been identified. This complicates the verification of whether isolated PDNVs are equivalent to EVs as traditionally defined. Consequently, it is important to investigate whether PDNVs derived from different plant sources share a common protein composition, in order to advance the standardization of their identification. Additionally, plant growth conditions—including climatic and seasonal variations—can significantly influence both the physiological state of the plant and the abundance of PDNVs [25]. Thus, we can shift our attention from fresh plants to dried or even decocted plants, shedding light on new sources of PDNVs. In addition, there are some controversies about the preservation of PDNV, and some studies have noted the toxicity of PDNV after preservation at − 80 °C; however, only a few reports on this topic have been published. Therefore, finding cryogenic freezing or prolonging the preservation time of PDNVs by finding high-quality cryoprotectors, as well as methods that have no effect on the stability and structure of PDNVs, are key to the efficient preservation of PDNVs.

Understanding the in vivo metabolism, systemic absorption, and biodistribution of PDNVs is essential. Different routes of administration significantly influence their biodistribution, yet the interactions between PDNVs as delivery vehicles and target receptor cells remain inadequately characterized. Given the complexity of PDNV internalization and distribution mechanisms, it is imperative to investigate their internalization pathways as well as their physiological and biochemical properties to advance their therapeutic application. Therefore, to address this issue, the internalization mechanism and physiological and biochemical properties of PDNVs should be studied. PDNVs derived from plants can be used not only as therapeutic agents but also as drug delivery vehicles. In particular, PDNVs derived from Chinese herbs may carry bioactive components of Chinese medicines with biological effects, such as antitumor, anti-inflammatory, antioxidant, and anti-osteoporosis effects, and their unique properties make them effective tools for the treatment of a variety of diseases. However, the direct use of PDNVs usually involves only simple extraction and lacks specialized drug design and optimization, so there is still room for improvement in terms of targeting performance and therapeutic efficacy [11]. To overcome these potential shortcomings, researchers are now beginning to address the challenges faced by conventional drug delivery systems by engineering strategies to improve the targeting performance of PDNVs to specific tissues and organs. The means to do so can be engineered through modifications on the surface of PDNVs and fusion techniques, among others, while also paving the way for encapsulation of therapeutic molecules such as chemotherapeutic drugs and nucleic acids. In conclusion optimizing the efficacy of PDNVs in drug delivery systems plays a crucial role. However, no relevant studies have reported whether PDNVs still maintain their original biological activity, whether the bioactive molecules of PDNVs are changed, whether the whole nanocarrier is still safe and non-toxic, and whether the encapsulated therapeutic molecules, PDNVs, themselves carry out interactions with each other among the active components. Therefore, before PDNVs are engineered, the contents of their PDNVs can be removed to ensure that they do not interact with the encapsulated drug molecules or modified targets. However, after the contents are removed, the morphology, size, and activity of the PDNVs should be verified again to avoid affecting the original properties of the PDNVs [28]. The assessment of their activity is also recommended for addition to the subsequent quality control system. In view of the above, PDNVs hold great promise as drug delivery vehicles, but a great deal of effort is needed to move from the laboratory to clinical practice, especially given the critical importance of comprehensively evaluating their safety, pharmacokinetics and immunogenicity; establishing standardized production protocols; and controlling their quality from the production stage to the preclinical stage, which are essential for reproducibility and consistency during clinical application [265].

In conclusion, PDNVs have a promising future in nanomedicine. Owing to their superior biocompatibility, low immunogenicity, intrinsic bioactivity, and easy uptake by receptor cells, natural and endogenous PDNVs surpass synthetic nanoparticles and EVs of mammalian origin. Their lipid bilayer structure enhances the stability of the drug and prevents degradation due to environmental factors. In addition, PDNVs contain many pharmacologically significant bioactive components that can facilitate cross-domain communication and serve as therapeutic agents or drug delivery carriers. Modifying PDNVs to specifically target certain tissues and organs, along with enabling the encapsulation of various therapeutic agents, significantly enhances their potential as advanced drug delivery systems. While PDNVs offer numerous biomedical advantages, they also face substantial challenges. These include the need for standardized protocols in their production, identification, and characterization; the absence of an agreed-upon nomenclature; limited knowledge on long-term storage; unclear mechanisms of in vivo metabolism and distribution; and the complexities involved in their bioengineering and clinical application. A particularly pressing issue is the establishment of unified industry standards to ensure consistent quality control. Additionally, thorough preclinical studies are essential to validate the dosage, safety, and efficacy of PDNV-based therapies. Thus, there is still a long way to go to move PDNVs from the laboratory to clinical research, to collaborate in interdisciplinary research against all odds, and to develop PDNVs to the best of their ability to meet clinical needs and expand their therapeutic applications, and to open a new chapter in the treatment of human diseases.

**Table 7:** Comparing the advantages and disadvantages of EVs from different sources in the field of medical applications

Source of EVs	Advantages	Disadvantages	Ref.
FEVs	Antifungal resistance; Stimulating immune responses; Mediating biofilm formation; As vaccine candidates;	Pathogenicity; The stoichiometry of FEVs release remains elusive; Does not reflect whether FEVs is continuously secreted; Impure; Few markers	[266, 267]
BEVs	Non-replicative nature; Easy to modify; Physiological functions; Versatility	Toxicity; Batch-to-batch variations; Limited scalability; Poor targeting ability; Inherent biological variabilities	[268, 269]
MEVs	Ideal biocompatibility; Crossing the blood-brain barrier; Noninvasive biomarke	Insufficient targeting; Low yield; High cost; High heterogeneity	[7, 31, 270, 271]
PDNVs	Low immunogenicity; Eexcellent biocompatibility; High productivity; Free of human pathogens; Environmentally sustainable sources of raw materials	High heterogeneity; Lack of standardized isolation Methods; Seasonal harvest	[25, 272]

FEVs: fungal extracellular vesicles; BEVs: bacterial extracellular vesicles

## Data Availability

No datasets were generated or analysed during the current study.

## References

[1] Lee JC, Ray RM, Scott TA. Prospects and challenges of tissue-derived extracellular vesicles. Mol therapy: J Am Soc Gene Therapy. 2024;32:2950–78. 10.1016/j.ymthe.2024.06.025 PMC11403234 38910325

[2] Cheng X, Henick BS, Cheng K. Anticancer Therapy Targeting Cancer-Derived Extracellular Vesicles. ACS Nano. 2024;18:6748–65. 38393984 10.1021/acsnano.3c06462

[3] van Niel G, D’Angelo G, Raposo G. Shedding light on the cell biology of extracellular vesicles. Nat Rev Mol Cell Biol. 2018;19:213–28. 29339798 10.1038/nrm.2017.125

[4] Xia Y, Zhang J, Liu G, Wolfram J. Immunogenicity of Extracellular Vesicles, Advanced materials (Deerfield Beach, Fla.), 36 (2024) e2403199. 10.1002/adma.202403199 38932653

[5] Kumar MA, Baba SK, Sadida HQ, Marzooqi SA, Jerobin J, Altemani FH, Algehainy N, Alanazi MA, Abou-Samra AB, Kumar R, Al-Shabeeb AS, Akil MA, Macha R, Mir AA, Bhat. Extracellular vesicles as tools and targets in therapy for diseases. Signal Transduct Target Ther. 2024;9:27. 38311623 10.1038/s41392-024-01735-1 PMC10838959

[6] Wen CN, Ma H, Xu LM, Gu ZD, Li H, Zhang YM, Liang YJ, Xu X. Recent advances in the clinical application of exosomes for disease diagnosis and therapeutic strategies. Int J Surg. 2025;111:4609–28. 40387699 10.1097/JS9.0000000000002518

[7] Du S, Guan Y, Xie A, Yan Z, Gao S, Li W, Rao L, Chen X, Chen T. Extracellular vesicles: a rising star for therapeutics and drug delivery. J Nanobiotechnol. 2023;21:231. 10.1186/s12951-023-01973-5 PMC10360328 37475025

[8] Yang C, Xue Y, Duan Y, Mao C, Wan M. Extracellular vesicles and their engineering strategies, delivery systems, and biomedical applications. J Control Release. 2024;365:1089–123. 38065416 10.1016/j.jconrel.2023.11.057

[9] Yi Q, Xu Z, Thakur A, Zhang K, Liang Q, Liu Y, Yan Y. Current understanding of plant-derived exosome-like nanoparticles in regulating the inflammatory response and immune system microenvironment. Pharmacol Res. 2023;190:106733. 36931541 10.1016/j.phrs.2023.106733

[10] Wang J, Jing J, Zhou C, Fan Y. Emerging roles of exosomes in oral diseases progression. Int J Oral Sci. 2024;16:4. 38221571 10.1038/s41368-023-00274-9 PMC10788352

[11] Wang X, Xin C, Zhou Y, Sun T. Plant-Derived Vesicle-like Nanoparticles: The Next-Generation Drug Delivery Nanoplatforms. Pharmaceutics; 2024. p. 16. 10.3390/pharmaceutics16050588 PMC11125130 38794248

[12] Fang Z, Liu K. Plant-derived extracellular vesicles as oral drug delivery carriers. J Control Release. 2022;350:389–400. 36037973 10.1016/j.jconrel.2022.08.046

[13] Cai Y, Zhang L, Zhang Y, Lu R. Plant-Derived Exosomes as a Drug-Delivery Approach for the Treatment of Inflammatory Bowel Disease and Colitis-Associated Cancer, Pharmaceutics, 14 (2022). 10.3390/pharmaceutics14040822 PMC9029273 35456656

[14] Kim M, Jang H, Kim W, Kim D, Park JH. Therapeutic Applications of Plant-Derived Extracellular Vesicles as Antioxidants for Oxidative Stress-Related Diseases, Antioxidants (Basel, Switzerland), 12 (2023). 10.3390/antiox12061286 PMC10295733 37372016

[15] Tan ZL, Li JF, Luo HM, Liu YY, Jin Y. Plant extracellular vesicles: A novel bioactive nanoparticle for tumor therapy. Front Pharmacol. 2022;13:1006299. 36249740 10.3389/fphar.2022.1006299 PMC9559701

[16] Halperin W, Jensen WA. Ultrastructural changes during growth and embryogenesis in carrot cell cultures. J Ultrastruct Res. 1967;18:428–43. 6025110 10.1016/s0022-5320(67)80128-x

[17] An Q, Huckelhoven R, Kogel KH, van Bel AJ. Multivesicular bodies participate in a cell wall-associated defence response in barley leaves attacked by the pathogenic powdery mildew fungus. Cell Microbiol. 2006;8:1009–19. 16681841 10.1111/j.1462-5822.2006.00683.x

[18] An Q, Ehlers K, Kogel KH, van Bel AJ, Huckelhoven R. Multivesicular compartments proliferate in susceptible and resistant MLA12-barley leaves in response to infection by the biotrophic powdery mildew fungus. New Phytol. 2006;172:563–76. 17083686 10.1111/j.1469-8137.2006.01844.x

[19] Regente M, Corti-Monzon G, Maldonado AM, Pinedo M, Jorrin J, de la Canal L. Vesicular fractions of sunflower apoplastic fluids are associated with potential exosome marker proteins. FEBS Lett. 2009;583:3363–6. 19796642 10.1016/j.febslet.2009.09.041

[20] Zhu H, He W. Ginger: a representative material of herb-derived exosome-like nanoparticles. Front Nutr. 2023;10:1223349. 37521414 10.3389/fnut.2023.1223349 PMC10374224

[21] Pozo-Acebo LD, López de Las Hazas MC, Tomé-Carneiro J, Del Saz-Lara A, Gil-Zamorano J, Balaguer L, Chapado LA, Busto R, Visioli F, Dávalos A. Therapeutic potential of broccoli-derived extracellular vesicles as nanocarriers of exogenous miRNAs. Pharmacol Res. 2022;185:106472. 36182038 10.1016/j.phrs.2022.106472

[22] Wang B, Guo X-J, Cai H, Zhu Y-H, Huang L-Y, Wang W, Luo L, Qi S-H. Momordica charantia-derived extracellular vesicles-like nanovesicles inhibited glioma proliferation, migration, and invasion by regulating the PI3K/AKT signaling pathway. J Funct Foods. 2022;90:104968.

[23] Qiao Z, Zhang K, Liu J, Cheng D, Yu B, Zhao N, Xu FJ. Biomimetic electrodynamic nanoparticles comprising ginger-derived extracellular vesicles for synergistic anti-infective therapy. Nat Commun. 2022;13:7164. 36418895 10.1038/s41467-022-34883-5 PMC9684156

[24] Zhang L, Li S, Cong M, Liu Z, Dong Z, Zhao M, Gao K, Hu L, Qiao H. Lemon-Derived Extracellular Vesicle-like Nanoparticles Block the Progression of Kidney Stones by Antagonizing Endoplasmic Reticulum Stress in Renal Tubular Cells. Nano Lett. 2023;23:1555–63. 36727669 10.1021/acs.nanolett.2c05099

[25] Feng J, Xiu Q, Huang Y, Troyer Z, Li B, Zheng L. Plant-Derived Vesicle-Like Nanoparticles as Promising Biotherapeutic Tools: Present and Future. Adv Mater (Deerfield Beach Fla). 2023;35:e2207826. 10.1002/adma.202207826 36592157

[26] Suharta S, Barlian A, Hidajah AC, Notobroto HB, Ana ID, Indariani S, Wungu TDK, Wijaya CH. Plant-derived exosome-like nanoparticles: A concise review on its extraction methods, content, bioactivities, and potential as functional food ingredient. J Food Sci. 2021;86:2838–50. 34151426 10.1111/1750-3841.15787

[27] Huang Y, Wang S, Cai Q, Jin H. Effective methods for isolation and purification of extracellular vesicles from plants. J Integr Plant Biol. 2021;63:2020–30. 34668639 10.1111/jipb.13181 PMC8972076

[28] Li A, Li D, Gu Y, Liu R, Tang X, Zhao Y, Qi F, Wei J, Liu J. Plant-derived nanovesicles: Further exploration of biomedical function and application potential. Acta Pharm Sinica B. 2023;13:3300–20. 10.1016/j.apsb.2022.12.022 PMC10465964 37655320

[29] Cao M, Diao N, Cai X, Chen X, Xiao Y, Guo C, Chen D, Zhang X. Plant exosome nanovesicles (PENs): green delivery platforms. Mater Horiz. 2023;10:3879–94. 37671650 10.1039/d3mh01030a

[30] Sarasati A, Syahruddin MH, Nuryanti A, Ana ID, Barlian A, Wijaya CH, Ratnadewi D, Wungu TDK, Takemori H. Plant-Derived Exosome-like Nanoparticles for Biomedical Applications and Regenerative Therapy, Biomedicines, 11 (2023). 10.3390/biomedicines11041053 PMC10136114 37189671

[31] Bai C, Liu J, Zhang X, Li Y, Qin Q, Song H, Yuan C, Huang Z. Research status and challenges of plant-derived exosome-like nanoparticles. Volume 174. Biomedicine & pharmacotherapy = Biomedecine & pharmacotherapie; 2024. p. 116543. 10.1016/j.biopha.2024.116543 38608523

[32] Zhao Y, Tan H, Zhang J, Pan B, Wang N, Chen T, Shi Y, Wang Z. Plant-Derived Vesicles: A New Era for Anti-Cancer Drug Delivery and Cancer Treatment. Int J Nanomed. 2023;18:6847–68. 10.2147/IJN.S432279 PMC10664809 38026523

[33] Xu X, Xu LM, Wang JZ, Wen CN, Xia J, Zhang YM, Liang YJ. Bioinspired cellular membrane-derived vesicles for mRNA delivery, Theranostics, 14 (2024) 3246–3266. 10.7150/thno.93755 PMC11155408 38855184

[34] Teng F, Fussenegger M. Shedding Light on Extracellular Vesicle Biogenesis and Bioengineering, Advanced science (Weinheim, Baden-Wurttemberg, Germany), 8 (2020) 2003505. 10.1002/advs.202003505 PMC7788585 33437589

[35] Dixson AC, Dawson TR, Di Vizio D, Weaver AM. Context-specific regulation of extracellular vesicle biogenesis and cargo selection. Nat Rev Mol Cell Biol. 2023;24:454–76. 36765164 10.1038/s41580-023-00576-0 PMC10330318

[36] Zhou Q., Ma K., Hu H., Xing X., Huang X., Gao H. Extracellular vesicles: Their functions in plant-pathogen interactions. Mol Plant Pathol. 2022;23:760–71. 10.1111/mpp.13170 PMC9104264 34873812

[37] Wang J, Ding Y, Wang J, Hillmer S, Miao Y, Lo SW, Wang X, Robinson DG, Jiang L. EXPO, an exocyst-positive organelle distinct from multivesicular endosomes and autophagosomes, mediates cytosol to cell wall exocytosis in Arabidopsis and tobacco cells. Plant Cell. 2010;22:4009–30. 21193573 10.1105/tpc.110.080697 PMC3027174

[38] Wu P, Wu W, Zhang S, Han J, Liu C, Yu H, Chen X, Chen X. Therapeutic potential and pharmacological significance of extracellular vesicles derived from traditional medicinal plants. Front Pharmacol. 2023;14:1272241. 38108066 10.3389/fphar.2023.1272241 PMC10725203

[39] Dad HA, Gu TW, Zhu AQ, Huang LQ, Peng LH. Plant Exosome-like Nanovesicles: Emerging Therapeutics and Drug Delivery Nanoplatforms. Mol therapy: J Am Soc Gene Therapy. 2021;29:13–31. 10.1016/j.ymthe.2020.11.030 PMC7791080 33278566

[40] Cui Y, Gao J, He Y, Jiang L. Plant extracellular vesicles. Protoplasma. 2020;257:3–12. 31468195 10.1007/s00709-019-01435-6

[41] Robinson DG, Ding Y, Jiang L. Unconventional protein secretion in plants: a critical assessment. Protoplasma. 2016;253:31–43. 26410830 10.1007/s00709-015-0887-1

[42] Ly NP, Han HS, Kim M, Park JH, Choi KY. Plant-derived nanovesicles: Current understanding and applications for cancer therapy. Bioactive Mater. 2023;22:365–83. 10.1016/j.bioactmat.2022.10.005 PMC9588993 36311046

[43] Wang Y, Wu Y, Shen S, Liu Y, Xia Y, Xia H, Xie Z, Xu Y. Engineered plant extracellular vesicles for natural delivery across physiological barriers. Food Funct. 2024;15:1737–57. 38284549 10.1039/d3fo03503d

[44] Wang X, Chung KP, Lin W, Jiang L. Protein secretion in plants: conventional and unconventional pathways and new techniques. J Exp Bot. 2017;69:21–37. 28992209 10.1093/jxb/erx262

[45] Hatsugai N, Iwasaki S, Tamura K, Kondo M, Fuji K, Ogasawara K, Nishimura M, Hara-Nishimura I. A novel membrane fusion-mediated plant immunity against bacterial pathogens. Genes Dev. 2009;23:2496–506. 19833761 10.1101/gad.1825209 PMC2779742

[46] Cui Y, Cao W, He Y, Zhao Q, Wakazaki M, Zhuang X, Gao J, Zeng Y, Gao C, Ding Y, Wong HY, Wong WS, Lam HK, Wang P, Ueda T, Rojas-Pierce M, Toyooka K, Kang BH, Jiang L. A whole-cell electron tomography model of vacuole biogenesis in Arabidopsis root cells. Nat plants. 2019;5:95–105. 30559414 10.1038/s41477-018-0328-1

[47] Movahed N, Cabanillas DG, Wan J, Vali H, Laliberté JF, Zheng H. Turnip Mosaic Virus Components Are Released into the Extracellular Space by Vesicles in Infected Leaves. Plant Physiol. 2019;180:1375–88. 31019004 10.1104/pp.19.00381 PMC6752911

[48] Regente M, Pinedo M, San Clemente H, Balliau T, Jamet E, de la Canal L. Plant extracellular vesicles are incorporated by a fungal pathogen and inhibit its growth. J Exp Bot. 2017;68:5485–95. 29145622 10.1093/jxb/erx355

[49] Meyer D, Pajonk S, Micali C, O’Connell R, Schulze-Lefert P. Extracellular transport and integration of plant secretory proteins into pathogen-induced cell wall compartments. Plant journal: cell Mol biology. 2009;57:986–99. 10.1111/j.1365-313X.2008.03743.x 19000165

[50] Manjithaya R, Anjard C, Loomis WF, Subramani S. Unconventional secretion of Pichia pastoris Acb1 is dependent on GRASP protein, peroxisomal functions, and autophagosome formation. J Cell Biol. 2010;188:537–46. 20156962 10.1083/jcb.200911149 PMC2828923

[51] Ambrosone A, Barbulova A, Cappetta E, Cillo F, De Palma M, Ruocco M, Pocsfalvi G. Plant Extracellular Vesicles: Current Landscape and Future Directions, Plants (Basel, Switzerland), 12 (2023). 10.3390/plants12244141 PMC10747359 38140468

[52] C. Théry, K.W. Witwer, E. Aikawa, M.J. Alcaraz, J.D. Anderson, R. Andriantsitohaina,A. Antoniou, T. Arab, F. Archer, G.K. Atkin-Smith, D.C. Ayre, J.M. Bach, D. Bachurski,H. Baharvand, L. Balaj, S. Baldacchino, N.N. Bauer, A.A. Baxter, M. Bebawy, C. Beckham,A. Bedina Zavec, A. Benmoussa, A.C. Berardi, P. Bergese, E. Bielska, C. Blenkiron,S. Bobis-Wozowicz, E. Boilard, W. Boireau, A. Bongiovanni, F.E. Borràs, S. Bosch,C.M. Boulanger, X. Breakefield, A.M. Breglio, M. Brennan, D.R. Brigstock, A. Brisson,M.L. Broekman, J.F. Bromberg, P. Bryl-Górecka, S. Buch, A.H. Buck, D. Burger, S. Busatto,D. Buschmann, B. Bussolati, E.I. Buzás, J.B. Byrd, G. Camussi, D.R. Carter, S. Caruso,L.W. Chamley, Y.T. Chang, C. Chen, S. Chen, L. Cheng, A.R. Chin, A. Clayton, S.P.Clerici, A. Cocks, E. Cocucci, R.J. Coffey, A. Cordeiro-da-Silva, Y. Couch, F.A. Coumans,B. Coyle, R. Crescitelli, M.F. Criado, C. D’Souza-Schorey, S. Das, A. Datta Chaudhuri,P. de Candia, E.F. De Santana, O. De Wever, H.A. Del Portillo, T. Demaret, S. Deville,A. Devitt, B. Dhondt, D. Di Vizio, L.C. Dieterich, V. Dolo, A.P. Dominguez Rubio,M. Dominici, M.R. Dourado, T.A. Driedonks, F.V. Duarte, H.M. Duncan, R.M. Eichenberger,K. Ekström, S. El Andaloussi, C. Elie-Caille, U. Erdbrügger, J.M. Falcón-Pérez, F.Fatima, J.E. Fish, M. Flores-Bellver, A. Försönits, A. Frelet-Barrand, F. Fricke,G. Fuhrmann, S. Gabrielsson, A. Gámez-Valero, C. Gardiner, K. Gärtner, R. Gaudin,Y.S. Gho, B. Giebel, C. Gilbert, M. Gimona, I. Giusti, D.C. Goberdhan, A. Görgens,S.M. Gorski, D.W. Greening, J.C. Gross, A. Gualerzi, G.N. Gupta, D. Gustafson, A.Handberg, R.A. Haraszti, P. Harrison, H. Hegyesi, A. Hendrix, A.F. Hill, F.H. Hochberg,K.F. Hoffmann, B. Holder, H. Holthofer, B. Hosseinkhani, G. Hu, Y. Huang, V. Huber,S. Hunt, A.G. Ibrahim, T. Ikezu, J.M. Inal, M. Isin, A. Ivanova, H.K. Jackson, S.Jacobsen, S.M. Jay, M. Jayachandran, G. Jenster, L. Jiang, S.M. Johnson, J.C. Jones,A. Jong, T. Jovanovic-Talisman, S. Jung, R. Kalluri, S.I. Kano, S. Kaur, Y. Kawamura,E.T. Keller, D. Khamari, E. Khomyakova, A. Khvorova, P. Kierulf, K.P. Kim, T. Kislinger,M. Klingeborn, D.J. Klinke, 2nd, M. Kornek, M.M. Kosanović, F. Kovács Á, E.M. Krämer-Albers, S. Krasemann, M. Krause, I.V. Kurochkin, G.D. Kusuma, S. Kuypers, S. Laitinen,S.M. Langevin, L.R. Languino, J. Lannigan, C. Lässer, L.C. Laurent, G. Lavieu, E. Lázaro-Ibáñez, S. Le Lay, M.S. Lee, Y.X.F. Lee, D.S. Lemos, M. Lenassi, A. Leszczynska, I.T.Li, K. Liao, S.F. Libregts, E. Ligeti, R. Lim, S.K. Lim, A. Linē, K. Linnemannstöns, A. Llorente, C.A. Lombard, M.J. Lorenowicz, M. Lörincz Á, J.Lötvall, J. Lovett, M.C. Lowry, X. Loyer, Q. Lu, B. Lukomska, T.R. Lunavat, S.L. Maas,H. Malhi, A. Marcilla, J. Mariani, J. Mariscal, E.S. Martens-Uzunova, L. Martin-Jaular,M.C. Martinez, V.R. Martins, M. Mathieu, S. Mathivanan, M. Maugeri, L.K. McGinnis,M.J. McVey, D.G. Meckes, Jr., K.L. Meehan, I. Mertens, V.R. Minciacchi, A. Möller,M. Møller Jørgensen, A. Morales-Kastresana, J. Morhayim, F. Mullier, M. Muraca, L.Musante, V. Mussack, D.C. Muth, K.H. Myburgh, T. Najrana, M. Nawaz, I. Nazarenko,P. Nejsum, C. Neri, T. Neri, R. Nieuwland, L. Nimrichter, J.P. Nolan, E.N. Nolte-‘t Hoen, N. Noren Hooten, L. O’Driscoll, T. O’Grady, A. O’Loghlen, T. Ochiya, M. Olivier,A. Ortiz, L.A. Ortiz, X. Osteikoetxea, O. Østergaard, M. Ostrowski, J. Park, D.M.Pegtel, H. Peinado, F. Perut, M.W. Pfaffl, D.G. Phinney, B.C. Pieters, R.C. Pink,D.S. Pisetsky, E. Pogge von Strandmann, I. Polakovicova, I.K. Poon, B.H. Powell, I.Prada, L. Pulliam, P. Quesenberry, A. Radeghieri, R.L. Raffai, S. Raimondo, J. Rak,M.I. Ramirez, G. Raposo, M.S. Rayyan, N. Regev-Rudzki, F.L. Ricklefs, P.D. Robbins,D.D. Roberts, S.C. Rodrigues, E. Rohde, S. Rome, K.M. Rouschop, A. Rughetti, A.E.Russell, P. Saá, S. Sahoo, E. Salas-Huenuleo, C. Sánchez, J.A. Saugstad, M.J. Saul,R.M. Schiffelers, R. Schneider, T.H. Schøyen, A. Scott, E. Shahaj, S. Sharma, O. Shatnyeva,F. Shekari, G.V. Shelke, A.K. Shetty, K. Shiba, P.R. Siljander, A.M. Silva, A. Skowronek,O.L. Snyder, 2nd, R.P. Soares, B.W. Sódar, C. Soekmadji, J. Sotillo, P.D. Stahl, W.Stoorvogel, S.L. Stott, E.F. Strasser, S. Swift, H. Tahara, M. Tewari, K. Timms, S.Tiwari, R. Tixeira, M. Tkach, W.S. Toh, R. Tomasini, A.C. Torrecilhas, J.P. Tosar,V. Toxavidis, L. Urbanelli, P. Vader, B.W. van Balkom, S.G. van der Grein, J. Van Deun, M.J. van Herwijnen, K. Van Keuren-Jensen, G. van Niel, M.E. van Royen, A.J.van Wijnen, M.H. Vasconcelos, I.J. Vechetti, Jr., T.D. Veit, L.J. Vella, É. Velot,F.J. Verweij, B. Vestad, J.L. Viñas, T. Visnovitz, K.V. Vukman, J. Wahlgren, D.C.Watson, M.H. Wauben, A. Weaver, J.P. Webber, V. Weber, A.M. Wehman, D.J. Weiss, J.A.Welsh, S. Wendt, A.M. Wheelock, Z. Wiener, L. Witte, J. Wolfram, A. Xagorari, P. Xander,J. Xu, X. Yan, M. Yáñez-Mó, H. Yin, Y. Yuana, V. Zappulli, J. Zarubova, V. Žėkas,J.Y. Zhang, Z. Zhao, L. Zheng, A.R. Zheutlin, A.M. Zickler, P. Zimmermann, A.M. Zivkovic,D. Zocco, E.K. Zuba-Surma, Minimal information for studies of extracellular vesicles 2018 (MISEV2018): a position statement of the International Society for Extracellular Vesicles and update of the MISEV2014 guidelines, Journal of extracellular vesicles,7 (2018) 1535750. 10.1080/20013078.2018.1535750 PMC6322352 30637094

[53] Rahmati S, Karimi H, Alizadeh M, Khazaei AH, Paiva-Santos AC, Rezakhani L, Sharifi E. Prospects of plant-derived exosome-like nanocarriers in oncology and tissue engineering. Hum Cell. 2024;37:121–38. 37878214 10.1007/s13577-023-00994-4

[54] Yıldırım M, Ünsal N, Kabataş B, Eren O, Şahin F. Effect of Solanum lycopersicum and Citrus limon-Derived Exosome-Like Vesicles on Chondrogenic Differentiation of Adipose-Derived Stem Cells. Appl Biochem Biotechnol. 2024;196:203–19. 37103740 10.1007/s12010-023-04491-0

[55] Savcı Y, Kırbaş OK, Bozkurt BT, Abdik EA, Taşlı PN, Şahin F, Abdik H. Grapefruit-derived extracellular vesicles as a promising cell-free therapeutic tool for wound healing. Food Funct. 2021;12:5144–56. 33977960 10.1039/d0fo02953j

[56] Xiao J, Feng S, Wang X, Long K, Luo Y, Wang Y, Ma J, Tang Q, Jin L, Li X, Li M. Identification of exosome-like nanoparticle-derived microRNAs from 11 edible fruits and vegetables. Volume 6. PeerJ; 2018. p. e5186. 10.7717/peerj.5186 PMC6074755 30083436

[57] Seo K, Yoo JH, Kim J, Min SJ, Heo DN, Kwon IK, Moon HJ. Ginseng-derived exosome-like nanovesicles extracted by sucrose gradient ultracentrifugation to inhibit osteoclast differentiation. Nanoscale. 2023;15:5798–808. 36857681 10.1039/d2nr07018a

[58] Man F, Meng C, Liu Y, Wang Y, Zhou Y, Ma J, Lu R. The Study of Ginger-Derived Extracellular Vesicles as a Natural Nanoscale Drug Carrier and Their Intestinal Absorption in Rats. AAPS PharmSciTech. 2021;22:206. 34297224 10.1208/s12249-021-02087-7

[59] Ramírez-Hernández AA, Reyes-Jiménez E, Velázquez-Enríquez JM, Santos-Álvarez JC, Soto-Guzmán A, Castro-Sánchez L, Tapia-Pastrana G, Torres-Aguilar H, Vásquez-Garzón VR. R. Baltiérrez-Hoyos, Zingiber officinale-Derived Extracellular Vesicles Attenuate Bleomycin-Induced Pulmonary Fibrosis Trough Antioxidant, Anti-Inflammatory and Protease Activity in a Mouse Model. Cells; 2023. p. 12. 10.3390/cells12141852 PMC10378408 37508515

[60] O’Leary BM, Rico A, McCraw S, Fones HN, Preston GM. The infiltration-centrifugation technique for extraction of apoplastic fluid from plant leaves using Phaseolus vulgaris as an example. J visualized experiments JoVE. 2014;19:52113. 10.3791/52113 PMC4396939 25549068

[61] Lohaus G, Pennewiss K, Sattelmacher B, Hussmann M, Hermann K, Muehling. Is the infiltration-centrifugation technique appropriate for the isolation of apoplastic fluid? A critical evaluation with different plant species. Physiol Plant. 2001;111:457–65. 11299010 10.1034/j.1399-3054.2001.1110405.x

[62] Prado N, Alché D, Jde J, Casado-Vela S, Mas M, Villalba R, Rodríguez E, Batanero. Nanovesicles are secreted during pollen germination and pollen tube growth: a possible role in fertilization. Mol Plant. 2014;7:573–7. 24177685 10.1093/mp/sst153

[63] Rutter BD, Innes RW. Extracellular Vesicles Isolated from the Leaf Apoplast Carry Stress-Response Proteins. Plant Physiol. 2017;173:728–41. 27837092 10.1104/pp.16.01253 PMC5210723

[64] Chen A, He B, Jin H. Isolation of Extracellular Vesicles from Arabidopsis. Curr protocols. 2022;2:e352. 10.1002/cpz1.352 PMC8931852 35030291

[65] Fujita D, Arai T, Komori H, Shirasaki Y, Wakayama T, Nakanishi T, Tamai I. Apple-Derived Nanoparticles Modulate Expression of Organic-Anion-Transporting Polypeptide (OATP) 2B1 in Caco-2 Cells. Mol Pharm. 2018;15:5772–80. 30359033 10.1021/acs.molpharmaceut.8b00921

[66] Arai M, Komori H, Fujita D, Tamai I. Uptake Pathway of Apple-derived Nanoparticle by Intestinal Cells to Deliver its Cargo. Pharm Res. 2021;38:523–30. 33723795 10.1007/s11095-021-03018-8

[67] Perut F, Roncuzzi L, Avnet S, Massa A, Zini N, Sabbadini S, Giampieri F, Mezzetti B, Baldini N. Strawberry-Derived Exosome-Like Nanoparticles Prevent Oxidative Stress in Human Mesenchymal Stromal Cells. Volume 11. Biomolecules; 2021. 10.3390/biom11010087 PMC7828105 33445656

[68] Castelli G, Logozzi M, Mizzoni D, Di Raimo R, Cerio A, Dolo V, Pasquini L, Screnci M, Ottone T, Testa U, Fais S, Pelosi E. Ex vivo anti-leukemic effect of exosome-like grapefruit-derived nanovesicles from organic farming-the potential role of ascorbic acid. Int J Mol Sci. 2023;24:15663. 37958646 10.3390/ijms242115663 PMC10648274

[69] Stanly C, Alfieri M, Ambrosone A, Leone A, Fiume I, Pocsfalvi G. Grapefruit-Derived Micro and Nanovesicles Show Distinct Metabolome Profiles and Anticancer Activities in the A375 Human Melanoma Cell Line. Cells; 2020. p. 9. 10.3390/cells9122722 PMC7766354 33371199

[70] Moon K, Hur J, Kim KP, Lee K, Kang JY. Surface-functionalizable plant‐derived extracellular vesicles for targeted drug delivery carrier using grapefruit. Adv Mater Interfaces. 2023;10:2300220.

[71] Garaeva L, Kamyshinsky R, Kil Y, Varfolomeeva E, Verlov N, Komarova E, Garmay Y, Landa S, Burdakov V, Myasnikov A, Vinnikov IA, Margulis B, Guzhova I, Kagansky A, Konevega AL, Shtam T. Delivery of functional exogenous proteins by plant-derived vesicles to human cells in vitro. Sci Rep. 2021;11:6489. 33753795 10.1038/s41598-021-85833-y PMC7985202

[72] Zhang M, Viennois E, Prasad M, Zhang Y, Wang L, Zhang Z, Han MK, Xiao B, Xu C, Srinivasan S, Merlin D. Edible ginger-derived nanoparticles: A novel therapeutic approach for the prevention and treatment of inflammatory bowel disease and colitis-associated cancer. Biomaterials. 2016;101:321–40. 27318094 10.1016/j.biomaterials.2016.06.018 PMC4921206

[73] Zhang M, Yang C, Yan X, Sung J, Garg P, Merlin D. Highly Biocompatible Functionalized Layer-by-Layer Ginger Lipid Nano Vectors Targeting P-selectin for Delivery of Doxorubicin to Treat Colon Cancer. Adv Ther (Weinh), 2019; 2: 1900129. 10.1002/adtp.201900129 PMC7546358 33043129

[74] Li Z, Wang H, Yin H, Bennett C, Zhang HG, Guo P. Arrowtail RNA for Ligand Display on Ginger Exosome-like Nanovesicles to Systemic Deliver siRNA for Cancer Suppression. Sci Rep. 2018;8:14644. 30279553 10.1038/s41598-018-32953-7 PMC6168523

[75] Zhuang X, Deng ZB, Mu J, Zhang L, Yan J, Miller D, Feng W, McClain CJ, Zhang HG. Ginger-derived nanoparticles protect against alcohol-induced liver damage. J Extracell vesicles. 2015;4:28713. 26610593 10.3402/jev.v4.28713 PMC4662062

[76] Cao M, Yan H, Han X, Weng L, Wei Q, Sun X, Lu W, Wei Q, Ye J, Cai X, Hu C, Yin X, Cao P. Ginseng-derived nanoparticles alter macrophage polarization to inhibit melanoma growth. J Immunother Cancer. 2019;7:326. 31775862 10.1186/s40425-019-0817-4 PMC6882204

[77] Kim J, Lee Y-H, Wang J, Kim YK, Kwon IK. Isolation and characterization of ginseng-derived exosome-like nanoparticles with sucrose cushioning followed by ultracentrifugation. SN Appl Sci. 2022;4:63.

[78] Xu XH, Yuan TJ, Dad HA, Shi MY, Huang YY, Jiang ZH, Peng LH. Plant Exosomes As Novel Nanoplatforms for MicroRNA Transfer Stimulate Neural Differentiation of Stem Cells In Vitro and In Vivo. Nano Lett. 2021;21:8151–9. 34586821 10.1021/acs.nanolett.1c02530

[79] Feng W, Teng Y, Zhong Q, Zhang Y, Zhang J, Zhao P, Chen G, Wang C, Liang XJ, Ou C. Biomimetic Grapefruit-Derived Extracellular Vesicles for Safe and Targeted Delivery of Sodium Thiosulfate against Vascular Calcification. ACS Nano. 2023;17:24773–89. 38055864 10.1021/acsnano.3c05261 PMC10753875

[80] Wang Q, Zhuang X, Mu J, Deng ZB, Jiang H, Zhang L, Xiang X, Wang B, Yan J, Miller D, Zhang HG. Corrigendum: Delivery of therapeutic agents by nanoparticles made of grapefruit-derived lipids, Nat Commun, 7 (2016) 11347. 10.1038/ncomms11347 PMC4842968 27094443

[81] Zhao X, Yin F, Fu L, Ma Y, Ye L, Huang Y, Fan W, Gao W, Cai Y, Mou X. Garlic-derived exosome-like nanovesicles as a hepatoprotective agent alleviating acute liver failure by inhibiting CCR2/CCR5 signaling and inflammation. Biomaterials Adv. 2023;154:213592. 10.1016/j.bioadv.2023.213592 37717364

[82] Zhou S, Huang P, Cao Y, Hua X, Yang Y, Liu S. Garlic-Derived Exosome-like Nanovesicles-Based Wound Dressing for Staphylococcus aureus Infection Visualization and Treatment. ACS Appl bio Mater. 2024;7:1888–98. 38349328 10.1021/acsabm.3c01256

[83] De Robertis M, Sarra A, D’Oria V, Mura F, Bordi F, Postorino P, Fratantonio D. Blueberry-Derived Exosome-Like Nanoparticles Counter the Response to TNF-α-Induced Change on Gene Expression in EA.hy926 Cells, Biomolecules, 10 (2020). 10.3390/biom10050742 PMC7277966 32397678

[84] Sánchez-López CM, Manzaneque-López MC, Pérez-Bermúdez P, Soler C, Marcilla A. Characterization and bioactivity of extracellular vesicles isolated from pomegranate. Food Funct. 2022;13:12870–82. 36441623 10.1039/d2fo01806c

[85] Kalarikkal SP, Prasad D, Kasiappan R, Chaudhari SR, Sundaram GM. A cost-effective polyethylene glycol-based method for the isolation of functional edible nanoparticles from ginger rhizomes. Sci Rep. 2020;10:4456. 32157137 10.1038/s41598-020-61358-8 PMC7064537

[86] Suresh AP, Kalarikkal SP, Pullareddy B, Sundaram GM. Low pH-Based Method to Increase the Yield of Plant-Derived Nanoparticles from Fresh Ginger Rhizomes. ACS omega. 2021;6:17635–41. 34278148 10.1021/acsomega.1c02162 PMC8280662

[87] Yang M, Liu X, Luo Q, Xu L, Chen F. An efficient method to isolate lemon derived extracellular vesicles for gastric cancer therapy. J Nanobiotechnol. 2020;18:100. 10.1186/s12951-020-00656-9 PMC7370524 32690102

[88] Yang M, Luo Q, Chen X, Chen F. Bitter melon derived extracellular vesicles enhance the therapeutic effects and reduce the drug resistance of 5-fluorouracil on oral squamous cell carcinoma. J Nanobiotechnol. 2021;19:259. 10.1186/s12951-021-00995-1 PMC8400897 34454534

[89] Jackson KK, Mata C, Marcus RK. A rapid capillary-channeled polymer (C-CP) fiber spin-down tip approach for the isolation of plant-derived extracellular vesicles (PDEVs) from 20 common fruit and vegetable sources. Talanta. 2023;252:123779. 35994804 10.1016/j.talanta.2022.123779

[90] Kang SJ, Kim SE, Seo M-J, Kim E, Rhee WJ. Suppression of inflammatory responses in macrophages by onion-derived extracellular vesicles. J Ind Eng Chem. 2022;115:287–97.

[91] Şahin F, Koçak P, Güneş MY, Özkan İ, Yıldırım E, Kala EY. In Vitro Wound Healing Activity of Wheat-Derived Nanovesicles. Appl Biochem Biotechnol. 2019;188:381–94. 30474796 10.1007/s12010-018-2913-1

[92] Nguyen TN, Pham CV, Chowdhury R, Patel S, Jaysawal SK, Hou Y, Xu H, Jia L, Duan A, Tran PH, Duan W. Development of Blueberry-Derived Extracellular Nanovesicles for Immunomodulatory Therapy, Pharmaceutics, 15 (2023). 10.3390/pharmaceutics15082115 PMC10458573 37631329

[93] Zhang J, Qiu Y, Xu K. Characterization of GFP-AtPEN1 as a marker protein for extracellular vesicles isolated from Nicotiana benthamiana leaves. Volume 15. Plant signaling & behavior; 2020. p. 1791519. 10.1080/15592324.2020.1791519 PMC8550176 32657215

[94] Liu Y, Wu S, Koo Y, Yang A, Dai Y, Khant H, Osman SR, Chowdhury M, Wei H, Li Y, Court K, Hwang E, Wen Y, Dasari SK, Nguyen M, Tang EC, Chehab EW, de Val N, Braam J, Sood AK. Characterization of and isolation methods for plant leaf nanovesicles and small extracellular vesicles. Nanomedicine. 2020;29:102271. 32702466 10.1016/j.nano.2020.102271

[95] Woith E, Melzig MF. Extracellular vesicles from fresh and dried plants-simultaneous purification and visualization using gel electrophoresis. Int J Mol Sci. 2019;20:357. 30654488 10.3390/ijms20020357 PMC6359398

[96] Vader P, Mol EA, Pasterkamp G, Schiffelers RM. Extracellular vesicles for drug delivery. Adv Drug Deliv Rev. 2016;106:148–56. 26928656 10.1016/j.addr.2016.02.006

[97] Gardiner C, Di Vizio D, Sahoo S, Théry C, Witwer KW, Wauben M, Hill AF. Techniques used for the isolation and characterization of extracellular vesicles: results of a worldwide survey. J Extracell vesicles. 2016;5:32945. 27802845 10.3402/jev.v5.32945 PMC5090131

[98] Shao H, Im H, Castro CM, Breakefield X, Weissleder R, Lee H. New Technologies for Analysis of Extracellular Vesicles. Chem Rev. 2018;118:1917–50. 29384376 10.1021/acs.chemrev.7b00534 PMC6029891

[99] Cong M, Tan S, Li S, Gao L, Huang L, Zhang HG, Qiao H. Technology insight: Plant-derived vesicles-How far from the clinical biotherapeutics and therapeutic drug carriers? Adv Drug Deliv Rev. 2022;182:114108. 34990792 10.1016/j.addr.2021.114108

[100] Kilasoniya A, Garaeva L, Shtam T, Spitsyna A, Putevich E, Moreno-Chamba B, Salazar-Bermeo J, Komarova E, Malek A, Valero M, Saura D. Potential of Plant Exosome Vesicles from Grapefruit (Citrus × paradisi) and Tomato (Solanum lycopersicum) Juices as Functional Ingredients and Targeted Drug Delivery Vehicles, Antioxidants (Basel, Switzerland), 12 (2023). 10.3390/antiox12040943 PMC10135875 37107317

[101] Crescitelli R, Lässer C, Lötvall J. Isolation and characterization of extracellular vesicle subpopulations from tissues. Nat Protoc. 2021;16:1548–80. 33495626 10.1038/s41596-020-00466-1

[102] Min L, Wang B, Bao H, Li X, Zhao L, Meng J, Wang S. Advanced Nanotechnologies for Extracellular Vesicle-Based Liquid Biopsy. Adv Sci (Weinheim Baden-Wurttemberg Germany). 2021;8:e2102789. 10.1002/advs.202102789 PMC8529441 34463056

[103] Chen J, Li P, Zhang T, Xu Z, Huang X, Wang R, Du L. Review on Strategies and Technologies for Exosome Isolation and Purification. Front Bioeng Biotechnol. 2021;9:811971. 35071216 10.3389/fbioe.2021.811971 PMC8766409

[104] Iwai K, Minamisawa T, Suga K, Yajima Y, Shiba K. Isolation of human salivary extracellular vesicles by iodixanol density gradient ultracentrifugation and their characterizations. J Extracell vesicles. 2016;5:30829. 27193612 10.3402/jev.v5.30829 PMC4871899

[105] Yang D, Zhang W, Zhang H, Zhang F, Chen L, Ma L, Larcher LM, Chen S, Liu N, Zhao Q, Tran PHL, Chen C, Veedu RN, Wang T. Progress, opportunity, and perspective on exosome isolation - efforts for efficient exosome-based theranostics. Theranostics. 2020;10:3684–707. 32206116 10.7150/thno.41580 PMC7069071

[106] Lee R, Ko HJ, Kim K, Sohn Y, Min SY, Kim JA, Na D, Yeon JH. Anti-melanogenic effects of extracellular vesicles derived from plant leaves and stems in mouse melanoma cells and human healthy skin. J Extracell vesicles. 2020;9:1703480. 32002169 10.1080/20013078.2019.1703480 PMC6968621

[107] Monguió-Tortajada M, Gálvez-Montón C, Bayes-Genis A, Roura S, Borràs FE. Extracellular vesicle isolation methods: rising impact of size-exclusion chromatography, Cellular and molecular life sciences. Volume 76. CMLS; 2019. pp. 2369–82. 10.1007/s00018-019-03071-y PMC11105396 30891621

[108] Mol EA, Goumans MJ, Doevendans PA, Sluijter JPG, Vader P. Higher functionality of extracellular vesicles isolated using size-exclusion chromatography compared to ultracentrifugation. Nanomedicine. 2017;13:2061–5. 28365418 10.1016/j.nano.2017.03.011

[109] You JY, Kang SJ, Rhee WJ. Isolation of cabbage exosome-like nanovesicles and investigation of their biological activities in human cells. Bioactive Mater. 2021;6:4321–32. 10.1016/j.bioactmat.2021.04.023 PMC8105599 33997509

[110] Li P, Kaslan M, Lee SH, Yao J, Gao Z. Progress Exosome Isolation Techniques Theranostics. 2017;7:789–804. 28255367 10.7150/thno.18133 PMC5327650

[111] Wang Z, Zhou X, Kong Q, He H, Sun J, Qiu W, Zhang L, Yang M. Extracellular Vesicle Preparation and Analysis: A State-of-the-Art Review. Adv Sci (Weinheim Baden-Wurttemberg Germany). 2024;11:e2401069. 10.1002/advs.202401069 PMC11321646 38874129

[112] Cai Q, Qiao L, Wang M, He B, Lin F.M., Palmquist J, Huang S.D., Jin H. Plants send small RNAs in extracellular vesicles to fungal pathogen to silence virulence genes. Sci (New York N Y). 2018;360:1126–9. 10.1126/science.aar4142 PMC6442475 29773668

[113] He B, Cai Q, Qiao L, Huang CY, Wang S, Miao W, Ha T, Wang Y, Jin H. RNA-binding proteins contribute to small RNA loading in plant extracellular vesicles. Nat plants. 2021;7:342–52. 33633358 10.1038/s41477-021-00863-8 PMC7979528

[114] Wen Z, Yu J, Jeong H, Kim DU, Yang JY, Hyun KA, Choi S, Park S, Jung HI. An all-in-one platform to deplete pathogenic bacteria for rapid and safe enrichment of plant-derived extracellular vesicles. Lab Chip. 2023;23:4483–92. 37750717 10.1039/d3lc00585b

[115] Tiwari S, Kumar V, Randhawa S, Verma SK. Preparation and characterization of extracellular vesicles, American journal of reproductive immunology (New York, N.Y.: 1989), 85 (2021) e13367. 10.1111/aji.13367 33118232

[116] Rider MA, Hurwitz SN, Meckes DG Jr. ExtraPEG: A Polyethylene Glycol-Based Method for Enrichment of Extracellular Vesicles. Sci Rep. 2016;6:23978. 27068479 10.1038/srep23978 PMC4828635

[117] Zhao Q, Liu G, Liu F, Xie M, Zou Y, Wang S, Guo Z, Dong J, Ye J, Cao Y, Zheng L, Zhao K. An enzyme-based system for extraction of small extracellular vesicles from plants. Sci Rep. 2023;13:13931. 37626167 10.1038/s41598-023-41224-z PMC10457285

[118] Tauro BJ, Greening DW, Mathias RA, Ji H, Mathivanan S, Scott AM, Simpson RJ. Comparison of ultracentrifugation, density gradient separation, and immunoaffinity capture methods for isolating human colon cancer cell line LIM1863-derived exosomes. Methods (San Diego Calif). 2012;56:293–304. 22285593 10.1016/j.ymeth.2012.01.002

[119] Zhang J, Nguyen LTH, Hickey R, Walters N, Wang X, Kwak KJ, Lee LJ, Palmer AF, Reátegui E. Immunomagnetic sequential ultrafiltration (iSUF) platform for enrichment and purification of extracellular vesicles from biofluids. Sci Rep. 2021;11:8034. 33850163 10.1038/s41598-021-86910-y PMC8044115

[120] Sidhom K, Obi PO, Saleem A. A review of exosomal isolation methods: is size exclusion chromatography the best option? Int J Mol Sci. 2020;21:6466. 32899828 10.3390/ijms21186466 PMC7556044

[121] Fang X, Chen C, Liu B, Ma Z, Hu F, Li H, Gu H, Xu H. A magnetic bead-mediated selective adsorption strategy for extracellular vesicle separation and purification. Acta Biomater. 2021;124:336–47. 33578055 10.1016/j.actbio.2021.02.004

[122] Sundaram PM, Casadei L, Lopez G, Braggio D, Balakirsky G, Pollock R, Prakash S. Multi-Layer Micro-Nanofluidic Device for Isolation and Capture of Extracellular Vesicles Derived from Liposarcoma Cell Conditioned Media. J microelectromechanical systems: joint IEEE ASME publication microstructures microactuators microsensors microsystems. 2020;29:776–82. 10.1109/jmems.2020.3006786 PMC7839931 33519169

[123] Alzahrani FA, Khan MI, Kameli N, Alsahafi E, Riza YM. Plant-Derived Extracellular Vesicles and Their Exciting Potential as the Future of Next-Generation Drug Delivery, Biomolecules, 13 (2023). 10.3390/biom13050839 PMC10216196 37238708

[124] Kocholata M, Prusova M, Auer Malinska H, Maly J, Janouskova O. Comparison of two isolation methods of tobacco-derived extracellular vesicles, their characterization and uptake by plant and rat cells. Sci Rep. 2022;12:19896. 36400817 10.1038/s41598-022-23961-9 PMC9674704

[125] Feng T, Wan Y, Dai B, Liu Y. Anticancer Activity of Bitter Melon-Derived Vesicles Extract against Breast Cancer. Cells; 2023. p. 12. 10.3390/cells12060824 PMC10047160 36980165

[126] Bhattacharjee S. DLS and zeta potential - What they are and what they are not? J Control Release. 2016;235:337–51. 27297779 10.1016/j.jconrel.2016.06.017

[127] Szatanek R, Baj-Krzyworzeka M, Zimoch J, Lekka M, Siedlar M, Baran J. The methods of choice for extracellular vesicles (EVs) characterization. Int J Mol Sci. 2017;18:1153. 28555055 10.3390/ijms18061153 PMC5485977

[128] Chan MY, Dowling QM, Sivananthan SJ, Kramer RM. Particle Sizing of Nanoparticle Adjuvant Formulations by Dynamic Light Scattering (DLS) and Nanoparticle Tracking Analysis (NTA), Methods in molecular biology. (Clifton N J). 2017;1494:239–52. 10.1007/978-1-4939-6445-1_17 27718198

[129] Ju S, Mu J, Dokland T, Zhuang X, Wang Q, Jiang H, Xiang X, Deng ZB, Wang B, Zhang L, Roth M, Welti R, Mobley J, Jun Y, Miller D, Zhang HG. Grape exosome-like nanoparticles induce intestinal stem cells and protect mice from DSS-induced colitis, Molecular therapy. J Am Soc Gene Therapy. 2013;21:1345–57. 10.1038/mt.2013.64 PMC3702113 23752315

[130] Wang B, Zhuang X, Deng ZB, Jiang H, Mu J, Wang Q, Xiang X, Guo H, Zhang L, Dryden G, Yan J, Miller D, Zhang HG. Targeted drug delivery to intestinal macrophages by bioactive nanovesicles released from grapefruit. Mol therapy: J Am Soc Gene Therapy. 2014;22:522–34. 10.1038/mt.2013.190 PMC3944329 23939022

[131] Li D, Cao G, Yao X, Yang Y, Yang D, Liu N, Yuan Y, Nishinari K, Yang X. Tartary buckwheat-derived exosome-like nanovesicles against starch digestion and their interaction mechanism. Food Hydrocolloids. 2023;141:108739.

[132] Pang W, Zuo Z, Sun W, Zhang Z, Wang J, Wang Y, Zhang D. Kidney bean derived exosome-like nanovesicles ameliorate high-fat diet-induced obesity via reshaping gut microbiota. J Funct Foods. 2024;113:105997.

[133] Zhao WJ, Bian YP, Wang QH, Yin F, Yin L, Zhang YL, Liu JH. Blueberry-derived exosomes-like nanoparticles ameliorate nonalcoholic fatty liver disease by attenuating mitochondrial oxidative stress. Acta Pharmacol Sin. 2022;43:645–58. 33990765 10.1038/s41401-021-00681-w PMC8888548

[134] Yuana Y, Koning RI, Kuil ME, Rensen PC, Koster AJ, Bertina RM, Osanto S. Cryo-electron microscopy of extracellular vesicles in fresh plasma. J Extracell vesicles. 2013;2:21494. 10.3402/jev.v2i0.21494 PMC3895263 24455109

[135] Arraud N, Linares R, Tan S, Gounou C, Pasquet JM, Mornet S, Brisson AR. Extracellular vesicles from blood plasma: determination of their morphology, size, phenotype and concentration. J Thromb haemostasis: JTH. 2014;12:614–27. 24618123 10.1111/jth.12554

[136] Vorselen D, van Dommelen SM, Sorkin R, Piontek MC, Schiller J, Döpp ST, Kooijmans SAA, van Oirschot BA, Versluijs BA, Bierings MB, van Wijk R, Schiffelers RM, Wuite GJL, Roos WH. The fluid membrane determines mechanics of erythrocyte extracellular vesicles and is softened in hereditary spherocytosis. Nat Commun. 2018;9:4960. 30470753 10.1038/s41467-018-07445-x PMC6251882

[137] Liu H, Luo GF, Shang Z. [Not Available] Acta Pharm Sinica B. 2024;14:133–54. 10.1016/j.apsb.2023.08.033 PMC10792991 38239235

[138] Cai Q, He B, Wang S, Fletcher S, Niu D, Mitter N, Birch PRJ, Jin H. Message in a Bubble: Shuttling Small RNAs and Proteins Between Cells and Interacting Organisms Using Extracellular Vesicles. Annu Rev Plant Biol. 2021;72:497–524. 34143650 10.1146/annurev-arplant-081720-010616 PMC8369896

[139] Stanly C, Fiume I, Capasso G, Pocsfalvi G. Isolation of Exosome-Like Vesicles from Plants by Ultracentrifugation on Sucrose/Deuterium Oxide (D2O) Density Cushions, Methods in molecular biology. (Clifton N J). 2016;1459:259–69. 10.1007/978-1-4939-3804-9_18 27665565

[140] Berger E, Colosetti P, Jalabert A, Meugnier E, Wiklander OPB, Jouhet J, Errazurig-Cerda E, Chanon S, Gupta D, Rautureau GJP, Geloen A, El-Andaloussi S, Panthu B, Rieusset J, Rome S. Use of Nanovesicles from Orange Juice to Reverse Diet-Induced Gut Modifications in Diet-Induced Obese Mice, Molecular therapy. Volume 18. Methods & clinical development; 2020. pp. 880–92. 10.1016/j.omtm.2020.08.009 PMC7481887 32953937

[141] Cao Y, Tan X, Shen J, Liu F, Xu Y, Chen Y, Zhou S, Qiu T, Li D, Zhao Q, Zhao K. Morinda Officinalis-derived extracellular vesicle-like particles: Anti-osteoporosis effect by regulating MAPK signaling pathway. Phytomedicine: Int J phytotherapy phytopharmacology. 2024;129:155628. 10.1016/j.phymed.2024.155628 38663117

[142] Ren J, He W, Zheng L, Duan H. From structures to functions: insights into exosomes as promising drug delivery vehicles. Biomater Sci. 2016;4:910–21. 26977477 10.1039/c5bm00583c

[143] Minciacchi VR, Freeman MR, Di Vizio D. Extracellular vesicles in cancer: exosomes, microvesicles and the emerging role of large oncosomes. Semin Cell Dev Biol. 2015;40:41–51. 25721812 10.1016/j.semcdb.2015.02.010 PMC4747631

[144] Karamanidou T, Tsouknidas A. Plant-derived extracellular vesicles as therapeutic nanocarriers. Int J Mol Sci. 2022;23:191. 10.3390/ijms23010191 PMC8745116 35008617

[145] Teng Y, Ren Y, Sayed M, Hu X, Lei C, Kumar A, Hutchins E, Mu J, Deng Z, Luo C, Sundaram K, Sriwastva MK, Zhang L, Hsieh M, Reiman R, Haribabu B, Yan J, Jala VR, Miller DM, Van Keuren-Jensen K, Merchant ML, McClain CJ, Park JW, Egilmez NK, Zhang HG. Plant-Derived Exosomal MicroRNAs Shape the Gut Microbiota, Cell host & microbe, 24 (2018) 637–e652638. 10.1016/j.chom.2018.10.001 PMC6746408 30449315

[146] Wang X, Devaiah SP, Zhang W, Welti R. Signaling functions of phosphatidic acid. Prog Lipid Res. 2006;45:250–78. 16574237 10.1016/j.plipres.2006.01.005

[147] Bokka R, Ramos AP, Fiume I, Manno M, Raccosta S, Turiák L, Sugár S, Adamo G, Csizmadia T, Pocsfalvi G. Biomanufacturing of Tomato-Derived Nanovesicles, Foods (Basel, Switzerland), 9 (2020). 10.3390/foods9121852 PMC7764365 33322632

[148] Lee BH, Wu SC, Chien HY, Shen TL, Hsu WH. Tomato-fruit-derived extracellular vesicles inhibit Fusobacterium nucleatum via lipid-mediated mechanism. Food Funct. 2023;14:8942–50. 37723977 10.1039/d3fo01608k

[149] Kumar A, Sundaram K, Teng Y, Mu J, Sriwastva MK, Zhang L, Hood JL, Yan J, Zhang X, Park JW, Merchant ML, Zhang HG. Ginger nanoparticles mediated induction of Foxa2 prevents high-fat diet-induced insulin resistance. Theranostics. 2022;12:1388–403. 35154496 10.7150/thno.62514 PMC8771553

[150] Zeng L, Wang H, Shi W, Chen L, Chen T, Chen G, Wang W, Lan J, Huang Z, Zhang J, Chen J. Aloe derived nanovesicle as a functional carrier for indocyanine green encapsulation and phototherapy. J Nanobiotechnol. 2021;19:439. 10.1186/s12951-021-01195-7 PMC8686546 34930289

[151] Liu NJ, Wang N, Bao JJ, Zhu HX, Wang LJ, Chen XY. Lipidomic analysis reveals the importance of GIPCs in arabidopsis leaf extracellular vesicles. Mol Plant. 2020;13:1523–32. 32717349 10.1016/j.molp.2020.07.016

[152] Wang Q, Ren Y, Mu J, Egilmez NK, Zhuang X, Deng Z, Zhang L, Yan J, Miller D, Zhang HG. Grapefruit-Derived Nanovectors Use an Activated Leukocyte Trafficking Pathway to Deliver Therapeutic Agents to Inflammatory Tumor Sites. Cancer Res. 2015;75:2520–9. 25883092 10.1158/0008-5472.CAN-14-3095 PMC4470740

[153] Zhang M, Wang X, Han MK, Collins JF, Merlin D. Oral administration of ginger-derived nanolipids loaded with siRNA as a novel approach for efficient siRNA drug delivery to treat ulcerative colitis. Nanomed (London England). 2017;12:1927–43. 10.2217/nnm-2017-0196 PMC5827822 28665164

[154] Bligh EG, Dyer WJ. A rapid method of total lipid extraction and purification. Can J Biochem Physiol. 1959;37:911–7. 13671378 10.1139/o59-099

[155] Mu N, Li J, Zeng L, You J, Li R, Qin A, Liu X, Yan F, Zhou Z. Plant-Derived Exosome-Like Nanovesicles: Current Progress and Prospects. Int J Nanomed. 2023;18:4987–5009. 10.2147/IJN.S420748 PMC10492547 37693885

[156] Stanly C, Moubarak M, Fiume I, Turiák L, Pocsfalvi G. Membrane transporters in citrus clementina fruit juice-derived nanovesicles. Int J Mol Sci. 2019;20:6205. 31835328 10.3390/ijms20246205 PMC6941005

[157] Raimondo S, Naselli F, Fontana S, Monteleone F, Lo Dico A, Saieva L, Zito G, Flugy A, Manno M, Di Bella MA, De Leo G, Alessandro R. Citrus limon-derived nanovesicles inhibit cancer cell proliferation and suppress CML xenograft growth by inducing TRAIL-mediated cell death. Oncotarget. 2015;6:19514–27. 26098775 10.18632/oncotarget.4004 PMC4637302

[158] Liu B, Li X, Yu H, Shi X, Zhou Y, Alvarez S, Naldrett MJ, Kachman SD, Ro SH, Sun X, Chung S, Jing L, Yu J. Therapeutic potential of garlic chive-derived vesicle-like nanoparticles in NLRP3 inflammasome-mediated inflammatory diseases. Theranostics. 2021;11:9311–30. 34646372 10.7150/thno.60265 PMC8490522

[159] Pocsfalvi G, Turiák L, Ambrosone A, Del Gaudio P, Puska G, Fiume I, Silvestre T, Vékey K. Protein biocargo of citrus fruit-derived vesicles reveals heterogeneous transport and extracellular vesicle populations. J Plant Physiol. 2018;229:111–21. 30056374 10.1016/j.jplph.2018.07.006

[160] Zhang H, Zhou JF, Kan Y, Shan JX, Ye WW, Dong NQ, Guo T, Xiang YH, Yang YB, Li YC, Zhao HY, Yu HX, Lu ZQ, Guo SQ, Lei JJ, Liao B, Mu XR, Cao YJ, Yu JJ, Lin Y, Lin HX. A genetic module at one locus in rice protects chloroplasts to enhance thermotolerance. Volume 376. Science; 2022. pp. 1293–300. (New York, N.Y.). 10.1126/science.abo5721 35709289

[161] Cavalieri D, Rizzetto L, Tocci N, Rivero D, Asquini E, Si-Ammour A, Bonechi E, Ballerini C, Viola R. Plant microRNAs as novel immunomodulatory agents. Sci Rep. 2016;6:25761. 27167363 10.1038/srep25761 PMC4863160

[162] Teng Y, Xu F, Zhang X, Mu J, Sayed M, Hu X, Lei C, Sriwastva M, Kumar A, Sundaram K, Zhang L, Park JW, Chen SY, Zhang S, Yan J, Merchant ML, Zhang X, McClain CJ, Wolfe JK, Adcock RS, Chung D, Palmer KE, Zhang HG. Plant-derived exosomal microRNAs inhibit lung inflammation induced by exosomes SARS-CoV-2 Nsp12. Mol Therapy J Amer Soc Gene Therapy. 2021;29:2424–40. 10.1016/j.ymthe.2021.05.005 PMC8110335 33984520

[163] Cartalas J, Coudray L, Gobert A. How RNases shape mitochondrial transcriptomes. Int J Mol Sci. 2022;23:6141. 35682820 10.3390/ijms23116141 PMC9181182

[164] Zhou Q, Li M, Wang X, Li Q, Wang T, Zhu Q, Zhou X, Wang X, Gao X, Li X. Immune-related microRNAs are abundant in breast milk exosomes. Int J Biol Sci. 2012;8:118–23. 22211110 10.7150/ijbs.8.118 PMC3248653

[165] Woith E, Guerriero G, Hausman JF, Renaut J, Leclercq CC, Weise C, Legay S, Weng A, Melzig MF. Plant Extracellular Vesicles and Nanovesicles: Focus on Secondary Metabolites, Proteins and Lipids with Perspectives on Their Potential and Sources. Int J Mol Sci. 2021;22:3719. 33918442 10.3390/ijms22073719 PMC8038311

[166] Gao C, Zhou Y, Chen Z, Li H, Xiao Y, Hao W, Zhu Y, Vong CT, Farag MA, Wang Y, Wang S. Turmeric-derived nanovesicles as novel nanobiologics for targeted therapy of ulcerative colitis. Theranostics. 2022;12:5596–614. 35910802 10.7150/thno.73650 PMC9330521

[167] Chen Q, Zu M, Gong H, Ma Y, Sun J, Ran S, Shi X, Zhang J, Xiao B. Tea leaf-derived exosome-like nanotherapeutics retard breast tumor growth by pro-apoptosis and microbiota modulation. J Nanobiotechnol. 2023;21:6. 10.1186/s12951-022-01755-5 PMC9811040 36600299

[168] Chen Q, Li Q, Liang Y, Zu M, Chen N, Canup BSB, Luo L, Wang C, Zeng L, Xiao B. Natural exosome-like nanovesicles from edible tea flowers suppress metastatic breast cancer via ROS generation and microbiota modulation. Acta Pharm Sinica B. 2022;12:907–23. 10.1016/j.apsb.2021.08.016 PMC8897038 35256954

[169] Mammadova R, Maggio S, Fiume I, Bokka R, Moubarak M, Gellén G, Schlosser G, Adamo G, Bongiovanni A, Trepiccione F, Guescini M, Pocsfalvi G. Protein Biocargo and Anti-Inflammatory Effect of Tomato Fruit-Derived Nanovesicles Separated by Density Gradient Ultracentrifugation and Loaded with Curcumin. Pharmaceutics; 2023. p. 15. 10.3390/pharmaceutics15020333 PMC9961453 36839657

[170] Li C, Song Q, Yin X, Song R, Chen G. Preparation, Characterization, and In Vitro Anticancer Activity Evaluation of Broccoli-Derived Extracellular Vesicle-Coated Astaxanthin Nanoparticles, Molecules (Basel, Switzerland), 27 (2022). 10.3390/molecules27123955 PMC9230617 35745077

[171] Chen X, Zhou Y, Yu J. Exosome-like nanoparticles from ginger rhizomes inhibited NLRP3 inflammasome activation. Mol Pharm. 2019;16:2690–9. 31038962 10.1021/acs.molpharmaceut.9b00246

[172] Wu JY, Li YJ, Hu XB, Huang S, Xiang DX. Preservation of small extracellular vesicles for functional analysis and therapeutic applications: a comparative evaluation of storage conditions. Drug Deliv. 2021;28:162–70. 33427518 10.1080/10717544.2020.1869866 PMC7808382

[173] Gelibter S, Marostica G, Mandelli A, Siciliani S, Podini P, Finardi A, Furlan R. The impact of storage on extracellular vesicles: A systematic study. J Extracell vesicles. 2022;11:e12162. 35102719 10.1002/jev2.12162 PMC8804350

[174] Nemidkanam V, Chaichanawongsaroj N. Characterizing Kaempferia parviflora extracellular vesicles, a nanomedicine candidate. PLoS ONE. 2022;17:e0262884. 35077499 10.1371/journal.pone.0262884 PMC8789119

[175] Zhang M, Xiao B, Wang H, Han MK, Zhang Z, Viennois E, Xu C, Merlin D. Edible Ginger-derived Nano-lipids Loaded with Doxorubicin as a Novel Drug-delivery Approach for Colon Cancer Therapy. Mol Therapy J Amer Soc Gene Therapy. 2016;24:1783–96. 10.1038/mt.2016.159 PMC5112046 27491931

[176] Charoenviriyakul C, Takahashi Y, Nishikawa M, Takakura Y. Preservation of exosomes at room temperature using lyophilization. Int J Pharm. 2018;553:1–7. 30316791 10.1016/j.ijpharm.2018.10.032

[177] Ruzycka-Ayoush M, Nowicka AM, Kowalczyk A, Gluchowska A, Targonska A, Mosieniak G, Sobczak K, Donten M, Grudzinski IP. Exosomes derived from lung cancer cells: Isolation, characterization, and stability studies. Eur J Pharm sciences: official J Eur Federation Pharm Sci. 2023;181:106369. 10.1016/j.ejps.2022.106369 36572357

[178] Zhao B, Lin H, Jiang X, Li W, Gao Y, Li M, Yu Y, Chen N, Gao J. Exosome-like nanoparticles derived from fruits, vegetables, and herbs: innovative strategies of therapeutic and drug delivery, Theranostics, 14 (2024) 4598–4621. 10.7150/thno.97096 PMC11373634 39239509

[179] Yamasaki M, Yamasaki Y, Furusho R, Kimura H, Kamei I, Sonoda H, Ikeda M, Oshima T, Ogawa K, Nishiyama K. Onion (Allium cepa L.)-Derived Nanoparticles Inhibited LPS-Induced Nitrate Production, However, Their Intracellular Incorporation by Endocytosis Was Not Involved in This Effect on RAW264 Cells. Molecules. 2021;26:2763. 34067155 10.3390/molecules26092763 PMC8124543

[180] Sasaki D, Kusamori K, Takayama Y, Itakura S, Todo H, Nishikawa M. Development of nanoparticles derived from corn as mass producible bionanoparticles with anticancer activity. Sci Rep. 2021;11:22818. 34819568 10.1038/s41598-021-02241-y PMC8613273

[181] Liu C, Yan X, Zhang Y, Yang M, Ma Y, Zhang Y, Xu Q, Tu K, Zhang M. Oral administration of turmeric-derived exosome-like nanovesicles with anti-inflammatory and pro-resolving bioactions for murine colitis therapy. J Nanobiotechnol. 2022;20:206. 10.1186/s12951-022-01421-w PMC9052603 35488343

[182] Song H, Canup BSB, Ngo VL, Denning TL, Garg P, Laroui H. Internalization of Garlic-Derived Nanovesicles on Liver Cells is Triggered by Interaction With CD98. ACS Omega. 2020;5:23118–28. 32954162 10.1021/acsomega.0c02893 PMC7495725

[183] Itakura S, Shohji A, Amagai S, Kitamura M, Takayama K, Sugibayashi K, Todo H. Gene knockdown in HaCaT cells by small interfering RNAs entrapped in grapefruit-derived extracellular vesicles using a microfluidic device. Sci Rep. 2023;13:3102. 36813850 10.1038/s41598-023-30180-3 PMC9947018

[184] Cui C, Du M, Zhao Y, Tang J, Liu M, Min G, Chen R, Zhang Q, Sun Z, Weng H. Functional Ginger-Derived Extracellular Vesicles-Coated ZIF-8 Containing TNF-α siRNA for Ulcerative Colitis Therapy by Modulating Gut Microbiota. ACS Appl Mater Interfaces. 2024;16:53460–73. 39303016 10.1021/acsami.4c10562

[185] Zhuang X, Teng Y, Samykutty A, Mu J, Deng Z, Zhang L, Cao P, Rong Y, Yan J, Miller D, Zhang HG. Grapefruit-derived Nanovectors Delivering Therapeutic miR17 Through an Intranasal Route Inhibit Brain Tumor Progression, Molecular therapy: the journal of the American Society of Gene Therapy, 2016; 24: 96-105. 10.1038/mt.2015.188 PMC4754550 26444082

[186] Yepes-Molina L, Martínez-Ballesta MC, Carvajal M. Plant plasma membrane vesicles interaction with keratinocytes reveals their potential as carriers. J Adv Res. 2020;23:101–11. 32089878 10.1016/j.jare.2020.02.004 PMC7025959

[187] Date AA, Hanes J, Ensign LM. Nanoparticles for oral delivery: Design, evaluation and state-of-the-art. J Control Release. 2016;240:504–26. 27292178 10.1016/j.jconrel.2016.06.016 PMC5064878

[188] Mu J, Zhuang X, Wang Q, Jiang H, Deng ZB, Wang B, Zhang L, Kakar S, Jun Y, Miller D, Zhang HG. Interspecies communication between plant and mouse gut host cells through edible plant derived exosome-like nanoparticles. Volume 58. Molecular nutrition & food research; 2014. pp. 1561–73. 10.1002/mnfr.201300729 PMC4851829 24842810

[189] Niu W, Xiao Q, Wang X, Zhu J, Li J, Liang X, Peng Y, Wu C, Lu R, Pan Y, Luo J, Zhong X, He H, Rong Z, Fan JB, Wang Y. A Biomimetic Drug Delivery System by Integrating Grapefruit Extracellular Vesicles and Doxorubicin-Loaded Heparin-Based Nanoparticles for Glioma Therapy. Nano Lett. 2021;21:1484–92. 33475372 10.1021/acs.nanolett.0c04753

[190] Hang Z, Zhou L, Xing C, Wen Y, Du H. The blood-brain barrier, a key bridge to treat neurodegenerative diseases. Ageing Res Rev. 2023;91:102070. 37704051 10.1016/j.arr.2023.102070

[191] Wang Y, Wei Y, Liao H, Fu H, Yang X, Xiang Q, Zhang S. Plant Exosome-like Nanoparticles as Biological Shuttles for Transdermal Drug Delivery, Bioengineering (Basel, Switzerland), 10 (2023). 10.3390/bioengineering10010104 PMC9854743 36671676

[192] Abraham AM, Wiemann S, Ambreen G, Zhou J, Engelhardt K, Brüßler J, Bakowsky U, Li SM, Mandic R, Pocsfalvi G, Keck CM. Cucumber-Derived Exosome-like Vesicles and PlantCrystals for Improved Dermal Drug Delivery, Pharmaceutics, 14 (2022). 10.3390/pharmaceutics14030476 PMC8955785 35335851

[193] Jin Z, Na J, Lin X, Jiao R, Liu X, Huang Y. Plant-derived exosome-like nanovesicles: A novel nanotool for disease therapy. Heliyon. 2024;10:e30630. 38765146 10.1016/j.heliyon.2024.e30630 PMC11098843

[194] Chu K, Liu J, Zhang X, Wang M, Yu W, Chen Y, Xu L, Yang G, Zhang N, Zhao T. Herbal Medicine-Derived Exosome-Like Nanovesicles: A Rising Star in Cancer Therapy. Int J Nanomed. 2024;19:7585–603. 10.2147/IJN.S477270 PMC11287466 39081899

[195] Kim J, Li S, Zhang S, Wang J. Plant-derived exosome-like nanoparticles and their therapeutic activities. Asian J Pharm Sci. 2022;17:53–69. 35261644 10.1016/j.ajps.2021.05.006 PMC8888139

[196] Chen T, Ma B, Lu S, Zeng L, Wang H, Shi W, Zhou L, Xia Y, Zhang X, Zhang J, Chen J. Cucumber-Derived Nanovesicles Containing Cucurbitacin B for Non-Small Cell Lung Cancer Therapy. Int J Nanomed. 2022;17:3583–99. 10.2147/IJN.S362244 PMC9376005 35974872

[197] Yang L, Gu T, Xu Y, Liu Y, Zhang Y, Jiang Z, Peng L. Plant polysaccharides as novel biomaterials for microcapsule construction and therapeutics delivery. Int J Pharm. 2022;625:122137. 36029991 10.1016/j.ijpharm.2022.122137

[198] Hao S, Yang H, Hu J, Luo L, Yuan Y, Liu L. Bioactive compounds and biological functions of medicinal plant-derived extracellular vesicles. Pharmacol Res. 2024;200:107062. 38211637 10.1016/j.phrs.2024.107062

[199] Wei X, Li X, Zhang Y, Wang J, Shen S. Advances in the Therapeutic Applications of Plant-Derived Exosomes in the Treatment of Inflammatory Diseases. Volume 11. Biomedicines; 2023. 10.3390/biomedicines11061554 PMC10294895 37371649

[200] Cui L, Perini G, Palmieri V, De Spirito M, Papi M. Plant-Derived Extracellular Vesicles as a Novel Frontier in Cancer Therapeutics, Nanomaterials (Basel, Switzerland), 14 (2024). 10.3390/nano14161331 PMC11356885 39195369

[201] Huang Y, Liu H, Sun X, Ding M, Tao G, Li X. Honeysuckle-derived microRNA2911 directly inhibits varicella-zoster virus replication by targeting IE62 gene. J Neurovirol. 2019;25:457–63. 31140131 10.1007/s13365-019-00741-2

[202] Liu B, Lu Y, Chen X, Muthuraj PG, Li X, Pattabiraman M, Zempleni J, Kachman SD, Natarajan SK, Yu J. Protective Role of Shiitake Mushroom-Derived Exosome-Like Nanoparticles in D-Galactosamine and Lipopolysaccharide-Induced Acute Liver Injury in Mice, Nutrients, 12 (2020). 10.3390/nu12020477 PMC7071144 32069862

[203] Babbar R, Kaur A, Vanya R, Arora JK, Gupta P, Wal AK, Tripathi AA, Koparde P, Goyal S, Ramniwas M, Gulati T, Behl. Impact of Bioactive Compounds in the Management of Various Inflammatory Diseases. Curr Pharm Des. 2024;30:1880–93. 38818920 10.2174/0113816128299615240513174041

[204] Xie J, Xiong S, Li Y, Xia B, Li M, Zhang Z, Shi Z, Peng Q, Li C, Lin L, Liao D. Phenolic acids from medicinal and edible homologous plants: a potential anti-inflammatory agent for inflammatory diseases. Front Immunol. 2024;15:1345002. 38975345 10.3389/fimmu.2024.1345002 PMC11224438

[205] Kim ME, Lee JS. Molecular Foundations of Inflammatory Diseases: Insights into Inflammation and Inflammasomes. Curr Issues Mol Biol. 2024;46:469–84. 38248332 10.3390/cimb46010030 PMC10813887

[206] Kim J, Zhang S, Zhu Y, Wang R, Wang J. Amelioration of colitis progression by ginseng-derived exosome-like nanoparticles through suppression of inflammatory cytokines. J ginseng Res. 2023;47:627–37. 37720571 10.1016/j.jgr.2023.01.004 PMC10499592

[207] Kawada K, Ishida T, Morisawa S, Jobu K, Higashi Y, Aizawa F, Yagi K, Izawa-Ishizawa Y, Niimura T, Abe S, Goda M, Miyamura M, Ishizawa K. Atractylodes lancea (Thunb.) DC. [Asteraceae] rhizome-derived exosome-like nanoparticles suppress lipopolysaccharide-induced inflammation in murine microglial cells. Front Pharmacol. 2024;15:1302055. 38738173 10.3389/fphar.2024.1302055 PMC11082290

[208] Liu Y, Ahumada AL, Bayraktar E, Schwartz P, Chowdhury M, Shi S, Sebastian MM, Khant H, de Val N, Bayram NN, Zhang G, Vu TC, Jie Z, Jennings NB, Rodriguez-Aguayo C, Swain J, Stur E, Mangala LS, Wu Y, Nagaraju S, Ermias B, Li C, Lopez-Berestein G, Braam J, Sood AK. Enhancing oral delivery of plant-derived vesicles for colitis. J Control Release. 2023;357:472–83. 37031740 10.1016/j.jconrel.2023.03.056 PMC10191613

[209] Ding L, Chang CJ, Liang ML, Dong KM, Li FR. Plant-Derived Extracellular Vesicles as Potential Emerging Tools for Cancer Therapeutics. Adv Ther. 2024;7:2400256.

[210] Yan G, Xiao Q, Zhao J, Chen H, Xu Y, Tan M, Peng L. Brucea javanica derived exosome-like nanovesicles deliver miRNAs for cancer therapy. J Control Release. 2024;367:425–40. 38295998 10.1016/j.jconrel.2024.01.060

[211] Yang M, Guo J, Li J, Wang S, Sun Y, Liu Y, Peng Y. Platycodon grandiflorum-derived extracellular vesicles suppress triple-negative breast cancer growth by reversing the immunosuppressive tumor microenvironment and modulating the gut microbiota. J Nanobiotechnol. 2025;23:92. 10.1186/s12951-025-03139-x PMC11804104 39920791

[212] Kim DK, Kang SJ, Rhee WJ. Perilla-Leaf-Derived Extracellular Vesicles Selectively Inhibit Breast Cancer Cell Proliferation and Invasion. Int J Mol Sci. 2023;24:15633. 37958616 10.3390/ijms242115633 PMC10647566

[213] Yang X, Peng Y, Wang Y-e, Zheng Y, He Y, Pan J, Liu N, Xu Y, Ma R, Zhai J, Ma Y, Guan S. Curcumae Rhizoma Exosomes-like nanoparticles loaded Astragalus components improve the absorption and enhance anti-tumor effect. J Drug Deliv Sci Technol. 2023;81:104274.

[214] Yang M, Xu L, Wang W. Molecular anti-tumorigenic mechanism of Radix Polygoni Multiflori-derived exosome-like nanoparticles. Heliyon. 2025;11:e41918. 40028544 10.1016/j.heliyon.2025.e41918 PMC11868940

[215] Ma X, Chen N, Zeng P, He Y, Zhang T, Lu Y, Li Z, Xu J, You J, Zheng Y, Wang L, Luo M, Wu J. Hypericum Perforatum-Derived Exosomes-Like Nanovesicles: A Novel Natural Photosensitizer for Effective Tumor Photodynamic Therapy. Int J Nanomed. 2025;20:1529–41. 10.2147/IJN.S510339 PMC11806729 39925681

[216] Cui L, Perini G, Augello A, Palmieri V, De Spirito M, Papi M. Plant-derived extracellular nanovesicles: a promising biomedical approach for effective targeting of triple negative breast cancer cells. Front Bioeng Biotechnol. 2024;12:1390708. 38952670 10.3389/fbioe.2024.1390708 PMC11215178

[217] Lu X, Han Q, Chen J, Wu T, Cheng Y, Li F, Xia W. Celery (Apium graveolens L.) Exosome-like Nanovesicles as a New-Generation Chemotherapy Drug Delivery Platform against Tumor Proliferation. J Agric Food Chem. 2023;71:8413–24. 37222554 10.1021/acs.jafc.2c07760

[218] Han X, Wei Q, Lv Y, Weng L, Huang H, Wei Q, Li M, Mao Y, Hua D, Cai X, Cao M, Cao P. Ginseng-derived nanoparticles potentiate immune checkpoint antibody efficacy by reprogramming the cold tumor microenvironment. Mol therapy: J Am Soc Gene Therapy. 2022;30:327–40. 10.1016/j.ymthe.2021.08.028 PMC8753455 34450250

[219] Wu W, Zhang B, Wang W, Bu Q, Li Y, Zhang P, Zeng L. Plant-Derived Exosome-Like Nanovesicles in Chronic Wound Healing. Int J Nanomed. 2024;19:11293–303. 10.2147/IJN.S485441 PMC11549884 39524918

[220] Wang Q, Liu K, Cao X, Rong W, Shi W, Yu Q, Deng W, Yu J, Xu X. Plant-derived exosomes extracted from Lycium barbarum L. loaded with isoliquiritigenin to promote spinal cord injury repair based on 3D printed bionic scaffold. Bioeng translational Med. 2024;9:e10646. 10.1002/btm2.10646 PMC11256167 39036078

[221] Natania F, Iriawati I, Ayuningtyas FD, Barlian A. Potential of Plant-derived Exosome-like Nanoparticles from Physalis peruviana Fruit for Human Dermal Fibroblast Regeneration and Remodeling. Pharm Nanotechnol. 2025;13:358–71. 38243927 10.2174/0122117385281838240105110106

[222] Tan S, Liu Z, Cong M, Zhong X, Mao Y, Fan M, Jiao F, Qiao H. Dandelion-derived vesicles-laden hydrogel dressings capable of neutralizing Staphylococcus aureus exotoxins for the care of invasive wounds. J Control Release. 2024;368:355–71. 38432468 10.1016/j.jconrel.2024.02.045

[223] Valentino A, Conte R, Bousta D, Bekkari H, Di Salle A, Calarco A, Peluso G. Extracellular Vesicles Derived from Opuntia ficus-indica Fruit (OFI-EVs) Speed Up the Normal Wound Healing Processes by Modulating Cellular Responses. Int J Mol Sci, 2024; 25: 7103. 10.3390/ijms25137103 PMC11241772 39000212

[224] Saroj S, Saha S, Ali A, Gupta SK, Bharadwaj A, Agrawal T, Pal S, Rakshit T. Plant Extracellular Nanovesicle-Loaded Hydrogel for Topical Antibacterial Wound Healing In Vivo, ACS applied bio materials, 8 (2025) 1–11. 10.1021/acsabm.4c00992 39377525

[225] Pan Y, Tan WF, Yang MQ, Li JY, Geller DA. The therapeutic potential of exosomes derived from different cell sources in liver diseases. Am J Physiol Gastrointest Liver Physiol. 2022;322:G397–404. 35107032 10.1152/ajpgi.00054.2021 PMC8917924

[226] Kim JS, Kim DH, Gil MC, Kwon HJ, Seo W, Kim DK, Cho YE. Pomegranate-Derived Exosome-Like Nanovesicles Alleviate Binge Alcohol-Induced Leaky Gut and Liver Injury. J Med Food. 2023;26:1–10. 37733268 10.1089/jmf.2023.K.0060

[227] Kim JS, Eom JY, Kim HW, Ko JW, Hong EJ, Kim MN, Kim J, Kim DK, Kwon HJ, Cho YE. Hemp sprout-derived exosome-like nanovesicles as hepatoprotective agents attenuate liver fibrosis. Biomater Sci. 2024;12:5361–71. 39253746 10.1039/d4bm00812j

[228] Ismail M, Liu J, Wang N, Zhang D, Qin C, Shi B, Zheng M. Advanced nanoparticle engineering for precision therapeutics of brain diseases. Biomaterials. 2025;318:123138. 39914193 10.1016/j.biomaterials.2025.123138

[229] Li J, Zhang H, Jiang Y, Li N, Zhu A, Zhang Y, Feng K, Zeng W, Di L, Wang R. The landscape of extracellular vesicles combined with intranasal delivery towards brain diseases. Nano Today. 2024;55:102169.

[230] Wu J, Ma L, Sun D, Zhang X, Cui J, Du Y, Guo Y, Wang X, Di L, Wang R. Bioengineering extracellular vesicles as novel nanocarriers towards brain disorders. Nano Res. 2022;16:2635–59.

[231] Xu Y, Yan G, Zhao J, Ren Y, Xiao Q, Tan M, Peng L. Plant-derived exosomes as cell homogeneous nanoplatforms for brain biomacromolecules delivery ameliorate mitochondrial dysfunction against Parkinson’s disease. Nano Today. 2024;58:102438.

[232] Zhang Y, Lu L, Li Y, Liu H, Zhou W, Zhang L. Response Surface Methodology Optimization of Exosome-like Nanovesicles Extraction from Lycium ruthenicum Murray and Their Inhibitory Effects on Aβ-Induced Apoptosis and Oxidative Stress in HT22 Cells, Foods (Basel, Switzerland), 13 (2024). 10.3390/foods13203328 PMC11507227 39456390

[233] Cai H, Huang LY, Hong R, Song JX, Guo XJ, Zhou W, Hu ZL, Wang W, Wang YL, Shen JG, Qi SH. Momordica charantia Exosome-Like Nanoparticles Exert Neuroprotective Effects Against Ischemic Brain Injury via Inhibiting Matrix Metalloproteinase 9 and Activating the AKT/GSK3β Signaling Pathway. Front Pharmacol. 2022;13:908830. 35814200 10.3389/fphar.2022.908830 PMC9263912

[234] Sundaram K, Mu J, Kumar A, Behera J, Lei C, Sriwastva MK, Xu F, Dryden GW, Zhang L, Chen S, Yan J, Zhang X, Park JW, Merchant ML, Tyagi N, Teng Y, Zhang HG. Garlic exosome-like nanoparticles reverse high-fat diet induced obesity via the gut/brain axis. Theranostics. 2022;12:1220–46. 35154484 10.7150/thno.65427 PMC8771565

[235] Dong X, Liu H, Yuan D, Gulati K, Liu Y. Re-engineering bone: pathogenesis, diagnosis and emerging therapies for osteoporosis. J Mater Chem B. 2025;13:4938–63. 40192254 10.1039/d4tb02628d

[236] Chen X, Wu W, Zhu W, Zhou J, Chen J, Lin Z, Zhang S, Caruso F, Liu C. Regulation of Bone Remodeling by Metal-Phenolic Networks for the Treatment of Systemic Osteoporosis. ACS Appl Mater Interfaces. 2025;17:5995–6008. 39818714 10.1021/acsami.4c18829 PMC11788982

[237] Mehrvar A, Akbari M, Khosroshahi EM, Nekavand M, Mokhtari K, Baniasadi M, Aghababaian M, Karimi M, Amiri S, Moazen A, Maghsoudloo M, Alimohammadi M, Rahimzadeh P, Farahani N, Vaghar ME, Entezari M, Hashemi M. The impact of exosomes on bone health: A focus on osteoporosis. Pathol Res Pract. 2024;263:155618. 39362132 10.1016/j.prp.2024.155618

[238] Hwang JH, Park YS, Kim HS, Kim DH, Lee SH, Lee CH, Lee SH, Kim JE, Lee S, Kim HM, Kim HW, Kim J, Seo W, Kwon HJ, Song BJ, Kim DK, Baek MC, Cho YE. Yam-derived exosome-like nanovesicles stimulate osteoblast formation and prevent osteoporosis in mice. J Control Release. 2023;355:184–98. 36736431 10.1016/j.jconrel.2023.01.071

[239] Zhao Q, Feng J, Liu F, Liang Q, Xie M, Dong J, Zou Y, Ye J, Liu G, Cao Y, Guo Z, Qiao H, Zheng L, Zhao K. Rhizoma Drynariae-derived nanovesicles reverse osteoporosis by potentiating osteogenic differentiation of human bone marrow mesenchymal stem cells via targeting ERα signaling. Acta Pharm Sinica B. 2024;14:2210–27. 10.1016/j.apsb.2024.02.005 PMC11119514 38799625

[240] Zhan W, Deng M, Huang X, Xie D, Gao X, Chen J, Shi Z, Lu J, Lin H, Li P. Pueraria lobata-derived exosome-like nanovesicles alleviate osteoporosis by enhacning autophagy. J Control Release. 2023;364:644–53. 37967723 10.1016/j.jconrel.2023.11.020

[241] Fiorucci S, Urbani G, Biagioli M, Sepe V, Distrutti E, Zampella A. Bile acids and bile acid activated receptors in the treatment of Covid-19. Biochem Pharmacol. 2024;228:115983. 38081371 10.1016/j.bcp.2023.115983

[242] Valenzuela-Fernández A, Cabrera-Rodriguez R, Ciuffreda L, Perez-Yanes S, Estevez-Herrera J, González-Montelongo R, Alcoba-Florez J, Trujillo-González R, García-Martínez D, de Artola H, Gil-Campesino O, Díez-Gil JM, Lorenzo-Salazar C, Flores J. Garcia-Luis, Nanomaterials to combat SARS-CoV-2: Strategies to prevent, diagnose and treat COVID-19. Front Bioeng Biotechnol. 2022;10:1052436. 36507266 10.3389/fbioe.2022.1052436 PMC9732709

[243] Kalarikkal SP, Sundaram GM. Edible plant-derived exosomal microRNAs: Exploiting a cross-kingdom regulatory mechanism for targeting SARS-CoV-2. Toxicol Appl Pharmacol. 2021;414:115425. 33516820 10.1016/j.taap.2021.115425 PMC7844364

[244] Zhou LK, Zhou Z, Jiang XM, Zheng Y, Chen X, Fu Z, Xiao G, Zhang CY, Zhang LK, Yi Y. Absorbed plant MIR2911 in honeysuckle decoction inhibits SARS-CoV-2 replication and accelerates the negative conversion of infected patients. Cell discovery. 2020;6:54. 32802404 10.1038/s41421-020-00197-3 PMC7406496

[245] Sundaram K, Miller DP, Kumar A, Teng Y, Sayed M, Mu J, Lei C, Sriwastva MK, Zhang L, Yan J, Merchant ML, He L, Fang Y, Zhang S, Zhang X, Park JW, Lamont RJ, Zhang HG. Plant-Derived Exosomal Nanoparticles Inhibit Pathogenicity of Porphyromonas gingivalis, iScience, 21 (2019) 308–27. 10.1016/j.isci.2019.10.032 PMC6838522 31678913

[246] Cui Z, Liu T, Wen Y, Li W, Xu J, Chen Y, Chen D, Zhu Y. Oral administration of cranberry-derived exosomes attenuates murine premature ovarian failure in association with changes in the specific gut microbiota and diminution in ovarian granulosa cell PANoptosis. Food Funct. 2024;15:11697–714. 39530911 10.1039/d4fo03468f

[247] Ye C, Yan C, Bian SJ, Li XR, Li Y, Wang KX, Zhu YH, Wang L, Wang YC, Wang YY, Li TS, Qi SH, Luo L. Momordica charantia L.-derived exosome-like nanovesicles stabilize p62 expression to ameliorate doxorubicin cardiotoxicity. J Nanobiotechnol. 2024;22:464. 10.1186/s12951-024-02705-z PMC11297753 39095755

[248] Huang Z, Whitehead B, Nejsum P, Corredig M, Rasmussen MK. Tomato-derived extracellular vesicles increase intestinal zinc transportation by potentially down-regulating the expression of the metallothionein family. Food Res Int (Ottawa Ont). 2025;203:115804. 10.1016/j.foodres.2025.115804 40022334

[249] Mathieu M, Martin-Jaular L, Lavieu G, Théry C. Specificities of secretion and uptake of exosomes and other extracellular vesicles for cell-to-cell communication. Nat Cell Biol. 2019;21:9–17. 30602770 10.1038/s41556-018-0250-9

[250] Lian MQ, Chng WH, Liang J, Yeo HQ, Lee CK, Belaid M, Tollemeto M, Wacker MG, Czarny B, Pastorin G. Plant-derived extracellular vesicles: Recent advancements and current challenges on their use for biomedical applications. J Extracell vesicles. 2022;11:e12283. 36519808 10.1002/jev2.12283 PMC9753580

[251] Liu X, Mei L, Wang J, Liu X, Yang Y, Wu Z, Ji Y. Cutting-edge insights into the mechanistic understanding of plant-derived exosome-like nanoparticles: Implications for intestinal homeostasis. Food Res Int. 2025;208:116186. 40263791 10.1016/j.foodres.2025.116186

[252] Liu R, Zhang F, He X, Huang K. Plant Derived Exosome-Like Nanoparticles and Their Therapeutic Applications in Glucolipid Metabolism Diseases. J Agric Food Chem. 2025;73:6385–99. 40048449 10.1021/acs.jafc.4c12480

[253] Yi C, Lu L, Li Z, Guo Q, Ou L, Wang R, Tian X. Plant-derived exosome-like nanoparticles for microRNA delivery in cancer treatment. Drug delivery translational Res. 2025;15:84–101. 10.1007/s13346-024-01621-x 38758499

[254] Liu Y, Qi H, Zong J, Li M, Yang Y, Li X, Li T, Cho JY, Yu T. Oral Piwi-Interacting RNA Delivery Mediated by Green Tea-Derived Exosome-Like Nanovesicles for the Treatment of Aortic Dissection. Adv Healthc Mater. 2024;13:e2401466. 39087398 10.1002/adhm.202401466

[255] Li Y, Wang Y, Zhao H, Pan Q, Chen G. Engineering Strategies of Plant-Derived Exosome-Like Nanovesicles: Current Knowledge and Future Perspectives. Int J Nanomed. 2024;19:12793–815. 10.2147/IJN.S496664 PMC11618857 39640047

[256] Zhang M, Hu S, Liu L, Dang P, Liu Y, Sun Z, Qiao B, Wang C. Engineered exosomes from different sources for cancer-targeted therapy. Signal Transduct Target Ther. 2023;8:124. 36922504 10.1038/s41392-023-01382-y PMC10017761

[257] Dong Y, Wan J, Xiao H, Wang J, Yu X, Liu Y, Lei S, Zhang Z, Zhang L, Yang Q, Wang S. MSCs derived membrane coating nanoparticles targeted delivery itaconic acid to regulate M1 macrophage pyroptosis for osteoarthritis therapy. Appl Mater Today. 2024;39:102314.

[258] Han R, Zhou D, Ji N, Yin Z, Wang J, Zhang Q, Zhang H, Liu J, Liu X, Liu H, Han Q, Su J. Folic acid-modified ginger-derived extracellular vesicles for targeted treatment of rheumatoid arthritis by remodeling immune microenvironment via the PI3K-AKT pathway. J Nanobiotechnol. 2025;23:41. 10.1186/s12951-025-03096-5 PMC11756199 39849554

[259] Long F, Pan Y, Li J, Sha S, Shi X, Guo H, Huang C, Xiao Q, Fan C, Zhang X, Fan JB, Wang Y. Orange-derived extracellular vesicles nanodrugs for efficient treatment of ovarian cancer assisted by transcytosis effect. Acta Pharm Sinica B. 2023;13:5121–34. 10.1016/j.apsb.2023.04.006 PMC10692363 38045062

[260] Guo Z, Zhang Y, Gong Y, Li G, Pan J, Dou D, Ma K, Cui C, Liu Y, Zhu X. Antibody functionalized curcuma-derived extracellular vesicles loaded with doxorubicin overcome therapy-induced senescence and enhance chemotherapy. J Control Release. 2025;379:377–89. 39814319 10.1016/j.jconrel.2025.01.029

[261] Zhang Y, Qiu L, Li H, Cai W, Liu E, Zhang H, Muhitdinov B, Liang J, Huang Y. Biomimetically engineered plant-derived exosomes-like nanovesicles for rheumatoid arthritis therapy. Chin Chem Lett. 2025;36:110658.

[262] Hargreaves SM, Raposo A, Saraiva A, Zandonadi RP. Vegetarian Diet: An Overview through the Perspective of Quality of Life Domains. Int J Environ Res Public Health. 2021;18:4067. 33921521 10.3390/ijerph18084067 PMC8069426

[263] Han Y, Guo X, Ji Z, Guo Y, Ma W, Du H, Guo Y, Xiao H. Colon health benefits of plant-derived exosome-like nanoparticles via modulating gut microbiota and immunity. Crit Rev Food Sci Nutr. 2025;65:7718–38. 40105379 10.1080/10408398.2025.2479066

[264] Pinedo M, de la Canal L, de Marcos Lousa C. A call for Rigor and standardization in plant extracellular vesicle research. J Extracell vesicles. 2021;10:e12048. 33936567 10.1002/jev2.12048 PMC8077130

[265] Jung D, Kim NE, Kim S, Bae JH, Jung IY, Doh KW, Lee B, Kim DK, Cho YE, Baek MC. Plant-derived nanovesicles and therapeutic application. Pharmacol Ther. 2025;269:108832. 40023319 10.1016/j.pharmthera.2025.108832

[266] Liebana-Jordan M, Brotons B, Falcon-Perez JM, Gonzalez E. Extracellular Vesicles in the Fungi Kingdom. Int J Mol Sci. 2021;22:28. 10.3390/ijms22137221 PMC8269022 34281276

[267] Rodrigues ML, May RC, Janbon G. The multiple frontiers in the study of extracellular vesicles produced by fungi. Microbes Infect. 2024;26:105233. 37805124 10.1016/j.micinf.2023.105233

[268] Xu X, Xu LM, Wen CN, Zhang YM, Xia J, Liang YJ. Engineering bacterial membrane vesicles: A new paradigm in biomedical innovation. Coord Chem Rev. 2025;543:22.

[269] Li Q, Chen X, Xie J, Nie S. Engineered Bacterial Extracellular Besicles: Developments, Challenges, and Opportunities, Engineering, (2025).

[270] Zhao B, Wei JP, Jiang ZJ, Long YM, Xu Y, Jiang BT. Mesenchymal stem cell-derived exosomes: an emerging therapeutic strategy for hepatic ischemia-reperfusion injury. Stem Cell Res Ther. 2025;16:13. 40229893 10.1186/s13287-025-04302-9 PMC11998454

[271] Quan JL, Liu Q, Li PH, Yang ZY, Zhang YH, Zhao FX, Zhu GH. Mesenchymal stem cell exosome therapy: current research status in the treatment of neurodegenerative diseases and the possibility of reversing normal brain aging. Stem Cell Res Ther. 2025;16:13. 39985030 10.1186/s13287-025-04160-5 PMC11846194

[272] Zheng YZ, Wang TR, Zhang JX, Wei S, Wu ZJ, Li JL, Shi BH, Sun ZX, Xu WR, Zhu J. Plant-Derived Nanovesicles: A Promising Frontier in Tissue Repair and Antiaging. J Agric Food Chem. 2025;73:13159–77. 40402864 10.1021/acs.jafc.5c01547 PMC12147150

